# Neurofibromin level directs RAS pathway signaling and mediates sensitivity to targeted agents in malignant peripheral nerve sheath tumors

**DOI:** 10.18632/oncotarget.25181

**Published:** 2018-04-27

**Authors:** Elliot John Kahen, Andrew Brohl, Diana Yu, Darcy Welch, Christopher L. Cubitt, Jae K. Lee, Yunyun Chen, Sean J. Yoder, Jamie K. Teer, Yonghong O. Zhang, Margaret R. Wallace, Damon R. Reed

**Affiliations:** ^1^ Sunshine Project Translational Research Lab, Tampa, FL 33612, Florida, USA; ^2^ Sarcoma Department, Tampa, FL 33612, Florida, USA; ^3^ Department of Biostatistics and Bioinformatics Tampa, FL 33612, Florida, USA; ^4^ Molecular Genomics Core Facility, Tampa, FL 33612, Florida, USA; ^5^ Department of Molecular Genetics and Microbiology and UF Health Cancer Center, University of Florida, Tampa, FL 33612, Florida, USA; ^6^ Chemical Biology and Molecular Medicine Program, Tampa, FL 33612, Florida, USA; ^7^ Adolescent and Young Adult Program, H. Lee Moffitt Cancer Center and Research Institute, Tampa, FL 33612, Florida, USA

**Keywords:** NF1, MPNST, RAS, combination therapy, MEK

## Abstract

Malignant peripheral nerve sheath tumor (MPNST) is a type of soft-tissue sarcoma strongly associated with dysfunction in neurofibromin; an inhibitor of the RAS pathway. We performed high-throughput screening of an array of FDA approved and promising agents in clinical development both alone and in combination at physiologically achievable concentrations against a panel of established MPNST cell line models. We found that drugs targeting a variety of factors in the RAS pathway can effectively lead to cell death *in vitro* with considerable drug combination synergy in regimens that target MEK or mTOR. We observed that the degree of relative sensitivity to chemotherapeutic agents was associated with the status of neurofibromin in these cell line models. Using a combination of agents that target MEK and mTORC1/2, we effectively silenced RAS/PI3K/MEK/mTOR signaling *in vitro*. Moreover, we employed RNAi against *NF1* to establish that MPNST drug sensitivity is directly proportional to relative level of intracellular neurofibromin. Thus, two-drug combinations that target MEK and mTORC1/2 are most effective in halting the RAS signaling cascade, and the relative success of this and related small molecule interventions in MPNSTs may be predicated upon the molecular status of neurofibromin.

## INTRODUCTION

Inactivating mutations in a copy of the *NF1* gene cause neurofibromatosis type 1 (NF1), an autosomal dominant condition characterized by formation of benign tumors and an increased risk of developing Malignant Peripheral Nerve Sheath Tumors (MPNST) [1–3]. MPNST is a devastating sarcoma subtype with 5-year, 10-year, and metastatic survival rates of approximately 50%, 30%, and 8%, respectively [4, 5]. Unresectable MPNSTs remain relatively refractory to chemotherapies despite recent advances, particularly when arising in NF1 patients [6].

The *NF1* gene encodes the protein neurofibromin that functions as a negative regulator of RAS signaling through its GTPase activating protein (GAP) activity, converting active RAS-GTP to inactive RAS-GDP, which is essential for the regulation of cell proliferation and differentiation [7–9]. Consequently, inactivating mutations in *NF1* lead to elevated levels of RAS-GTP and thus increased RAS signaling [10, 11]. Both benign neurofibromas (Schwann cell tumors) and MPNSTs (also Schwann lineage) are thought to follow the two-hit mechanism, where the initiating tumor cell has most commonly lost the other *NF1* allele by somatic mutation, rendering the cell deficient in neurofibromin activity. Because such cells have increased RAS activity, most approaches to developing treatments for MPNSTs have been focused on inhibiting targets downstream of RAS such as pathways associated with mTOR or MEK/MAPK [12–15]. Effective targeting of RAS activation directly has proven elusive despite large efforts in other RAS driven malignancies such as colorectal carcinoma and pancreatic adenocarcinoma [16, 17]. This has been in part due to the difficulty in directly targeting the three major isoforms of RAS (HRAS, KRAS and NRAS), clinical toxicity when inhibiting multiple downstream pathways of RAS, and the lack of a clear single node to block [12, 13, 18–21].

Utilizing an array of FDA approved drugs or agents in clinical development that included current standard of care for MPNST and those that target factors downstream of RAS, we sought to evaluate if these small molecules, alone or in combination, could not only reduce downstream RAS signaling in cell line models of MPNST, but also whether that blockade would lead to cell death to support further translation towards clinical trials. Moreover, we sought to examine the association of residual neurofibromin with the phosphorylation cascade downstream of RAS and determine the role played in the sensitivity of MPNST cells to chemotherapeutic agents. This study is novel in its approach to interpreting drug screening results in the context of genomic findings revealing a neurofibromin level contextual mechanism for RAS pathway drug sensitivity in MPNSTs.

## RESULTS

### MPNSTs are variably sensitive to individual chemotherapeutic agents at clinically achievable concentrations

**Figure 1:**
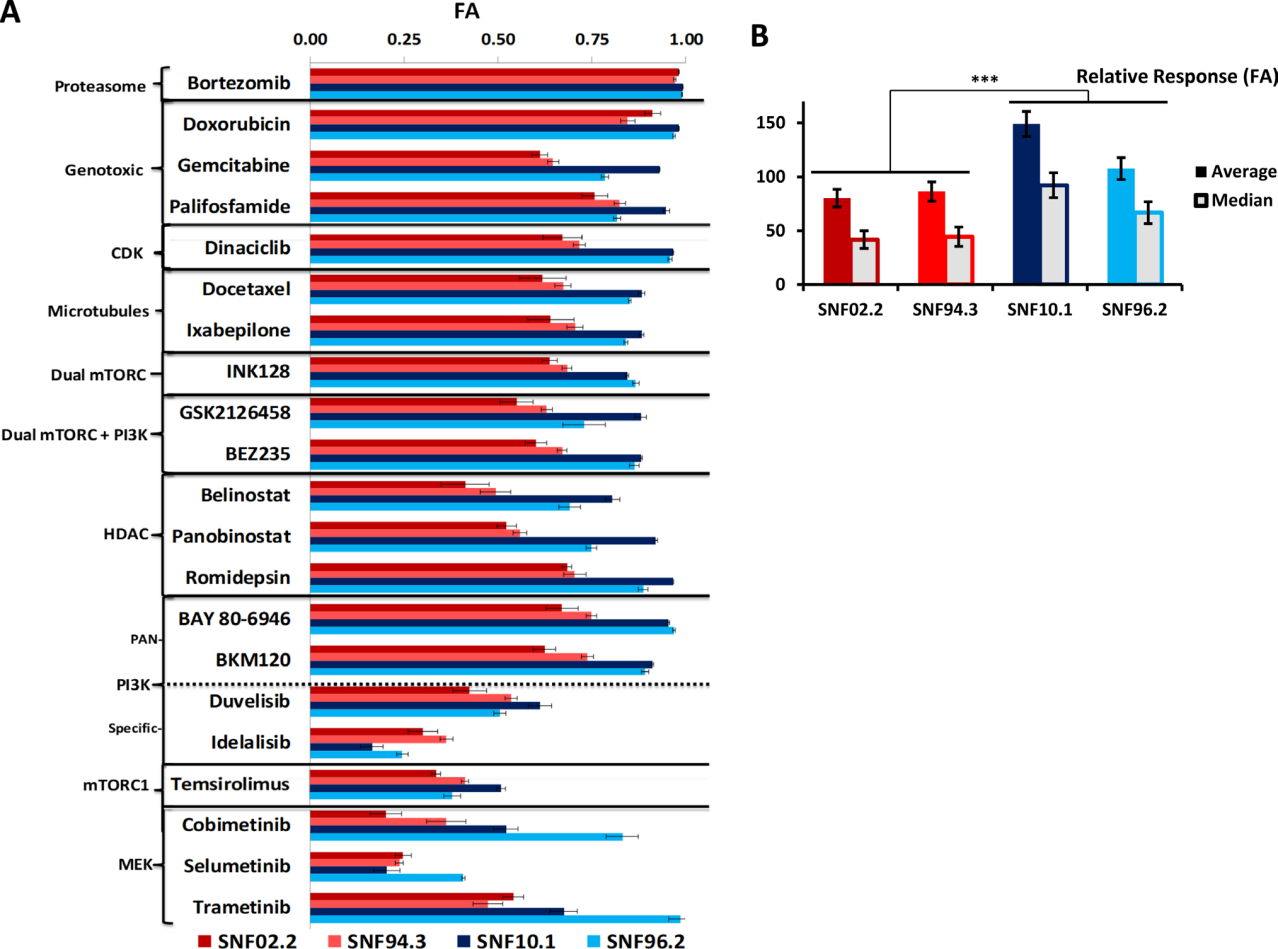
Results of single agent screening in MPNST cell lines (**A**) FA values of selected high performing agents and putative RAS-pathway inhibitors in 4 MPNST cell lines at Cmax. (**B**) Contrast of relative response to all drugs tested between cell lines, ^***^P < 0.00001. Error bars represent SEM. See Supplementary Table 1 and Supplementary Figure 1 for all single agent data.

**Figure 2:**
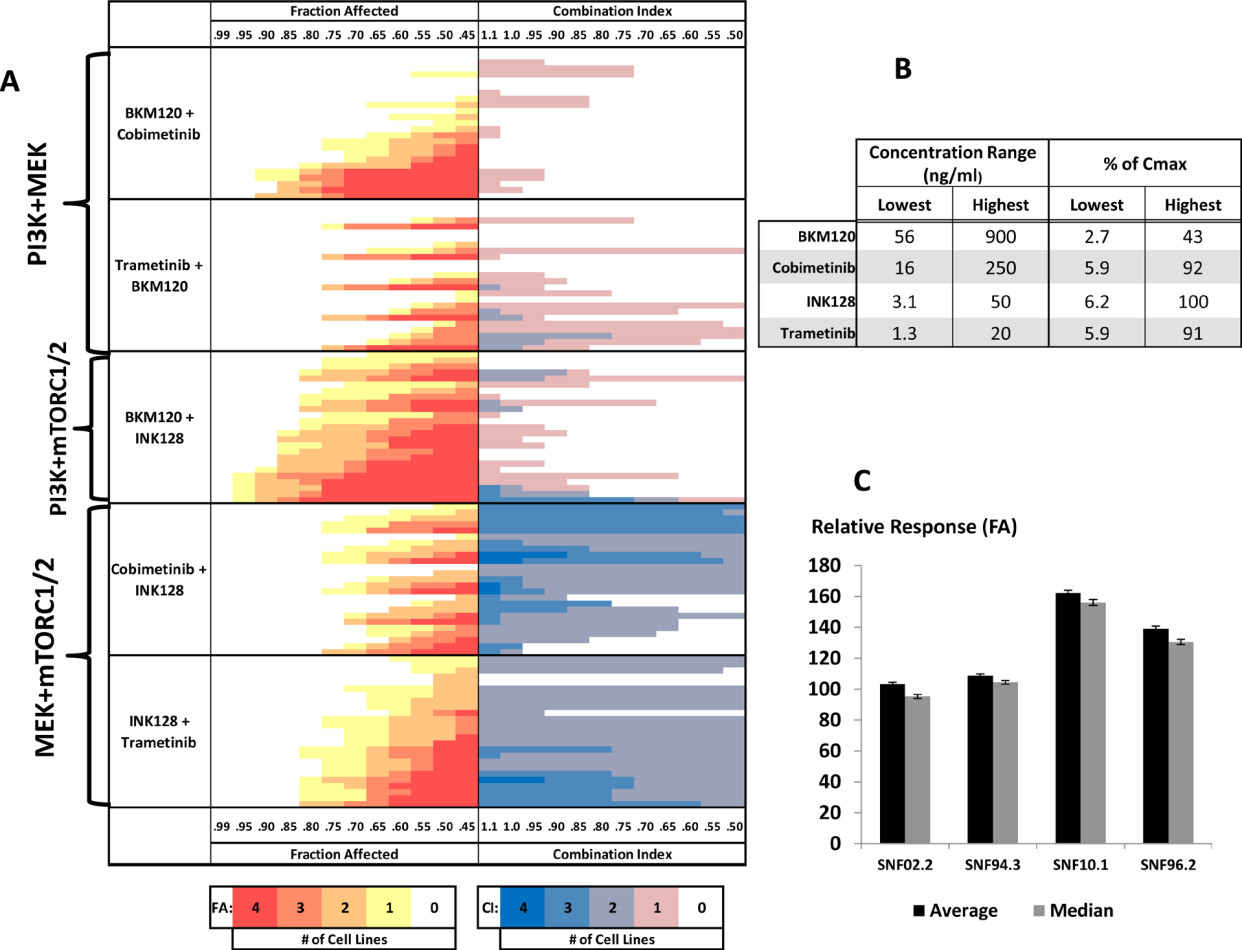
Drug-combination screening in MPNST cell line models (**A**) FA-CI heat maps for two-drug combinations targeting PI3K+MEK, PI3K+mTORC1/2, and MEK+mTORC1/2 from top to bottom, respectively. Drug concentrations increase from top to bottom (**B**, Lowest to Highest). The color of each bar represents the number of cell lines meeting the given criteria for FA or CI (see color bars below and horizontal axes on top and bottom). FA panels (red, orange, and yellow): bars further left indicate higher FA in response to treatment. Darker red signifies more cell lines meeting the denoted FA. CI panels (blue and pink): bars further right indicate lower CI/greater synergism between drug combinations. Darker blue signifies percentage greater number of cell lines meeting the denoted CI. The relative importance of each drug in a combination can be determined by the pattern of the heatmap. A right triangle-like pattern with a mostly flat hypotenuse implies that the drugs are contributing mostly equally to the response. A stepwise increase in signal, starting with little or no color with a sudden burst follow down suggests that, for each Drug A + Drug B, Drug A is more greatly contributing to the response. Peaks and valleys imply that Drug B is more greatly contributing to the response. Drug treatment was concurrent. (**C**) Comparison of average and median relative response values for all tested drug combinations across each cell line.

We screened 59 agents with diverse mechanisms of action at multiple clinically achievable concentrations with the use of high throughput assays of cell viability with CT-Glo, which provides a luminescence signal in proportion to ATP concentration (Supplementary Table 1). Fraction affected (FA), obtained by normalizing the luminescence signal of a treatment condition against an untreated control condition on the same plate, is reported. The agents leading to the top 25% FA were mostly clinically used cytotoxic agents, RAS pathway targeting agents, HDAC’s, and microtubule inhibitors (Figure 1A, Supplementary Table 2). The current standard of care, doxorubicin, performed second best following the proteasome inhibitor bortezomib, both demonstrating FA values over 0.90 (equivalent to 90% cell death) on average across the 4 cell lines at the top concentrations tested. The alkylating agent palifosfamide, an analogue of ifosfamide, frequently used with doxorubicin clinically in the treatment of MPNST [22] was also active with an average FA above 0.80. Importantly, 6 drugs that impact the RAS pathway members MEK1/2, mTORC1/2, and PI3K (pan-Class I) produced FA's of 0.70–0.85 at the top concentrations tested. These agents, in order of efficacy, are: BAY 80-6946 (copanlisib), BKM-120 (buparlisib), TAK-228 (INK-128), BEZ235 (dactolisib), GSK2126458 (omipalisib), and trametinib. Agents with specificity towards one or two PI3K members – idelalisib and duvelisib, which target the δ and δ&γ isoforms, respectively – had relatively poor activity. Additionally, our screen presented 3 HDAC inhibitors in the top 25% most active agents (romidepsin, panobinostat, and belinostat). Other high activity drug classes include microtubule inhibitors (ixabepilone and docetaxel), the CDK1/2/9 inhibitor dinaciclib, and the nucleoside analogue, gemcitabine. Notably, where our single agent data overlap, results were highly concordant with a recent study [23]. Additional past *in vitro* and animal studies have identified several of these agents – bortezomib [24], gemcitabine [25], romidepsin [26], and trametinib [27] – as promising candidates in the treatment of MPNST. Interestingly, when assessing cell line specific response to individual chemotherapeutic agents, a trend became apparent, particularly when focusing on drugs that target factors in the RAS/PI3K/mTOR axis (Figure 2A). Upon calculation of the median and average across all FA values observed in the analysis (Figure 1B), a significant difference in response was observed between two groups of cell lines - SNF02.2 and SNF94.3 compared to SNF10.1 and SNF96.2 (*p <* 0.00001, Mann–Whitney–Wilcoxon test).

### Inhibitors of factors downstream of RAS act synergistically to reduce viability of MPNST cells

From amongst the active single agents, we focused on those that either demonstrated high efficacy or acted upon targets of the RAS pathway. Seventy-two two-drug combinations were tested at 25 concentrations across the 4 MPNST cell lines. (Figure 2A, Supplementary Table 3). All drugs were assessed at or below published Cmax values (Figure 2B). Broadly, following the trend observed in the single agent studies, SNF02.2 and SNF94.3 demonstrated similar tolerances with median FA values of 0.49 and 0.41, respectively. SNF10.1 and SNF96.2 were overall more sensitive to the tested treatments with median FA values of 0.82 and 0.62, respectively (Figure 2C).

The efficacy and synergy for two-drug combinations against MPNST cell lines fell into a few distinct groups. The most highly synergistic combinations contained HDAC, mTOR, MEK, PI3K, inhibitors. Of particular interest clinically are combinations that demonstrate strong synergism and efficacy at lower concentrations relative to Cmax. Translating such agents to the clinic could minimize toxicity and maximize the combination opportunities. The strongest synergism observed in this study involved combinations that included the dual pan-PI3K/mTORC1/2 inhibitor GSK2126458 (omipalisib) and the HDAC inhibitor romidepsin. Though certainly active, we chose not to follow-up with GSK2126458 due to clinical evidence that the drug cannot be tolerated at effective dosages. Romidepsin, like other potent HDAC inhibitors, is frequently active in cell line models, and also because of this class's pleotropic effects we did not chose to further explore this compound. However, because romidepsin continues to demonstrate activity and synergistic potentiation of INK128 at concentrations that are <0.1% of Cmax, we believe that further work should be done to investigate this potential relationship.

With regard to combinations that inhibit the RAS pathway, we focused in on pan-PI3K inhibition with BKM120, MEK inhibition with cobimetinib or trametinib, and dual mTORC1/2 inhibition with INK128. We initially hypothesized that the RAS pathway in MPNSTs was balanced between the flux of PI3K and MEK. We were surprised to see that combinations of PI3K+MEK inhibitors demonstrated, at best, additive effects (Figure 2A). Indeed, the strongest synergism was identified in combinations of INK128 with cobimetinib or trametinib. The activity and mechanisms of action represented by these small molecules are consistent with effects seen in previous studies both *in vitro* and in animal models, particularly with regard to observed synergism in combinations that target mTOR or MEK, though prior literature is limited regarding mechanistic effects of this dual inhibition [14, 24, 27–29].

As suggested by the relative response differences between cell lines, for many of the two-drug combinations, there were striking sensitivity differences between the cell lines. Indeed, with few exceptions, we were able to reach FAs between 0.8 and 1.0 in 2 out of 4 cell lines at concentrations well below Cmax. However, the FA values for the other two cell lines reached values of at most 0.6, with relatively minimal treatment effects observed with some combinations (Supplementary Table 3). The relative sensitivity observed for single agents (Figure 1A) is conserved in the combinations – without exception, SNF10.1 and SNF96.2 were more impacted by each treatment than SNF02.2 and SNF94.3 (Figure 2C).

### The genetic status of NF1 is associated with MPNST response to small molecules

**Figure 3:**
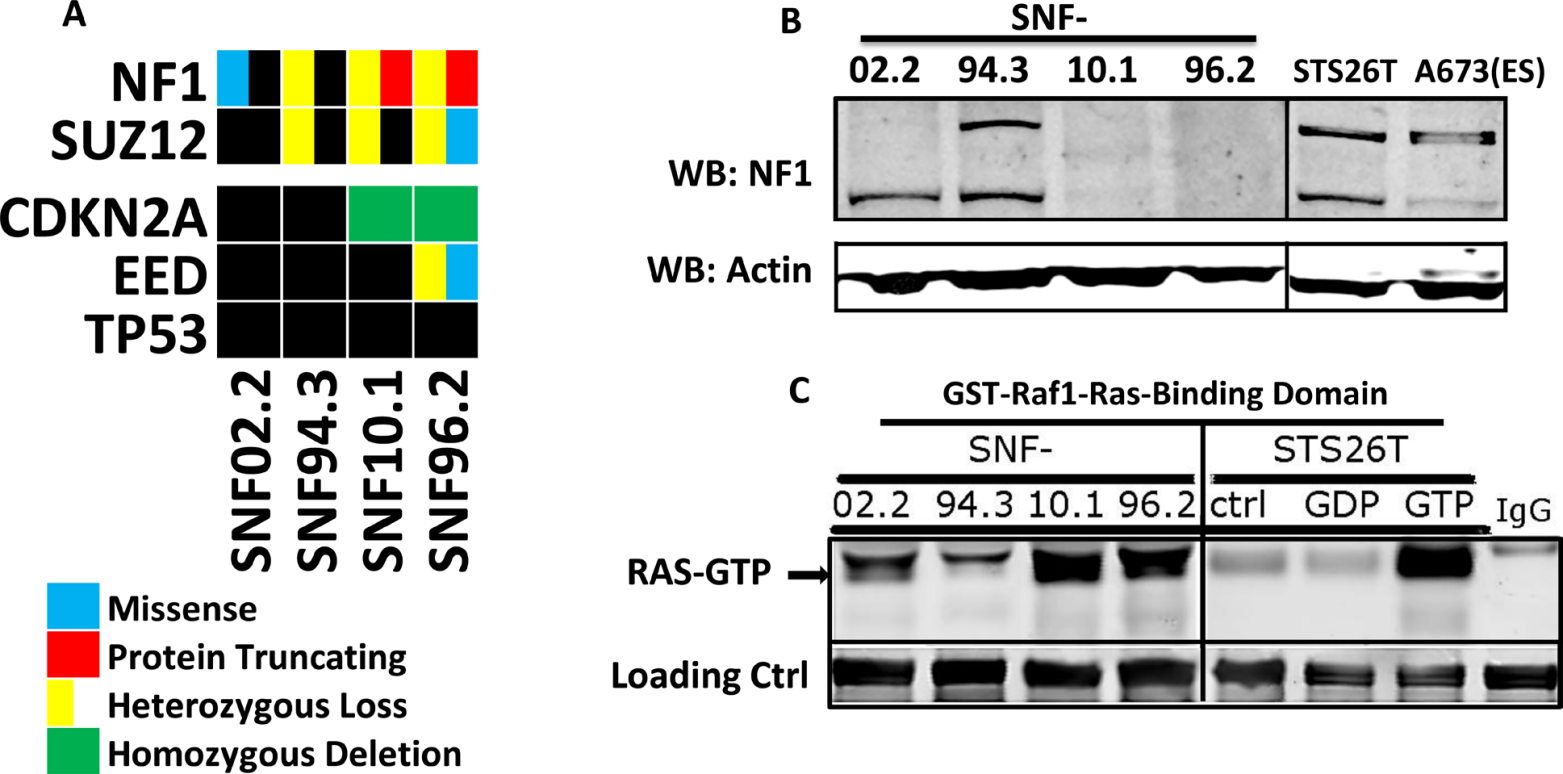
Genetics and biochemical activity of NF1 and RAS in MPNST cell lines (**A**) Oncoprint of genetic background of 5 MPNST cell line models revealed by whole-exome sequencing and CNV analysis (*no CNV data for STS26T). Black box indicates no mutation found. (**B**) Western blots of neurofibromin in 5 MPNST cell lines and the Ewing Sarcoma line A673. (**C**) Western blot of total RAS following pull-down of GST-Raf1-Ras binding domain, which only interacts with GTP-bound (active) RAS. As a positive control, high concentration GTP treatment of STS26T lysate was used to activate RAS *in vitro*. GDP treatment, which leads to the opposite, provided as negative control.

With the goal of identifying a biomarker for the observed drug sensitivity differences within our cell lines, we performed whole exome sequencing and single nucleotide polymorphism (SNP) arrays of each of the tested cell lines. We additionally performed genomic characterization of the sporadic MPNST cell line STS26T which was utilized as a comparator in later functional experiments. We found mutation of *NF1* in all cell lines except STS26T, confirming those already reported for SNF94.3 and SNF96.2 (Figure 3A). Specifically, SNF02.2 has a missense mutation D1623V for transcript variant 1 NM_001042492 (also known as D1644V depending on the transcript reported) with no detectable second mutation; SNF94.3 has a microdeletion spanning the entire *NF1* locus plus flanking loci (including *SUZ12*), but again no additional *NF1* mutation was found; SNF10.1 has a R1276X nonsense mutation and hemizygous deletion of the other allele; and SNF96.2 has two null alleles (1-bp frameshift mutation plus loss of heterozygosity). Heterozygous copy number loss of *SUZ12* was identified in SNF10.1, SNF94.3, and STS26T. A missense variant in *SUZ12* with loss of heterozygosity was identified in SNF96.2. Homozygous deletion of *CDKN2A* was detected in SNF10.1 and SNF96.2. Copy neutral LOH was present in much of the genome in SNF96.2 including chromosome 17 which contains the *TP53* transcript. Overall, these mutations are analogous to the lesions also observed in sequencing data obtained from MPNST patient tumor samples [30, 31]. Importantly, the genetic feature most pertinent to MPNSTs, the varying status of *NF1* between cell lines, is delineated along the two groups defined by the observed phenotypic differences. The lines with homozygous loss of *NF1* (SNF10.1 and SNF96.2), were significantly more sensitive to chemotherapeutic and RAS targeted treatments than those without complete *NF1* loss, SNF02.2 and SNF94.3.

### RAS activation is inversely proportional to the presence of NF1

To assess the impact of the different *NF1* genotypes at the protein level, we performed Western blots with lysates from the MPNST cell lines. At approximately 240 kD, the predicted MW of neurofibromin, we observed two distinct bands for SNF02.2, SNF94.3 and STS26T and no bands for SNF10.1 and SNF96.2. This is in accordance with the latter cell lines having homozygous loss of the *NF1* gene and is consistent with that previously observed in other labs (Figure 3B) [32–34]. The appearance of two bands in cells with remaining neurofibromin likely corresponds to the existence of two isoforms obtained via alternative splicing, type I and type II. Of the cell lines showing neurofibromin expression, the lower MW type I isoform trends toward predominance. As no additional mutations were found in *NF1*, we can infer that the neurofibromin evident by Western blot has normal function. However, we have not ruled out the contribution of posttranslational modifications to the apparent molecular weight shift [35–37].

To further explore the impact of the observed *NF1* variation on RAS activity, we performed a pull-down of GST-tagged Raf1-Ras-Binding Domain (Raf1-RBD). The *in vitro* Raf1-RBD binding affinity for the active RAS-GTP is 20 nM. Thus, an Anti-RAS blot of the resulting pull-down allows active RAS to be visualized with high specificity. Upon treatment of STS26T lysates with saturating amounts of GTP, we observed a remarkable increase in active RAS-GTP compared to GDP treated or untreated STS26T lysates. Bands similar to the positive control appear in SNF10.1 and SNF96.2, while considerably lower signal is observed in SNF02.2 and SNF94.3 demonstrating that the residual *NF1* levels lead to an increase in RAS activation in these cell line models. This is consistent with the dogma that neurofibromin has a large role in meditating RAS activity in Schwann lineage cells (Figure 3C).

### Small molecule inhibition of factors in the RAS/PI3K/MEK/mTOR pathway correlates with the status of NF1 in MPNST

We hypothesized that the varying levels of neurofibromin/RAS-GTP activity in each cell line may play a role in the observed drug sensitivity differences specifically with regard to MEK activation. We sought to obtain a steady-state view of the RAS-activated phosphorylation cascade for the sake of making comparisons between cell lines in response to different drug treatments. We employed phospho-specific antibody arrays that allow concurrent quantification of 16 phosphorylation sites in the RAS pathway (Supplementary Figure 2). Though we identified many promising two-drug combinations with activity, we focused on agents that target three pivotal nodes – the PI3K inhibitor BKM-120, the MEK inhibitor trametinib, and the dual mTORC inhibitor INK128 (MLN0128, TAK-228). These agents were tested both alone and in a series of two-drug combinations. To determine if agent inhibition of the pathway would affect phosphorylation signaling differently, we selected one hemizygous and one homozygous affected *NF1* cell line, SNF94.3 and SNF96.2, respectively.

**Figure 4:**
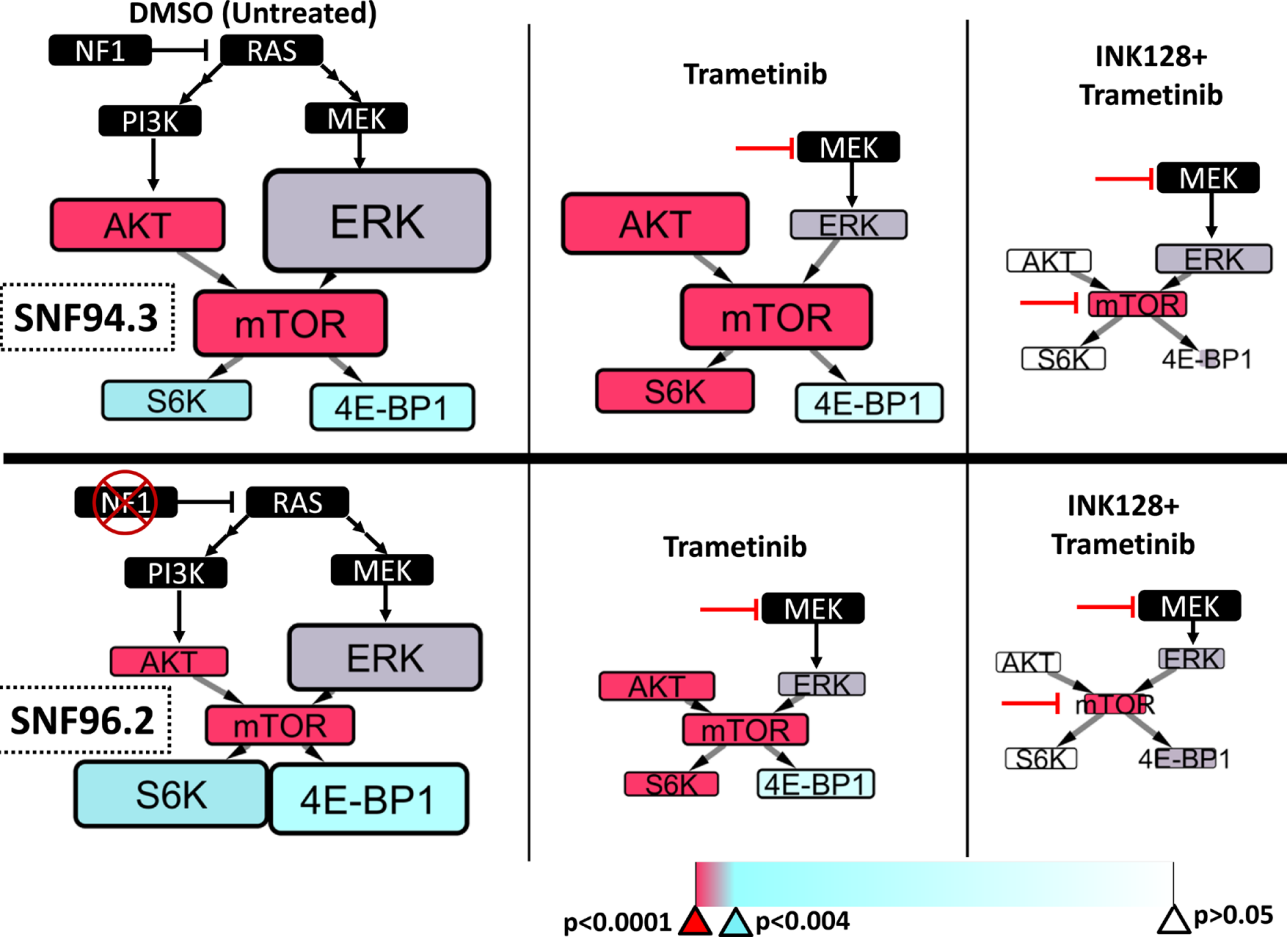
RAS pathway signaling differences between MPNST cell lines The phosphorylation of selected factors downstream of RAS was quantified in SNF94.3 (**top**) and SNF96.2 (**bottom**) following treatment with putative inhibitors of targets downstream using an antibody array. The node size and font size denoting a given factor in a simplified schematic of the PI3K/MEK/mTOR axis are directly proportional to the measured relative fluorescent signal, which is proportional to the amount of phosphorylation for a given target. Due to differences in antibody affinities, relative phosphorylation signal differences can only be remarked upon in contrasting the same target between treatment conditions. The specific treatment (or DMSO control) associated with each schematic is listed on the bottom, and inhibitor lines point to the primary factor targeted. The color of each box represents the statistical significance for each given factor, suggesting that the phosphorylation levels are significantly different between the two cell lines. The associated *p*-values are provided on the color bar. Broadly, red indicates very strong significance (*p <* 0.00001), purple indicates significance (*p <* 0.002), cyan indicates borderline significance (*p <* 0.004), and white indicates low confidence in significance (*p >* 0.05).

Broadly, the phosphorylation antibody array consistently showed higher phosphorylation of factors in SNF94.3 than those in SNF96.2. Two notable distinctions to this overall pattern were the baseline values of the phosphosites 4E-BP1(Thr37/46) and p70 S6 Kinase(Thr421/Ser424) (S6K) which were higher in SNF96.2 than SNF94.3 with *p*-values of 0.0164 and 0.0037, respectively (Figure 4, Supplementary Figure 3). As expected, targeting PI3K, MEK, or mTOR resulted in a decrease in AKT, ERK, or mTOR phosphorylation, respectively (Supplementary Figure 3). However, the most provocative factor measured was PRAS40, a highly regulated target that putatively provides an additional bridge between AKT and mTOR. The relative phosphorylation signal of PRAS40 was consistently higher than any other phosphosite measured in our analysis, regardless of cell line or drug treatment. This extreme PRAS40 signal is not observed in other cell types (e.g. MCF7 cells) [38]. Notably, the overall signaling cascades in SNF94.3 were considerably more resistant to perturbation by the tested agents than SNF96.2, in effect corroborating the sensitivity differences observed in the cell viability assays.

We achieved effective suppression of pathway signaling relative to control following the treatment with the use of two-drug combinations. A striking difference between the two cell lines revealed itself under those conditions. While the signaling cascades in SNF96.2 could be equally reduced when combining BKM-120+INK128, BKM-120+trametinib, or INK128+trametinib, SNF94.3 resisted profound changes to pathway flux in all cases aside from INK128+trametinib (Figure 4, Supplementary Figure 3). Interestingly, this corresponds well with our cell viability data where we observed that of these combinations, INK128+trametinib demonstrated the strongest synergism (Figure 2). We focused our analysis on the INK128+trametinib combination and endeavored to elucidate the statistical relationship between SNF94.3 and SNF96.2 in response to these agents. For each of the five antibody targets in our final investigation, the most parsimonious ANOVA model was obtained (Supplementary Statistics).

Overall, we found that the mean phosphorylation signals for SNF 94.3 were higher than those for SNF 96.2 with some exceptions (Figure 4, Supplementary Figure 2). As alluded to above, phosphorylation of 4E-BP1 (Thr37/46) in response to DMSO and INK128+trametinib treatment was relatively higher in SNF96.2 than SNF94.3 (Figure 4, bottom vs top) with statistical significance (*p* = 0.0164 and 0.0026, respectively). Similarly, S6K (Thr421/Ser424) had significantly greater phosphorylation signal at baseline in SNF96.2 (*p* = 0.0037). S6K in SNF96.2 was far more sensitive to trametinib than in SNF94.3 (*p <* 0.0001). Phosphorylation of Akt (Ser473) was considerably higher in SNF94.3 at baseline compared to SNF96.2 (*p* = 0.0001). Interestingly, in response to trametinib treatment, phosphorylation of AKT was increased relative to DMSO in SNF94.3; this response was significant compared to SNF96.2 where no increase in AKT was observed (*p <* 0.0001). This apparent compensatory response to reduction in MEK signaling may play a role in the overall tolerance to perturbation observed in SNF94.3. Indeed, even though phosphorylation of Erk1/2 (Thr202/Tyr204) was reduced in response to trametinib for both SNF94.3 and SNF96.2, the other factors in SNF94.3 were essentially unchanged compared to control. This is not the case for SNF96.2 where a decrease in phosphorylation was detected in the downstream factors. Phosphorylation signal of mTOR (Ser2481) was significantly higher in SNF94.3 overall regardless of treatment (*p* < .0001). INK128 was effective in reducing mTOR phosphorylation in both cell lines relative to control. It's notable that AKT and ERK were also substantially impacted by inhibition of mTOR in both cell lines, though ERK was impacted to a slightly greater degree in SNF96.2 when compared to SNF94.3. The pathway is essentially shut down with treatment of both INK128 and trametinib when comparing phosphorylation signals to baseline. While the absolute difference seems relatively minor, the percentage difference between INK128 and INK128+trametinib is considerable, particularly when considering the phosphorylation levels at baseline.

### MPNST sensitivity to MEK and mTOR inhibition is proportional to neurofibromin abundance

Having established an association between the relative level of residual full-length neurofibromin and the difference in S6K signaling downstream of RAS we wanted to determine the degree of causality via siRNA targeting of *NF1*. Following transfection of SNF02.2, SNF94.3, and STS26T with an siRNA construct mapped to the *NF1* gene, we were able to observe a notable reduction in the neurofibromin band relative to siRNA control construct as detected on Western blot. This indicates that *NF1* expression is indeed reduced in response to siRNA transfection.

**Figure 5:**
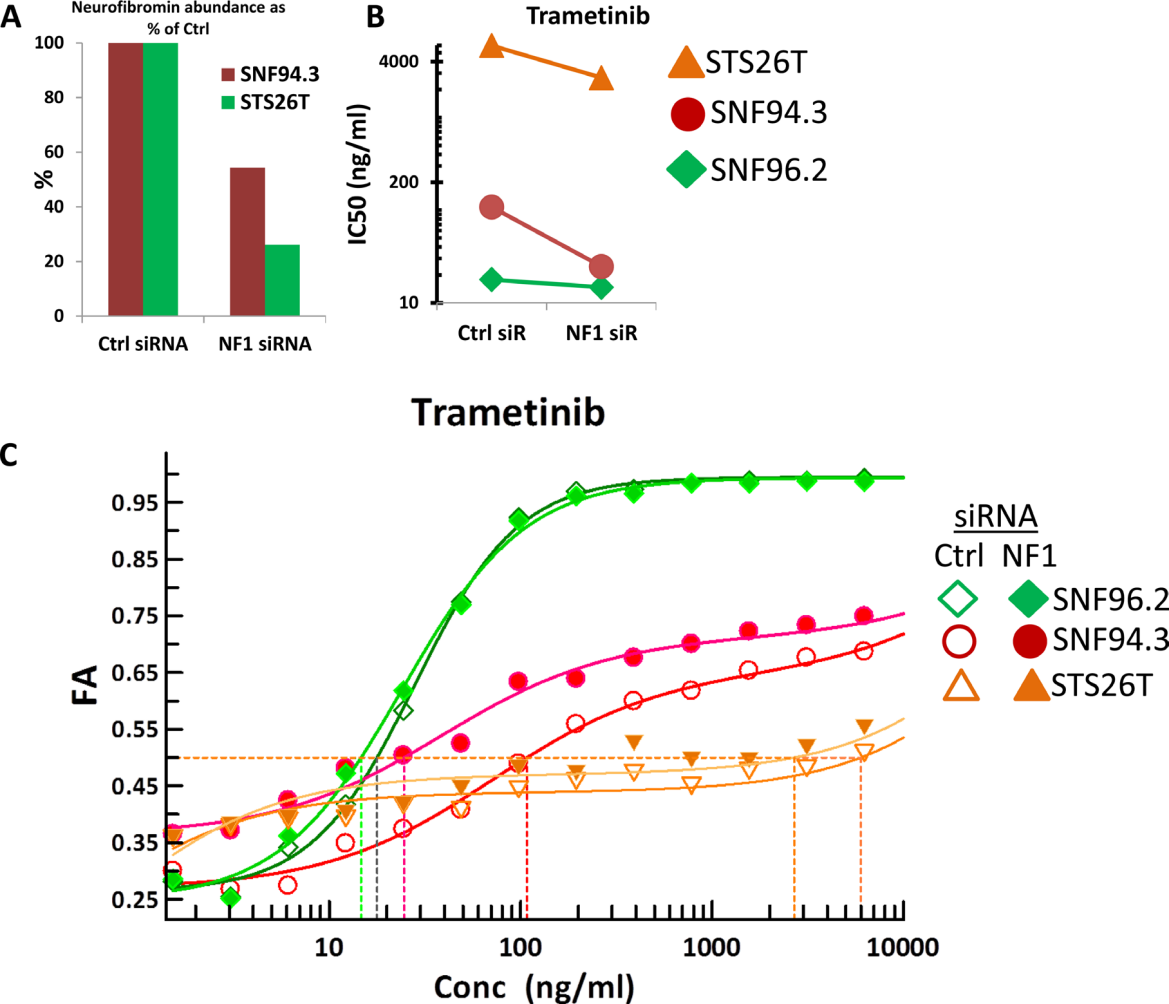
Drug sensitivity alterations in response to NF1 knockdown (**A**) Relative abundance of neurofibromin protein in response to control siRNA and *NF1* siRNA in SNF94.3 and STS26T, normalized to α-actinin. (**B**) Shift in IC50 for indicated drug treatment in given cell lines in response to *NF1* RNAi compared to control siRNA. (**C**) Full dose response curves for trametinib in SNF94.3, SNF96.2, and STS26T. Ctrl siRNA and *NF1* siRNA are represented by unfilled and filled shapes, respectively.

Following confirmation that the siRNA could cause knockdown, SNF94.3, SNF96.2, and STS26T were subjected to transfection with siRNA. After cells were returned to serum-containing media and briefly allowed to equilibrate, dose response curves were generated for trametinib, INK128, and BKM-120. Because SNF96.2 already had loss of both *NF1* alleles, further knockdown of this gene would not be expected to cause further impact on the cell line's phenotype. As anticipated, depletion of *NF1* in SNF94.3 and STS26T resulted in a considerable decrease in IC50s relative to control siRNA treatment (Figure 5A, 5B). *NF1* RNAi had no effect on the response to trametinib by SNF96.2 (Figure 5B). However, the dose response curves for both SNF94.3 and STS26T demonstrated a leftward shift when subjected to *NF1* siRNA (Figure 5C). As depletion of *NF1* in cell lines with residual neurofibromin results in greater sensitivity to tested agents, partially mimicking the phenotype of the *NF1*-null line SNF96.2, this suggests that the relatively greater drug sensitivity phenotype observed in *NF1*-/- cells may be partly caused by the *NF1* status in these lines.

## DISCUSSION

Despite concerted study, we continue to lack effective agents in the clinic for patients with advanced or metastatic MPNST [39]. In addition to the clinical needs, MPNST remains a good model for targeting RAS activated malignancies, the most common oncogenic transformation in human cancers [40]. While targeting RAS has proven elusive, we believed a combination strategy with clinically investigated compounds would be the most immediately translatable. Importantly, all screens performed in this effort were at concentrations achievable in human serum based on completed clinical trials. In addition to the novelty of high-throughput drug combination screening against multiple MPNST models, this study is also the most comprehensive to explore the genotype-phenotype association between *NF1* loss, the primary oncogenic driver of MPNST, and the quantifiable response to relevant chemotherapeutic agents.

Our findings in the context of single agent activity against MPNST cell lines both corroborate and expand upon what has already been published. Where our work overlaps with another recent single agent screening finding activity of the proteasome inhibitor bortezomib; MEK inhibitors cobimetinib, selumetinib, and trametinib (compared with a relative lack of activity for binimetinib); the PI3K inhibitor BKM120; the microtubule inhibitor docetaxel; the HDAC inhibitor panobinostat; and the CDK inhibitor palbociclib there is little deviation [23]. Their primary conclusions regarding MEK sensitivity are also corroborated by this work. We further expand on these findings by assessing the most promising agents in combination at concentrations well below published Cmax values in order to evaluate two-drug activity and synergy in MPNSTs.

We found that two-drug combinations can significantly dampen phosphorylation downstream of RAS and effectively reduce cell viability, but single agents were not nearly as effective in diminishing downstream pathway phosphorylation or cell proliferation to any considerable degree. Amongst the active agents are INK128 (Sapanisertib), a dual mTORC1/2 inhibitor which is currently being explored in a phase II trial (NCT02601209) with an MPNST cohort and with additional preclinical data [41]. INK128 in combination with a MEK inhibitor, either cobimetinib or trametinib, was found to have remarkably strong synergism at a broad array of concentrations, including the lowest tested concentrations, which are well below physiological maximums. This may prove to be indispensable in the future development of therapeutic treatments utilizing these agents, as such a synergistic relationship would potentially allow for clinically relevant efficacy at relatively low concentrations. This could be essential for agents whose mechanisms commonly result in adverse events. As two-drug regimens including inhibition of MEK or mTOR have demonstrated significant toxicities, assay development including optimal order of addition, peak levels and durations needed for effectively targeting these pathways in cancer cells will likely be needed to optimize the therapeutic index for patients. Our characterization of INK128 in combination with trametinib or cobimetinib in multiple cell line models of MPNST suggests a clinical trial that specifically aims to investigate this agent in patients with *NF1*-associated tumors may prove a promising avenue for investigation.

The drug synergism we have observed when combining MEK inhibitors with other drugs in addition to INK128 (Supplementary Table 3) lends further support to the burgeoning model that suggests exposing both plexiform neurofibromas and MPNST cells to MEK inhibition sensitizes them to other strategies that might not demonstrate noticeable activity alone. Several recent studies have characterized individual therapies that contain a MEK inhibitor along with other interventions such as nanoparticle-based photothermal therapy [42], all*trans* retinoic acid (ATRA) [43], BMP-2 inhibitors [44], the BRD4 inhibitor JQ1 [45], inhibition of MAPK-interacting kinases (MNKs) with cabozantinib [46], and other targets that lie downstream of RAS [15]. Altogether, these findings suggest that MEK-targeting strategies will be indispensable in the development of combinatorial therapies for MPNST and other NF1-associated diseases. The current study not only supports this model but also opens up new possibilities for exploration of the mechanisms underlying the variable sensitivity of MPNSTs to drugs that target MEK and associated factors.

In addition to MEK inhibition, combinations of agents that contained the dual mTORC1/2 inhibitor INK128 demonstrated considerable synergism. The likely importance of mTOR in our MPNST models is bolstered by the remarkably strong signal we observed for the mTOR repressor PRAS40 when assaying the phosphorylation status of an array of factors in the RAS/AKT/MEK/mTOR axis. Recent advances in understanding PRAS40 suggest this factor plays a significant role in regulation of mTOR in normal cellular homeostasis as well as disease contexts. A newly resolved crystal structure of Arabidopsis mTORC1 shows direct binding of PRAS40 to mTOR at multiple residues including the rapamycin-binding pocket [47]. Abnormal PRAS40 activation and high levels of phospho-PRAS40 have been indicated as putative biomarkers in melanoma, prostate cancer, and NSCLC [48], but the role of PRAS40 in MPNST or neurofibromatosis has yet to be explored. Hyperphosphorylation of PRAS40 may be a major conduit through which the RAS signal is able to reach mTOR in the context of dysfunctional neurofibromin. Interestingly, PRAS40 has also been implicated in the regulation of TP53 by way of the E3 ubiquitin ligase MDM2, suggesting that PRAS40 may have a role in tying in both of these pathways that are critical to so many sarcomas.

Given additional oncogenic mutations outside of the RAS/MEK/mTOR axis, it may still prove necessary to combine agents that target pathways parallel or perpendicular to the RAS signaling cascade for truly effective clinical outcomes. Thus, this relatively homogenous malignancy may need 3+ agents to address its pathophysiology. As the importance of PRC2 subunits SUZ12 and EED are further revealed in the context of MPNST, treatment strategies that include targeting of epigenetic regulatory factors may be of interest. We included the Class I/II HDAC inhibitor romidepsin in our screens and observed fairly strong effects, particularly with regard to synergism when combined with INK128. This provides some credence to one model of MPNST oncogenesis, which primarily involves a combination of RAS deregulation and epigenetic dysregulation, due to lesions in *NF1* and PRC2, respectively. Of course, due to the multitude of targets affected by HDAC inhibitors, more specific studies are required to realize this mechanism [49].

We extensively investigated mechanisms underlying drug sensitivity at both the genetic and the protein level. Importantly, *NF1* status, in terms of levels of function, appears to mediate the optimal downstream pathway targets with MEK inhibition being notably important in the case of *NF1* heterozygous loss. Relatedly, we expected that due to the putative parallelism of MEK and PI3K in the canonical RAS pathway, PI3K inhibition and MEK inhibition might be interchangeable as long as a related factor, such as mTOR, was being sufficiently targeted. However, at both the level of drug synergism and RAS pathway modulation, PI3K inhibition was insufficient in providing notable effects in cells with heterozygous *NF1* loss. Throughout this study, we observed a series of correlations between response and the observed state of *NF1* and the proportional impact on RAS activation. We did not directly rule out the possibility that CDKN2A, which also differed in status between cell lines, may also play a role in these sensitivity differences. However, it has been previously reported that mutations in CDKN2A (among others) plays a role in resistance to paclitaxel [50]. We do not see a significant difference in the activity of the analogous drug docetaxel in the context of CDKN2A mutation. Moreover, the central role of NF1 in regards to drug sensitivity differences was supported by siRNA knockdown experiments. Indeed, we observed that, particularly in the case of the MEK inhibitor trametinib, knockdown of *NF1* in cell lines either wildtype for or with het-loss of this gene resulted in a shift in the sensitivity phenotype toward that of the tumor cells with complete ablation. However, due to the incomplete knockdown of *NF1* with RNAi, more complex molecular tools may be essential for further confirmation of this causal relationship. Nonetheless, we hypothesize that residual *NF1* plays a more significant role in MPNST drug resistance than other identified factors based on these findings.

The experimental conditions of this study potentially provide the foundation of a model system for assessing the impact of small molecule treatment regimens on two different cell types that may simultaneously exist in MPNST and/or neurofibromatosis patients. The lack of a second *NF1* mutation in SNF02.2 and SNF94.3 is unusual given that both were derived from metastatic sites from high-grade MPNSTs; this challenges the paradigm that *NF1* MPNSTs have both *NF1* alleles mutated. However, it could be that the residual neurofibromin has somewhat reduced GAP activity due to its inclusion of the alternative exon within the GAP domain, failing to meet a high enough level of RAS control [51]. Or, additional genetic or epigenetic changes at other loci could compensate for *NF1* heterozygosity, to allow MPNST development. Such a model may prove useful for the evaluation of novel treatment regimens and provide a glimpse into how systemic chemotherapy may impact non-tumor cells. Relatedly, the importance of MEK targeting observed in this study is particularly timely with the recent identification of single agent MEK inhibition being the most active therapy for plexiform neurofibromas, precursor lesions for MPNST [52]. Selumetinib was included in the current study but provided the least promising activity in the MPNST models we tested. We have also observed that more potent MEK inhibitors such as trametinib have had more than expected toxicity when used off label in plexiform neurofibroma patients perhaps suggesting that there is an optimal level of MEK suppression to allow for antitumor activity without toxicity in MPNST patients that would be lower than the maximally tolerated single agent dose (AB, personal communication). This could facilitate combination therapy. Overall, we believe our data further supports RAS blockade as a strategy for MPNST through combination inhibition of MEK and dual mTOR.

## MATERIALS AND METHODS

### Investigational agents

Agents used included both cytotoxic and targeted agents, most obtained directly from Selleck Chemicals (Houston, TX, USA), Sequoia Research Products (Pangbourne, UK), and Sigma-Aldrich (St. Louis, MO, USA). Agents were assessed at clinically achievable concentrations, which were extrapolated from published human pharmacokinetic data, with a focus on Phase I trials, ideally containing pediatric patients - see Supplementary Table 1 for a full listing, including pharmacokinetic details of all agents used in this study. Stock solutions were typically made for each compound in DMSO, unless solubility properties required an alternative solvent, and were stored at –20°C. Structures for all agents are available in public databases.

### Cell lines and culture conditions

The MPNST cell lines SNF02.2, SNF94.3 and SNF96.2 have been previously published by M. Wallace and are available at ATCC [20, 21]; SNF10.1, gifted to us by M. Wallace (unpublished), is an MPNST cell line more recently established from a recurrent tumor. Sporadic MPNST cell line STS26T (not from an NF1 patient) was gifted from Daniel Scoles at the University of Utah Health Sciences Center. Cells were cultured in DMEM with glutamine, glucose, pyruvate and 10% FBS. Cells were maintained at 37°C and 5% CO2. Cell lines were tested for absence of mycoplasma quarterly using the MycoAlert test kit (Lonza Rockland, Rockland, ME, USA). Cell line identities for SNF02.2, SNF94.3, and SNF96.2 were authenticated, showing a 100% match to known STR profiles in the ATCC database. STR Profiles for SNF10.1 and STS26T have not yet been entered into any of the standard databases. Nevertheless, the lack of significant partial STR matches suggests an absence of any cross-contamination, and growth rate and morphology is consistent with known properties of these lines.

### Cell viability assays

Screening methodology developed previously [53, 54] was used to analyze single agent and two-drug combinations for MPNST. Prior to any assay containing drug treatment, growth characterization profiles for each cell line were determined in 384 well plates with a dilution series of starting cell number. Log-phase growth was observed after 24 hours when plated at several different starting numbers, but 1800 cells per well was found to be optimal for all cell lines in this study. Cells were subjected to treatment for 72 hours. Cell-Titer Glo (Promega, Madison, WI, USA) was added to 384-well plates containing drugged cells with the Precision XS liquid handling station (Bio-Tek Instruments, Winooski, VT, USA). Luminescence was measured after 30 minutes on a shaker at room temperature using a Cytation 3 plate reader (Bio-Tek Instruments). Raw data were transferred to custom-built Microsoft Excel workbooks, where subsequent background subtraction, normalization, and processing were conducted.

### Single-agent screening

Individual agents were screened across at least 4 cell lines. Candidates were characterized at a minimum of 3 concentrations – one near physiological maximum (Cmax), one at 20% this level, and another at 4%. For drugs of interest, full dose response curves were generated, and IC50 values were determined using a suitable sigmoidal equilibrium model regression fit using the XLfit Excel add-in version 5.5 (IDBS, Guildford, Surrey, England).

### Two-drug combination screening

Two-drug combinations were screened in 384-well plates and evaluated at 25 discrete concentration ratios in a 5 × 5 matrix to assess efficacy and synergy, as described previously [53]. Drug synergism was determined using the Chou-Talalay/Combination Index method. Combination indices (CIs) provide a measure of drug synergy where a CI = 1 indicates pure additivity of drug effects, a CI<1 indicates synergistic effects, and a CI>1 indicates drug antagonism. Dose response for each drug was measured in each subsequent experiment to control for batch effects and to mitigate technical artifacts. Single agent dose response was compared with 2-drug combination efficacy to derive a CI value using the median-effect principle. In this method, dose-effect curves for each single agent are generated using the median-effect equation: F_A_/F_U_= (D/D_m_)_m_, where F_A_ and F_U_ = fraction affected and fraction unaffected, respectively (F_U_ = 1–F_A_), D = dose of the drug, D_m_ = dose required for 50% effect (analogous to the IC50), and m = exponent signifying each dose response curve's sigmoidicity. CIs were determined by the isobologram equation for mutually nonexclusive drugs with different modes of action: For x% inhibition, CI = (D)_1_/(Dx)_1_ + (D)_2_/(Dx)_2_ + (D)_1_(D)_2_/(Dx)_1_(Dx)_2_, where (Dx) and (D) represent the single agent concentration and the two-drug combination concentration, respectively, at which Drug1 and Drug2 are isoeffective, inhibiting by x%. All FA and CI calculations were performed in a custom built Microsoft Excel template using the XLfit (IDBS) add-in for fitting linear regressions to dose response data and for calculating Dm and m.

### Genomics

#### Whole-exome sequencing

Sequencing libraries were generated from 1 μg of DNA using the Kapa Library Preparation Kit (Kapa Biosystems, Inc., Wilmington, MA, USA). Library DNA size and quality parameters were evaluated with the Agilent BioAnalzyer (Agilent Technologies, Santa Clara, CA, USA), and equimolar amounts were used for whole-exome enrichment using the Roche NimbleGen SeqCap EZ Exome Library v3.0 kit, which targets 64 Mb of genomic DNA, covering over 20,000 genes (Roche NimbleGen, Inc., Madison, WI, USA). Each library was quantitated with the Kapa Library Quantification Kit and each enriched DNA library was sequenced on an Illumina NextSeq 500 v2 sequencer to generate approximately 100 million 75-base paired-end reads for a final average target coverage depth of 100X. The raw sequence data were demultiplexed using the Illumina bcl2fastq2 software (Illumina, Inc., San Diego, CA, USA).

### Whole-exome sequencing data analysis

Sequence reads were aligned to the reference human genome (hs37d5) with the Burrows-Wheeler Alignment Tool (BWA), and duplicate identification, insertion/deletion realignment, quality score recalibration, and variant identification were performed with the Picard toolkit and Genome Analysis ToolKit (GATK, Broad Institute, Cambridge, MA, USA). Sequence variants were annotated to determine genic context (i.e., non-synonymous, missense, splicing) using ANNOVAR. Additional contextual information was incorporated, including allele frequency in other studies such as 1000 Genomes, the NHLBI Exome Sequence Project, in silico functional impact predictions, and observed impacts from databases like ClinVar (http://www.ncbi.nlm.nih.gov/clinvar/) and the Collection Of Somatic Mutations In Cancer (COSMIC) [55–57].

### Copy Number Variation (CNV) Analysis

Copy number variation (CNV) and loss-of-heterozygosity (LOH) status were obtained with the CytoScan HD Assay (Affymetrix, Santa Clara, CA, USA), which was performed on each cell line starting with 250 ng of DNA. The CytoScan HD assay uses 750,000 SNP probes and 1.9 million non-polymorphic probes to report genome-wide copy number aberrations at a resolution of 25-50 Kb. In addition, the assay reports genome-wide LOH, including copy-neutral LOH when applicable. The data generated from the assay were normalized, copy number status calculated, and the data reviewed for quality using the Chromosome Analysis Suite (ChAS) v3.0 (Affymetrix).

### Western blots

Western blots were performed using Novex and NuPAGE SDS-PAGE gel systems (Invitrogen, ThermoFisher, Waltham, MA, USA). NuPAGE 3-8% Tris-Acetate gels were used to resolve the 240kD neurofibromin. RAS was visualized on Novex 4-12% Tris-Glycine gels. Proteins were transferred from gels to nitrocellulose membranes using a wet transfer system. Membranes were blocked and antibodies were diluted using Odyssey^®^ Blocking Buffer (TBS) (LI-COR, Lincoln, Nebraska, USA).

Primary antibodies – Neurofibromin Antibody (D) (sc-67, rabbit polyclonal to a C-terminal epitope; discontinued product), Neurofibromin Antibody (H-12) (sc-376886, mouse monoclonal to AA241-540 near N-terminus), and Neurofibromin Antibody (McNFn27a) (sc-20017, mouse monoclonal made to N-terminal peptide) - were used to detect neurofibromin at a 1:200 dilution (Santa Cruz Biotechnology, Inc., Dallas, Texas, USA). Primary antibodies - β-Actin D6A8 (#8457, rabbit monoclonal to N-terminal peptide) (Cell Signaling Technology, Danvers, MA, USA) and α-actinin Antibody (H-2) Alexa Fluor^®^ 647 (sc-17829 AF647, mouse monoclonal to AA593-892) (Santa Cruz Biotechnology) - were used as loading controls. Secondary antibodies – to rabbit IgG (H&L, IRDye^®^ 800 Conjugated; discontinued product) (Rockland, Limerick, PA, USA), and mouse IgG (H&L, IRDye^®^ 700 Conjugated) (Rockland) allowed fluorescent visualization with the Odyssey^®^ Imaging System (LI-COR). Western blot bands were quantified using Image Studio Lite Ver 5.2 (LI-COR).

### Antibody array

Antibody array experiments were performed using the PathScan^®^ Akt Signaling Antibody Array Kit (#9474, Chemiluminescent Readout) (Cell Signaling Technology). Array slides were visualized using the Odyssey^®^ Imaging System (LI-COR) and signals were quantified using Image Studio Lite Ver 5.2 (LI-COR). Subsequent data normalization was conducted in a custom-built Microsoft Excel template. Pathway diagrams were generated using Cytoscape v 3.2.1 (San Diego, CA, USA).

### Statistical analysis

#### Antibody array

For each phosphosite, relative fluorescent signal was normalized by division of the average of three positive control values. A further normalization was conducted between the two antibody array experiments by subtracting the mean and dividing by the standard deviation of all expression values of each antibody within each experiment in order to make the antibody expression values consistent and comparable between the two experiments for subsequent analysis. We next fitted an ANOVA model for each antibody expression with three covariates: drug treatment, cell line, and experiment. Backward elimination was used to obtain the most parsimonious ANOVA model. Statistical comparison between cell lines SNF94.3 and SNF96.2 was made by examining significance of each contrast test of the two cell lines based on the final ANOVA model of each antibody target. Bonferroni correction was used to correct for multiple comparisons from the combinations among four different treatments and five antibodies we closely examined for our study. That is, statistical significance level ≤0.0025 was used for inferring the cases with a significant expression difference between the two cell lines for each combination of drug treatment and antibody. Statistical analysis was conducted with SAS 9.4 (Cary, NC, USA).

### Drug sensitivity comparison

Relative response was determined by normalizing fraction affected (FA) values against LOG(concentration). Statistical comparison between drug relative response values for cell lines SNF02.2, SNF94.3, SNF10.1, and SNF96.2 was made through application of the Mann–Whitney–Wilcoxon test (two-tailed). The z-statistic was obtained and *p*-value determined, which allowed inference of significant sensitivity differences between cell lines.

### RNAi Transfection

Cells were grown under serum starved conditions in modified EMEM with minimal supplements (siRNA Transfection Medium, sc-36868), and siRNA constructs were introduced using a polycationic lipid-based transfection reagent (siRNA Transfection Reagent, sc-36868) which were both obtained from Santa Cruz Biotechnology, Dallas, Texas, USA. Neurofibromin was targeted with neurofibromin siRNA (h) (sc-36036, pool of 3 proprietary NF1-specific constructs). As a control, cells were treated with Control siRNA (FITC Conjugate)-A (sc-36869, scrambled proprietary sequence with no known targets). All reagents were obtained from Santa Cruz Biotechnology.

## SUPPLEMENTARY MATERIALS FIGURES AND TABLES








