# Supplementary material for: Neurofibromin level directs RAS pathway signaling and mediates sensitivity to targeted agents in malignant peripheral nerve sheath tumors
Source: Oncotarget. 2018 Apr 27;9(32):22571–85. doi: 10.18632/oncotarget.25181 (PMC5978249; doi:10.18632/oncotarget.25181)
Supplement: Supplementary file 1 [file oncotarget-09-22571-s001.pdf]

# Neurofibromin level directs RAS pathway signaling and mediates sensitivity to targeted agents in malignant peripheral nerve sheath tumors

## SUPPLEMENTARY MATERIALS

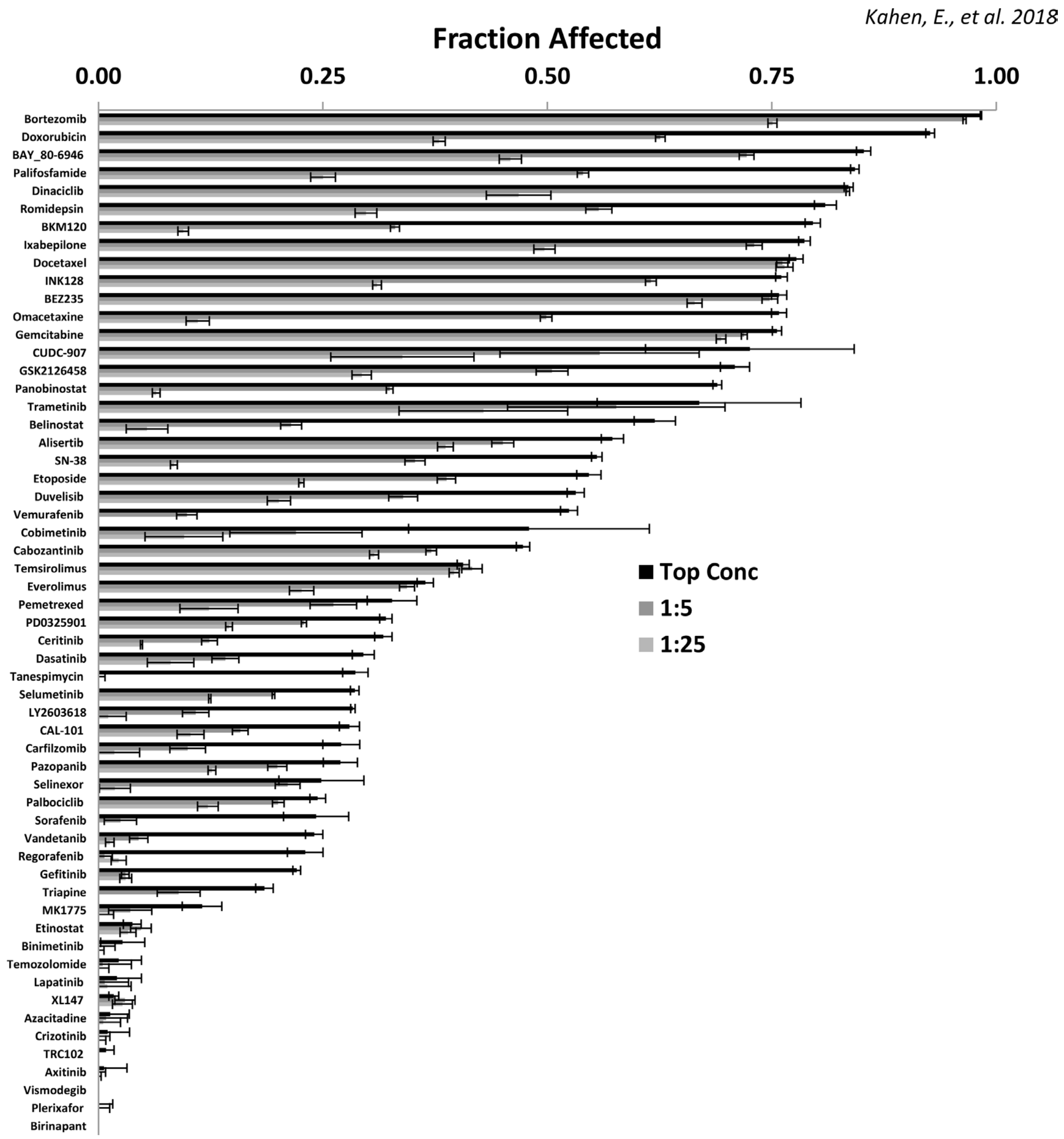


**Supplementary Figure 1: Average fraction affected (FA) values across 4 MPNST cell lines when treated with a given drug at Cmax, 1/5 Cmax, and 1/25 Cmax.**

**A**

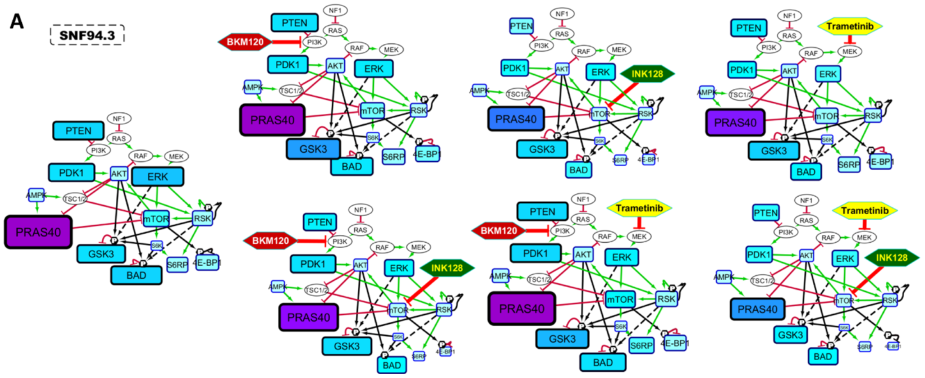


*Kahen, E., et al. 2018*

**B**

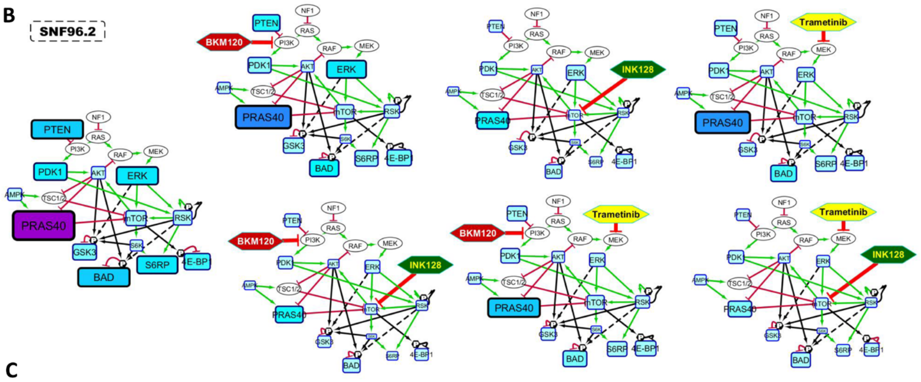


**C**

| Phosphosite Legend | | | |
|---|---|---|---|
| **Target** | **Phosphosite** | **Phosphorylated By*** | **Putative Biology*** |
| **4E-BP1** | **Thr37/46** | **mTORC1** | Inhibitory phosphorylation by mTOR |
| **AKT** | **Thr308** | **PDK1** | Activation Loop phosphosite |
| **AKT** | **Ser473** | **mTORC2** | AKT regulated by mTORC2 via Ser473 |
| **AMPKα** | **Thr172** | **LKB1, CAMKK2** | Inhibits mTOR through multiple mechanisms |
| **BAD** | **Ser112** | **RSK1, AKT, S6K** | Inhibitory phosphorylation induced by multiple possible upstream factors |
| **ERK1/2** | **Thr202/Tyr204** | **MEK1** | MAPK (MEK) cascade |
| **GSK-3α** | **Ser21** | **AKT, ERK, RSK, S6K** | Inhibitory phosphorylation induced by multiple possible upstream factors |
| **GSK-3β** | **Ser9** | **AKT, ERK, RSK, S6K** | Inhibitory phosphorylation induced by multiple possible upstream factors |
| **mTOR** | **Ser2481** | **mTOR (autophosphorylation)** | mTOR autophosphorylation follows Ser2488 phosphorylation |
| **p70 S6 Kinase (S6K)** | **Thr389** | **S6K (autophosphorylation)** | Downstream of mTOR (Thr389 *possibly parallel* to RAS signaling). Downstream of growth factors |
| **p70 S6 Kinase (S6K)** | **Thr421/Ser424** | **S6K (autophosphorylation)** | Required for full activation |
| **PDK1** | **Ser241** | **PDK1 (autophosphorylation)** | Autophopshorylation induced by growth factors |
| **PRAS40** | **Thr246** | **AKT** | Inhibitory phosphorylation by AKT relieves inhibition of mTOR |
| **PTEN** | **Ser380** | **LKB1 / PLK1** | Negative regulator of PI3K/AKT; MEK1 involved in membrane recruitment |
| **RSK1** | **Thr421/Ser424** | **RSK1 (autophosphorylation)** | Autophosphorylation; downstream of ERK1/2, PDK1, |
| **S6 Ribosomal Protein (S6RP)** | **Ser235/236** | **S6K** | Activated by mTOR |

*limited to upstream factors relevant to current study; not intended to be exhaustive.

**Supplementary Figure 2:** Pathway schematics showing all factors assessed via phospho-antibody array in (**A**) SNF94.3 and (**B**) SNF96.2. (**C**) Legend for all phosphosites measured in array.

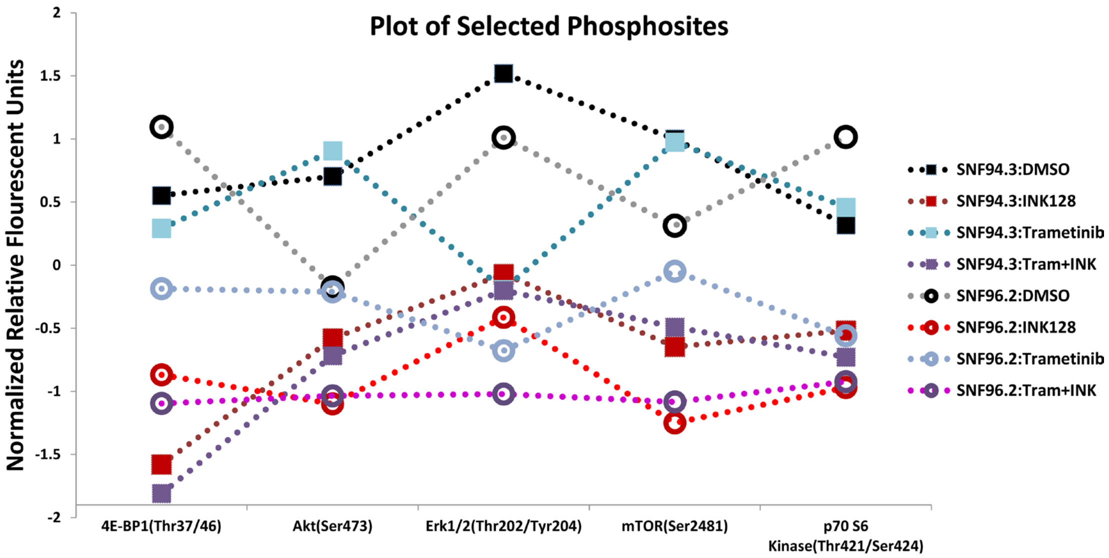


**Supplementary Figure 3: Line plot of the data presented in Figure 4.**

**Supplementary Table 1: Drug information sheet and associated references.** See Supplementary_Table 1

**Supplementary Table 2: Full table of FA values from single agent screen.** See Supplementary_Table 2

**Supplementary Table 3: Full table of FA and CI values from all drug combinations tested.** See Supplementary_Table 3
