# Supplementary material for: Neurofibromin level directs RAS pathway signaling and mediates sensitivity to targeted agents in malignant peripheral nerve sheath tumors
Source: Oncotarget. 2018 Apr 27;9(32):22571–85. doi: 10.18632/oncotarget.25181 (PMC5978249; doi:10.18632/oncotarget.25181)
Supplement: Supplementary file 2 [file oncotarget-09-22571-s002.docx]

| **Drug** | **Brand Name** | **Mechanism / Target of Inhibition** | **MW**  **(g/mol)** | **t-max (h)** | **t-1/2 (hr)** | **Cmax (ng/ml)** | **AUC (ng*h/ml)** | **Source(s)** |
| --- | --- | --- | --- | --- | --- | --- | --- | --- |
| 5-Azacitidine | Vidaza | Nucleoside analogue/DNA methylation | 244.2 | 0.5 | 0.7 | **2750** | 1044 | Agency, European Medicines 2009 |
| Alisertib (MLN8237) | Alisertib | Aurora kinase inhibitor | 518.9 | 2 | 8 | **3892** | 39023 | Mossé, Lipsitz et al. 2012 |
| Axitinib (AG013736) | Inlyta | VEGF, PDGF | 386.5 | 4 | 3 - 6 | **28** | 265 | Chen, Tortorici et al. 2013 |
| AZD1775 (MK1775) |  | Wee1 inhibitor // CDK1 activator | 500.6 | 3.7 | 11 | **746** | 4821 | Do, Wilsker et al. 2015 |
| BAY 80-6946 (Copanlisib) |  | PI3K (Pan-Class I) | 480.5 | 0.5 | 2 | **312** | 890 | Ramanathan, Hoff et al. 2015 |
| Belinostat (PXD101) | Beleodaq | HDAC (Class I/II/IV) | 318.3 | 0 | 0.7 | **32124** | 9990 | Steele, Plumb et al. 2008 |
| Dactolisib (NVP-BEZ235) |  | PI3K (Pan-class I) & mTORC1/mTORC2 | 469.5 |  | 4 | **500** | 10000 | Burris, Rodon et al. 2010 |
| Binimetinib (MEK162 / ARRY162) | POBID | MEK1/2 inhibitor | 441.2 | 3 | 4 | **493** | 2280 | Bendell, Papadopoulos et al. 2011 |
| Birinapant (TL32711) |  | SMAC mimetic/IAP | 806.9 |  | 25 | **3900** | 4648 | Amaravadi, Schilder et al. 2015 |
| Buparlisib (BKM-120) |  | PI3K (Pan-Class I) | 410.4 | 1 | 40 | **2080** | 22200 | Bendell, Rodon et al. 2012 |
| Bortezomib (PS-341) | Velcade | Proteasome | 384.2 |  | 40 - 193 | **223** | 155 | Janssen-Cilag 2015 |
| Cabozantinib (XL-184) | Cometriq | AXL, FLT3, KIT, MET, RET, TIE-2, TRKB, VEGF1/2/3 | 501.5 |  | 91 | **2310** | 41600 | Kurzrock, Sherman et al. 2011 |
| CAL-101 (GS 1101) | Zydelig | PI3K (Class I d) | 415.4 |  |  | **3300** | 15000 | Flinn, Kahl et al. 2014 |
| Carfilzomib (PR-171) | Kyprolis | Proteosome (Irreversible) | 719.9 | 0 | 0.4 - 0.9 | **5718** | 594 | Papadopoulos, Burris et al. 2013 |
| Ceritinib (LDK378) | Zykadia | ALK inhibitor | 558.1 | 6 | 40 | **800** | 16500 | Shaw , Kim et al. 2014 |
| Crizotinib (PF-2341066) | Xalkori | ALK/MET | 450.3 | 4 | 42 | **630** |  | Mossé, Lim et al. 2013 |
| Cobimetinib (GDC-0973) | Cotellic | MEK inhibitor | 531.3 | 2.4 | 53 | **273** | 4340 | FDA 2016 |
| Dasatinib (BMS-354825) | Spyrcel | SRC-family TKI | 488.0 | 2 | 2 - 4 | **250** | 742 | Aplenc, Blaney et al. 2011 |
| Dinaciclib (SCH727965) |  | CDK1,2,9 | 396.5 | 1 - 2 | 3 | **821** | 2020 | Nemunaitis, Small et al. 2013 |
| Docetaxel (RP 56976) | Taxotere | Microtubule | 807.9 |  | 11 | **3260** | 4810 | Bruno, Hille et al. 1998 |
| Doxorubicin | Adriamycin, Doxil | DNA, topo II | 543.5 |  | 20 | **2108** | 2406 | Greene, Collins et al. 1983 |
| Duvelisib (IPI-145) |  | PI3K d/g | 416.9 |  | 4 | **3225** |  | Infinity 2016 |
| Entinostat (MS-275) |  | HDAC (Class I) | 376.4 | 1 | 5 | **1460** |  | Gore, Rothenberg et al. 2008 |
| Etoposide Phosphate (VP-16) | Etopophos, Toposar | Topoisomerase II | 588.6 |  | 7 | **20000** |  | Kaul, Igwemezie et al. 1995 |
| Everolimus (RAD001) | Zortress/Afinitor/Certican | mTORC1 | 958.2 | 2 | 30 | **11** | 75 | RxList 2016 |
| Gefitinib (ZD 1839) | Iressa | EGFR | 446.9 | 2.3 | 12 | **2210** | 29 | Daw, Furman et al. 2005 |
| Gemcitabine hydrochloride | Gemzar | Nucleoside analogue | 299.7 |  | 10 | **41000** | 1390000 | Reid, Qu et al. 2004 |
| GSK2126458 (Omipalisb (GSK 212)) |  | PI3K (Pan-Class I) & mTORC1/mTORC2 | 505.5 | 4 | 6 | **42** | 474 | Munster, Noll et al. 2012 |
| INK-128 (TAK228) |  | mTORC1/mTORC2 | 309.3 | 1 - 2 | 6 - 8 | **50** |  | Ghobrial, Siegel et al. 2016 |
| Ixabepilone (BMS-247550) | Ixempra | Microtubule | 506.7 |  | 14 | **100** | 335 | Widemann, Goodspeed et al. 2009 |
| Lapatanib Ditosylate | Tykerb | HER2, ErbB1, ErbB2 | 925.5 | 6 - 8 | 3 | **6200** | 55 | Fouladi, Stewart et al. 2010 |
| LY2603618 (Rabusertib) |  | CHK1 | 436.3 |  | 13 - 15 | **4130** | 38300 | Calvo, Chen et al. 2014 |
| Omacetaxine mepesuccinate | Synribo | 80S Ribosome, Protein Synthesis | 545.6 | 0.6 | 11 | **96** | 909 | Levy, Zohar et al. 2006 |
| Palbociclib (PD-0332991) | Ibrance | CDK4/6 inhibitor | 484.0 | 4 - 6 | 27 | **174** | 1395 | Schwartz, LoRusso et al. 2011 |
| Palifosfamide tromethamine | Zymafos | DNA alkylator/crosslinker | 342.2 |  | 10 | **10000** |  |  |
| Panobinostat (LBH589) | Farydak | HDAC (Class I/II/IV) | 349.4 | 1 | 17 | **22** | 139 | Mu, Kuroda et al. 2016 |
| Pazopanib (GW786034B) | Votrient | VEGF1/2/3, PDGFRAB, FGF1/3, Lck, c-Fms-kit | 474.0 | 3.4 | 31 | **40000** | 401000 | Glade Bender, Lee et al. 2013 |
| PD0325901 |  | MEK1/2 inhibitor | 482.2 | 1 - 2 | 6 | **510** | 1410 | LoRusso, Krishnamurthi et al. 2010 |
| Pemetrexed (LY231514) | Alimta | Anti-folate | 427.4 |  | 3 | **421000** | 611000 | Malempati, Nicholson et al. 2007 |
| Plerixafor 8HCl (AMD3100) | Mozobil | SDF1-alpha/CXCR4 | 794.5 | 0.5 | 5 | **926** |  | Stewart, Smith et al. 2009 |
| Regorafenib (BAY 73-4506) | Stivarga | VEGF2, Tie-2 | 500.8 | 4 | 28 - 51 | **3900** | 70400 | RxList 2016 |
| Romidepsin (Depsipeptide) | Istodax | HDAC (Class I/II) | 540.7 | 4 | 3 | **377** | 2414 | Fouladi, Furman et al. 2006 |
| SAR245408 (XL-147, Pilaralisib) |  | PI3K (Class I) | 448.5 | 4 - 8 | 24 - 88 | **95200** | 2060000 | Shapiro, Rodon et al. 2014 |
| Selinexor (KPT-330) |  | CRM1 inhibitor | 443.3 | 4 | 8 | **976** | 5663 | Alexander, Lacayo et al. 2016 |
| Selumetinib (AZD6244) |  | MEK1/2 inhibitor | 457.7 | 1 | 4 - 9 | **1450** | 5782 | Adjei, Cohen et al. 2008, Leijen, Soetekouw et al. 2011 |
| SN-38 (Irinotecan metabolite) |  | Topoisomerase IB | 392.4 |  | 1.6 | **30** | 104 | Ma, Zamboni et al. 2000 |
| Sorafenib (BAY 43-9006) | Nexavar | VEGF2/3. PDGFRB, FLT3, BRAF, RET | 637.0 | 8.3 | 30 | **3670** | 28300 | Brendel, Ludwig et al. 2011 |
| Tanespimycin (17-AAG) | KOS-953 | HSP90 inhibitor | 585.7 |  | 3 - 6 | **8500** | 11714 | Bagatell, Gore et al. 2007 |
| Temozolomide | Temodar | Alkylator | 194.2 | 1 | 2 | **13000** | 46000 | Horton, Thompson et al. 2007 |
| Temsirolimus (CCI-779) | Torisel | mTORC1 | 1030.3 | 1 | 21 | **2800** | 5190 | Spunt, Grupp et al. 2011 |
| Trametinib (GSK1120212) | Mekinist | MEK1/2 inhibitor | 615.4 |  | 127 | **22** | 370 | GlaxoSmithKline 2014 |
| TRC102 (Methoxyamine HCl) |  | Base-Excision-Repair Inhibitor | 83.5 |  | 22 - 27 | **327** | 5812 | Gordon, Rosen et al. 2013 |
| Triapine (3-AP) | Triapine | RnR inhibitor; Fe chelator | 195.2 |  | 1 | **1560** | 254500 | Murren, Modiano et al. 2003 |
| Vandetanib (AZD6474) | Caprelsa | VEGF2, EGF, RET | 475.4 | 8.5 | 300 | **888** | 6468 | Broniscer, Baker et al. 2010 |
| Vemurafenib (PLX4032) | Zelboraf | BRAF(V600E) | 489.9 | 5 | 35 | **52700** | 3127000 | Grippo, Zhang et al. 2014 |
| Vismodegib (GDC-0449) | Erivedge | Hedgehog pathway, PgP | 421.3 | 24 - 37 | 96 | **3707** | 211070 | Gajjar, Stewart et al. 2013 |
|  |  |  |  |  |  |  |  |  |

**Supplementary Table S1: Drug Information**

1. Agency EM. Assessment report for VIDAZA. International Nonproprietary Name: azacitidine. Canary Wharf, London, UK. 2009.

2. Mossé YP, Lipsitz E, Fox E, Teachey DT, Maris JM, Weigel B, Adamson PC, Ingle MA, Ahern CH, Blaney SM. Pediatric phase I trial and pharmacokinetic study of MLN8237, an investigational oral selective small-molecule inhibitor of Aurora kinase A: a Children’s Oncology Group Phase I Consortium study. Clin Cancer Res. 2012; 18:6058–64. <https://doi.org/10.1158/1078-0432.CCR-11-3251>.

3. Chen Y, Tortorici MA, Garrett M, Hee B, Klamerus KJ, Pithavala YK. Clinical pharmacology of axitinib. Clin Pharmacokinet. 2013; 52:713–25. <https://doi.org/10.1007/s40262-013-0068-3>.

4. Do K, Wilsker D, Ji J, Zlott J, Freshwater T, Kinders RJ, Collins J, Chen AP, Doroshow JH, Kummar S. Phase I Study of Single-Agent AZD1775 (MK-1775), a Wee1 Kinase Inhibitor, in Patients With Refractory Solid Tumors. J Clin Oncol. 2015; 33:3409–15. <https://doi.org/10.1200/JCO.2014.60.4009>.

5. Ramanathan RK, Von Hoff DD, Eskens F, Blumenschein GR, Richards DA, Renshaw FG, Rajagopalan P, Kelly A, Pena CE, Mross KB. Phase Ib trial of the combination of PI3K inhibitor BAY 80-6946 and allosteric-MEK inhibitor BAY 86-9766 in subjects with advanced cancer. Clinical Study Websynopsis *(BAY 80-6946 / 12876)*. 2015. https://doi.org 10.1200/jco.2014.32.15_suppl.2588.

6. Steele NL, Plumb JA, Vidal L, Tjørnelund J, Knoblauch P, Rasmussen A, Ooi CE, Buhl-Jensen P, Brown R, Evans TR, DeBono JS. A phase 1 pharmacokinetic and pharmacodynamic study of the histone deacetylase inhibitor belinostat in patients with advanced solid tumors. Clin Cancer Res. 2008; 14:804–10. <https://doi.org/10.1158/1078-0432.CCR-07-1786>.

7. Burris H, et al. First-in-man Phase I study of the oral dual PI3K and mTORC1/2 inhibitor BEZ235 in patients with advanced solid tumors. *ASCO Annual Meeting 2010*. 2010.

8. Bendell JC, Papadopoulos K,  Jones SF, Barrett E, Guthrie K, Kass CL, Litwiler KS, Napier C, Patnaik A. A phase I dose-escalation study of MEK inhibitor MEK162 (ARRY-438162) in patients with advanced solid tumors. Mol Cancer Ther. 2010; 10:B243.

9. Amaravadi RK, Schilder RJ, Martin LP, Levin M, Graham MA, Weng DE, Adjei AA. A Phase I Study of the SMAC-Mimetic Birinapant in Adults with Refractory Solid Tumors or Lymphoma. Mol Cancer Ther. 2015; 14:2569–75. <https://doi.org/10.1158/1535-7163.MCT-15-0475>.

10. Bendell JC, Rodon J, Burris HA, de Jonge M, Verweij J, Birle D, Demanse D, De Buck SS, Ru QC, Peters M, Goldbrunner M, Baselga J. Phase I, dose-escalation study of BKM120, an oral pan-Class I PI3K inhibitor, in patients with advanced solid tumors. J Clin Oncol. 2012; 30:282–90. <https://doi.org/10.1200/JCO.2011.36.1360>.

11. Janssen-Cilag. Velcade® Data Sheet. <http://www.janssen.com/newzealand/sites/www_janssen_com_newzealand/files/product/pdf/velcadeinj.pdf>. 2015.

12. Kurzrock R, Sherman SI, Ball DW, Forastiere AA, Cohen RB, Mehra R, Pfister DG, Cohen EE, Janisch L, Nauling F, Hong DS, Ng CS, Ye L, et al. Activity of XL184 (Cabozantinib), an oral tyrosine kinase inhibitor, in patients with medullary thyroid cancer. J Clin Oncol. 2011; 29:2660–66. <https://doi.org/10.1200/JCO.2010.32.4145>.

13. Papadopoulos KP, Burris HA 3rd, Gordon M, Lee P, Sausville EA, Rosen PJ, Patnaik A, Cutler RE Jr, Wang Z, Lee S, Jones SF, Infante JR. A phase I/II study of carfilzomib 2-10-min infusion in patients with advanced solid tumors. Cancer Chemother Pharmacol. 2013; 72:861–68. <https://doi.org/10.1007/s00280-013-2267-x>.

14. Shaw AT, Kim DW, Mehra R, Tan DS, Felip E, Chow LQ, Camidge DR, Vansteenkiste J, Sharma S, De Pas T, Riely GJ, Solomon BJ, Wolf J, et al. Ceritinib in ALK-rearranged non-small-cell lung cancer. N Engl J Med. 2014; 370:1189–97. <https://doi.org/10.1056/NEJMoa1311107>.

15.  FDA. COTELLIC® (cobimetinib) tablets, for oral use. U.S. Food and Drug Administration Full Prescribing Information Sheet. 2016; 1-19.

16. Mossé YP, Lim MS, Voss SD, Wilner K, Ruffner K, Laliberte J, Rolland D, Balis FM, Maris JM, Weigel BJ, Ingle AM, Ahern C, Adamson PC, Blaney SM. Safety and activity of crizotinib for paediatric patients with refractory solid tumours or anaplastic large-cell lymphoma: a Children’s Oncology Group phase 1 consortium study. Lancet Oncol. 2013; 14:472–80. <https://doi.org/10.1016/S1470-2045(13)70095-0>.

17. Younes A, Berdeja JG, Patel MR, Flinn I, Gerecitano JF, Neelapu SS, Kelly KR, Copeland AR, Akins A, Clancy MS, Gong L, Wang J, Ma A, et al. Safety, tolerability, and preliminary activity of CUDC-907, a first-in-class, oral, dual inhibitor of HDAC and PI3K, in patients with relapsed or refractory lymphoma or multiple myeloma: an open-label, dose-escalation, phase 1 trial. Lancet Oncol. 2016; 17:622–31. <https://doi.org/10.1016/S1470-2045(15)00584-7>.

18. Aplenc R, Blaney SM, Strauss LC, Balis FM, Shusterman S, Ingle AM, Agrawal S, Sun J, Wright JJ, Adamson PC. Pediatric phase I trial and pharmacokinetic study of dasatinib: a report from the children’s oncology group phase I consortium. J Clin Oncol. 2011; 29:839–44. <https://doi.org/10.1200/JCO.2010.30.7231>.

19. Nemunaitis JJ, Small KA, Kirschmeier P, Zhang D, Zhu Y, Jou YM, Statkevich P, Yao SL, Bannerji R. A first-in-human, phase 1, dose-escalation study of dinaciclib, a novel cyclin-dependent kinase inhibitor, administered weekly in subjects with advanced malignancies. J Transl Med. 2013; 11:259. <https://doi.org/10.1186/1479-5876-11-259>.

20. Bruno R, Hille D, Riva A, Vivier N, ten Bokkel Huinnink WW, van Oosterom AT, Kaye SB, Verweij J, Fossella FV, Valero V, Rigas JR, Seidman AD, Chevallier B, et al. Population pharmacokinetics/pharmacodynamics of docetaxel in phase II studies in patients with cancer. J Clin Oncol. 1998; 16:187–96. <https://doi.org/10.1200/JCO.1998.16.1.187>.

21. Greene RF, Collins JM, Jenkins JF, Speyer JL, Myers CE. Plasma pharmacokinetics of adriamycin and adriamycinol: implications for the design of in vitro experiments and treatment protocols. Cancer Res. 1983; 43:3417–21.

22.  Infinity. NCCN Request for Proposals (RFP): Phase I/II Clinical Trials and/or Correlative Studies with Duvelisib for Hematologic Malignancies. NCCN INFINITY DUVELISIB RFP. 2016.

23. Gore L, Rothenberg ML, O’Bryant CL, Schultz MK, Sandler AB, Coffin D, McCoy C, Schott A, Scholz C, Eckhardt SG. A phase I and pharmacokinetic study of the oral histone deacetylase inhibitor, MS-275, in patients with refractory solid tumors and lymphomas. Clin Cancer Res. 2008; 14:4517–25. <https://doi.org/10.1158/1078-0432.CCR-07-1461>.

24. Kaul S, Igwemezie LN, Stewart DJ, Fields SZ, Kosty M, Levithan N, Bukowski R, Gandara D, Goss G, O’Dwyer P. Pharmacokinetics and bioequivalence of etoposide following intravenous administration of etoposide phosphate and etoposide in patients with solid tumors. J Clin Oncol. 1995; 13:2835–41. <https://doi.org/10.1200/JCO.1995.13.11.2835>.

25. RxList. Zortress (Everolimus) Drug Information: Clinical Pharmacology - Prescribing Information at RxList. <http://www.rxlist.com/zortress-drug/clinical-pharmacology.htm>. 2016.

26. Daw NC, Furman WL, Stewart CF, Iacono LC, Krailo M, Bernstein ML, Dancey JE, Speights RA, Blaney SM, Croop JM, Reaman GH, Adamson PC, and Children’s Oncology Group. Phase I and pharmacokinetic study of gefitinib in children with refractory solid tumors: a Children’s Oncology Group Study. J Clin Oncol. 2005; 23:6172–80. <https://doi.org/10.1200/JCO.2005.11.429>.

27. Reid JM, Qu W, Safgren SL, Ames MM, Krailo MD, Seibel NL, Kuttesch J, Holcenberg J. Phase I trial and pharmacokinetics of gemcitabine in children with advanced solid tumors. J Clin Oncol. 2004; 22:2445–51. <https://doi.org/10.1200/JCO.2004.10.142>.

28.  Munster, P. N. *et al.* A Phase I, Open-Label, Dose-Escalation Study of the Phosphoinositide 3-Kinase Inhibitor GSK2126458 in Subjects with Solid Tumors or Lymphoma (Study Number: P3K112826). 2012.

29. Flinn IW, Kahl BS, Leonard JP, Furman RR, Brown JR, Byrd JC, Wagner-Johnston ND, Coutre SE, Benson DM, Peterman S, Cho Y, Webb HK, Johnson DM, et al. Idelalisib, a selective inhibitor of phosphatidylinositol 3-kinase-δ, as therapy for previously treated indolent non-Hodgkin lymphoma. Blood. 2014; 123:3406–13. <https://doi.org/10.1182/blood-2013-11-538546>.

30. Ghobrial IM, Siegel DS, Vij R, Berdeja JG, Richardson PG, Neuwirth R, Patel CG, Zohren F, Wolf JL. TAK-228 (formerly MLN0128), an investigational oral dual TORC1/2 inhibitor: A phase I dose escalation study in patients with relapsed or refractory multiple myeloma, non-Hodgkin lymphoma, or Waldenström’s macroglobulinemia. Am J Hematol. 2016; 91:400–05. <https://doi.org/10.1002/ajh.24300>.

31. Widemann BC, Goodspeed W, Goodwin A, Fojo T, Balis FM, Fox E. Phase I trial and pharmacokinetic study of ixabepilone administered daily for 5 days in children and adolescents with refractory solid tumors. J Clin Oncol. 2009; 27:550–56. <https://doi.org/10.1200/JCO.2008.17.6644>.

32. Fouladi M, Stewart CF, Blaney SM, Onar-Thomas A, Schaiquevich P, Packer RJ, Gajjar A, Kun LE, Boyett JM, Gilbertson RJ. Phase I trial of lapatinib in children with refractory CNS malignancies: a Pediatric Brain Tumor Consortium study. J Clin Oncol. 2010; 28:4221–27. <https://doi.org/10.1200/JCO.2010.28.4687>.

33. Calvo E, Chen VJ, Marshall M, Ohnmacht U, Hynes SM, Kumm E, Diaz HB, Barnard D, Merzoug FF, Huber L, Kays L, Iversen P, Calles A, et al. Preclinical analyses and phase I evaluation of LY2603618 administered in combination with pemetrexed and cisplatin in patients with advanced cancer. Invest New Drugs. 2014; 32:955–68. <https://doi.org/10.1007/s10637-014-0114-5>.

34. Lévy V, Zohar S, Bardin C, Vekhoff A, Chaoui D, Rio B, Legrand O, Sentenac S, Rousselot P, Raffoux E, Chast F, Chevret S, Marie JP. A phase I dose-finding and pharmacokinetic study of subcutaneous semisynthetic homoharringtonine (ssHHT) in patients with advanced acute myeloid leukaemia. Br J Cancer. 2006; 95:253–59. <https://doi.org/10.1038/sj.bjc.6603265>.

35. Schwartz GK, LoRusso PM, Dickson MA, Randolph SS, Shaik MN, Wilner KD, Courtney R, O'Dwyer PJ. Phase I study of PD 0332991, a cyclin-dependent kinase inhibitor, administered in 3-week cycles (Schedule 2/1). Br J Cancer. 2011; 104:1862-8.

36. Mu S, Kuroda Y, Shibayama H, Hino M, Tajima T, Corrado C, Lin R, Waldron E, Binlich F, Suzuki K. Panobinostat PK/PD profile in combination with bortezomib and dexamethasone in patients with relapsed and relapsed/refractory multiple myeloma. Eur J Clin Pharmacol. 2016; 72:153–61. <https://doi.org/10.1007/s00228-015-1967-z>.

37. Glade Bender JL, Lee A, Reid JM, Baruchel S, Roberts T, Voss SD, Wu B, Ahern CH, Ingle AM, Harris P, Weigel BJ, Blaney SM. Phase I pharmacokinetic and pharmacodynamic study of pazopanib in children with soft tissue sarcoma and other refractory solid tumors: a children’s oncology group phase I consortium report. J Clin Oncol. 2013; 31:3034–43. <https://doi.org/10.1200/JCO.2012.47.0914>.

38. LoRusso PM, Krishnamurthi SS, Rinehart JJ, Nabell LM, Malburg L, Chapman PB, DePrimo SE, Bentivegna S, Wilner KD, Tan W, Ricart AD. Phase I pharmacokinetic and pharmacodynamic study of the oral MAPK/ERK kinase inhibitor PD-0325901 in patients with advanced cancers. Clin Cancer Res. 2010; 16:1924–37. <https://doi.org/10.1158/1078-0432.CCR-09-1883>.

39. Malempati S, Nicholson HS, Reid JM, Blaney SM, Ingle AM, Krailo M, Stork LC, Melemed AS, McGovern R, Safgren S, Ames MM, Adamson PC, and Children’s Oncology Group. Phase I trial and pharmacokinetic study of pemetrexed in children with refractory solid tumors: the Children’s Oncology Group. J Clin Oncol. 2007; 25:1505–11. <https://doi.org/10.1200/JCO.2006.09.1694>.

40. Stewart DA, Smith C, MacFarland R, Calandra G. Pharmacokinetics and pharmacodynamics of plerixafor in patients with non-Hodgkin lymphoma and multiple myeloma. Biol Blood Marrow Transplant. 2009; 15:39–46. <https://doi.org/10.1016/j.bbmt.2008.10.018>.

41. RxList. Stivarga (Regorafenib Tablets) Drug Information: Clinical Pharmacology - Prescribing Information at RxList. <http://www.rxlist.com/stivarga-drug/clinical-pharmacology.htm>. 2016.

42. Fouladi M, Furman WL, Chin T, Freeman BB 3rd, Dudkin L, Stewart CF, Krailo MD, Speights R, Ingle AM, Houghton PJ, Wright J, Adamson PC, Blaney SM, and Children’s Oncology Group. Phase I study of depsipeptide in pediatric patients with refractory solid tumors: a Children’s Oncology Group report. J Clin Oncol. 2006; 24:3678–85. <https://doi.org/10.1200/JCO.2006.06.4964>.

43. Shapiro GI, Rodon J, Bedell C, Kwak EL, Baselga J, Braña I, Pandya SS, Scheffold C, Laird AD, Nguyen LT, Xu Y, Egile C, Edelman G. Phase I safety, pharmacokinetic, and pharmacodynamic study of SAR245408 (XL147), an oral pan-class I PI3K inhibitor, in patients with advanced solid tumors. Clin Cancer Res. 2014; 20:233–45. <https://doi.org/10.1158/1078-0432.CCR-13-1777>.

44. Alexander TB, Lacayo NJ, Choi JK, Ribeiro RC, Pui CH, Rubnitz JE. Phase I Study of Selinexor, a Selective Inhibitor of Nuclear Export, in Combination With Fludarabine and Cytarabine, in Pediatric Relapsed or Refractory Acute Leukemia. J Clin Oncol. 2016; 34:4094–101. <https://doi.org/10.1200/JCO.2016.67.5066>.

45. Leijen S, Soetekouw PM, Jeffry Evans TR, Nicolson M, Schellens JH, Learoyd M, Grinsted L, Zazulina V, Pwint T, Middleton M. A phase I, open-label, randomized crossover study to assess the effect of dosing of the MEK 1/2 inhibitor Selumetinib (AZD6244; ARRY-142866) in the presence and absence of food in patients with advanced solid tumors. Cancer Chemother Pharmacol. 2011; 68:1619–28. <https://doi.org/10.1007/s00280-011-1732-7>.

46. Adjei AA, Cohen RB, Franklin W, Morris C, Wilson D, Molina JR, Hanson LJ, Gore L, Chow L, Leong S, Maloney L, Gordon G, Simmons H, et al. Phase I pharmacokinetic and pharmacodynamic study of the oral, small-molecule mitogen-activated protein kinase kinase 1/2 inhibitor AZD6244 (ARRY-142886) in patients with advanced cancers. J Clin Oncol. 2008; 26:2139–46. <https://doi.org/10.1200/JCO.2007.14.4956>.

47. Ma MK, Zamboni WC, Radomski KM, Furman WL, Santana VM, Houghton PJ, Hanna SK, Smith AK, Stewart CF. Pharmacokinetics of irinotecan and its metabolites SN-38 and APC in children with recurrent solid tumors after protracted low-dose irinotecan. Clin Cancer Res. 2000; 6:813–19.

48. Brendel E, Ludwig M, Lathia C, Robert C, Ropert S, Soria JC, Armand JP. Pharmacokinetic results of a phase I trial of sorafenib in combination with dacarbazine in patients with advanced solid tumors. Cancer Chemother Pharmacol. 2011; 68:53–61. <https://doi.org/10.1007/s00280-010-1423-9>.

49. Bagatell R, Gore L, Egorin MJ, Ho R, Heller G, Boucher N, Zuhowski EG, Whitlock JA, Hunger SP, Narendran A, Katzenstein HM, Arceci RJ, Boklan J, et al. Phase I pharmacokinetic and pharmacodynamic study of 17-N-allylamino-17-demethoxygeldanamycin in pediatric patients with recurrent or refractory solid tumors: a pediatric oncology experimental therapeutics investigators consortium study. Clin Cancer Res. 2007; 13:1783–88. <https://doi.org/10.1158/1078-0432.CCR-06-1892>.

50. Horton TM, Thompson PA, Berg SL, Adamson PC, Ingle AM, Dolan ME, Delaney SM, Hedge M, Weiss HL, Wu MF, Blaney SM, and Children’s Oncology Group Study. Phase I pharmacokinetic and pharmacodynamic study of temozolomide in pediatric patients with refractory or recurrent leukemia: a Children’s Oncology Group Study. J Clin Oncol. 2007; 25:4922–28. <https://doi.org/10.1200/JCO.2007.12.0667>.

51. Spunt SL, Grupp SA, Vik TA, Santana VM, Greenblatt DJ, Clancy J, Berkenblit A, Krygowski M, Ananthakrishnan R, Boni JP, Gilbertson RJ. Phase I study of temsirolimus in pediatric patients with recurrent/refractory solid tumors. J Clin Oncol. 2011; 29:2933–40. <https://doi.org/10.1200/JCO.2010.33.4649>.

52.  GlaxoSmithKline. Mekinist® Tablets Product Information. GlaxoSmithKline Australia Pty Ltd. 2014; 1-25.

53. Gordon MS, Rosen LS, Mendelson D, Ramanathan RK, Goldman J, Liu L, Xu Y, Gerson SL, Anthony SP, Figg WD, Spencer S, Adams BJ, Theuer CP, et al. A phase 1 study of TRC102, an inhibitor of base excision repair, and pemetrexed in patients with advanced solid tumors. Invest New Drugs. 2013; 31:714–23. <https://doi.org/10.1007/s10637-012-9876-9>.

54. Murren J, Modiano M, Clairmont C, Lambert P, Savaraj N, Doyle T, Sznol M. Phase I and pharmacokinetic study of triapine, a potent ribonucleotide reductase inhibitor, administered daily for five days in patients with advanced solid tumors. Clin Cancer Res. 2003; 9:4092–100.

55. Broniscer A, Baker JN, Tagen M, Onar-Thomas A, Gilbertson RJ, Davidoff AM, Pai Panandiker AS, Leung W, Chin TK, Stewart CF, Kocak M, Rowland C, Merchant TE, et al. Phase I study of vandetanib during and after radiotherapy in children with diffuse intrinsic pontine glioma. J Clin Oncol. 2010; 28:4762–68. <https://doi.org/10.1200/JCO.2010.30.3545>.

56. Grippo JF, Zhang W, Heinzmann D, Yang KH, Wong J, Joe AK, Munster P, Sarapa N, Daud A. A phase I, randomized, open-label study of the multiple-dose pharmacokinetics of vemurafenib in patients with BRAF V600E mutation-positive metastatic melanoma. Cancer Chemother Pharmacol. 2014; 73:103–11. <https://doi.org/10.1007/s00280-013-2324-5>.

57. Gajjar A, Stewart CF, Ellison DW, Kaste S, Kun LE, Packer RJ, Goldman S, Chintagumpala M, Wallace D, Takebe N, Boyett JM, Gilbertson RJ, Curran T. Phase I study of vismodegib in children with recurrent or refractory medulloblastoma: a pediatric brain tumor consortium study. Clin Cancer Res. 2013; 19:6305–12. <https://doi.org/10.1158/1078-0432.CCR-13-1425>.
