# Supplementary material for: Neurofibromin level directs RAS pathway signaling and mediates sensitivity to targeted agents in malignant peripheral nerve sheath tumors
Source: Oncotarget. 2018 Apr 27;9(32):22571–85. doi: 10.18632/oncotarget.25181 (PMC5978249; doi:10.18632/oncotarget.25181)
Supplement: Supplementary file 3 [file oncotarget-09-22571-s003.pdf]

*Kahen, E., et al. 2018*

# Supplementary Table S2: Single Agent Screen Data

| Drug | Concentration (ng/ml) | | | MPNST Cell Line | | | | | | | | | | | | Average | | | Standard Error | | |
|---|---|---|---|---|---|---|---|---|---|---|---|---|---|---|---|---|---|---|---|---|---|
| | | | | SNF02.2 | | | SNF10.1 | | | SNF94.3 | | | SNF96.2 | | | | | | | | |
| | Top | 1:5 | 1:25 | Top | 1:5 | 1:25 | Top | 1:5 | 1:25 | Top | 1:5 | 1:25 | Top | 1:5 | 1:25 | Top | 1:5 | 1:25 | Top | 1:5 | 1:25 |
| Bortezomib | 163.96 | 32.79 | 6.56 | 0.981 | 0.951 | 0.647 | 0.992 | 0.985 | 0.968 | 0.971 | 0.947 | 0.765 | 0.99 | 0.976 | 0.651 | 0.983 | 0.965 | 0.751 | 633.3E-6 | 1.7E-3 | 5.0E-3 |
| Doxorubicin | 68.97 | 13.79 | 2.76 | 0.911 | 0.432 | 0.215 | 0.981 | 0.815 | 0.556 | 0.845 | 0.449 | 0.249 | 0.969 | 0.745 | 0.44 | 0.926 | 0.626 | 0.380 | 4.9E-3 | 5.3E-3 | 6.9E-3 |
| BAY 80-6946 | 674.27 | 134.85 | 26.97 | 0.67 | 0.56 | 0.367 | 0.954 | 0.923 | 0.685 | 0.749 | 0.631 | 0.425 | 0.968 | 0.79 | 0.485 | 0.852 | 0.722 | 0.459 | 7.9E-3 | 8.2E-3 | 12.4E-3 |
| Palifosfamide | 45244.77 | 9048.95 | 1809.79 | 0.758 | 0.336 | 0.106 | 0.948 | 0.8 | 0.515 | 0.824 | 0.491 | 0.198 | 0.816 | 0.475 | 0.15 | 0.843 | 0.540 | 0.250 | 4.8E-3 | 6.3E-3 | 13.9E-3 |
| Dinaciclib | 353.10 | 1765.49 | 70.62 | 0.672 | 0.692 | 0.333 | 0.966 | 0.971 | 0.649 | 0.716 | 0.702 | 0.361 | 0.958 | 0.957 | 0.523 | 0.836 | 0.835 | 0.468 | 5.1E-3 | 2.1E-3 | 36.0E-3 |
| Romidepsin | 697.24 | 139.45 | 27.89 | 0.684 | 0.457 | 0.223 | 0.967 | 0.824 | 0.519 | 0.704 | 0.457 | 0.212 | 0.887 | 0.506 | 0.232 | 0.810 | 0.557 | 0.298 | 12.2E-3 | 14.6E-3 | 12.0E-3 |
| BKM120 | 2361.17 | 472.23 | 94.45 | 0.624 | 0.224 | 0.052 | 0.911 | 0.545 | 0.206 | 0.738 | 0.239 | 0.055 | 0.892 | 0.351 | 0.087 | 0.796 | 0.330 | 0.094 | 8.5E-3 | 5.1E-3 | 5.9E-3 |
| Ixabepilone | 197.36 | 39.47 | 7.89 | 0.64 | 0.544 | 0.371 | 0.882 | 0.856 | 0.62 | 0.705 | 0.632 | 0.475 | 0.841 | 0.791 | 0.487 | 0.786 | 0.730 | 0.497 | 6.5E-3 | 8.9E-3 | 11.8E-3 |
| Docetaxel | 495.12 | 99.02 | 2475.62 | 0.619 | 0.603 | 0.558 | 0.882 | 0.879 | 0.887 | 0.673 | 0.662 | 0.651 | 0.851 | 0.829 | 0.861 | 0.777 | 0.762 | 0.764 | 7.6E-3 | 6.2E-3 | 9.4E-3 |
| INK128 | 161.64 | 32.33 | 6.47 | 0.638 | 0.484 | 0.236 | 0.844 | 0.813 | 0.526 | 0.684 | 0.564 | 0.272 | 0.866 | 0.608 | 0.229 | 0.761 | 0.615 | 0.310 | 6.5E-3 | 5.9E-3 | 4.9E-3 |
| BEZ235 | 1000.96 | 200.19 | 40.04 | 0.602 | 0.592 | 0.518 | 0.881 | 0.864 | 0.81 | 0.671 | 0.671 | 0.596 | 0.863 | 0.85 | 0.726 | 0.758 | 0.748 | 0.664 | 8.6E-3 | 8.8E-3 | 8.4E-3 |
| Omacetaxine | 183.28 | 36.66 | 7.33 | 0.705 | 0.335 | 0.01 | 0.779 | 0.62 | 0.276 | 0.729 | 0.479 | 0.108 | 0.812 | 0.568 | 0.081 | 0.758 | 0.499 | 0.111 | 8.4E-3 | 6.5E-3 | 13.0E-3 |
| Gemcitabine | 136821.73 | 27364.35 | 5472.87 | 0.611 | 0.577 | 0.542 | 0.93 | 0.916 | 0.858 | 0.647 | 0.621 | 0.596 | 0.785 | 0.726 | 0.731 | 0.756 | 0.719 | 0.694 | 5.1E-3 | 3.3E-3 | 5.3E-3 |
| GSK2126458 | 83.28 | 16.66 | 3.33 | 0.55 | 0.364 | 0.183 | 0.88 | 0.687 | 0.428 | 0.63 | 0.428 | 0.237 | 0.729 | 0.502 | 0.286 | 0.709 | 0.505 | 0.293 | 16.3E-3 | 17.6E-3 | 10.8E-3 |
| Panobinostat | 60.68 | 12.14 | 2.43 | 0.523 | 0.222 | 0.01 | 0.92 | 0.586 | 0.233 | 0.559 | 0.214 | 0.007 | 0.748 | 0.261 | 0.018 | 0.689 | 0.324 | 0.064 | 4.9E-3 | 3.7E-3 | 4.4E-3 |
| Belinostat | 100907.81 | 20181.56 | 4036.31 | 0.413 | 0.125 | 0.029 | 0.805 | 0.411 | 0.131 | 0.494 | 0.086 | -0.018 | 0.691 | 0.244 | 0.073 | 0.620 | 0.215 | 0.054 | 23.0E-3 | 11.7E-3 | 23.3E-3 |
| Alisertib | 14453.09 | 2890.62 | 578.12 | 0.322 | 0.207 | 0.147 | 0.85 | 0.724 | 0.641 | 0.403 | 0.288 | 0.242 | 0.622 | 0.507 | 0.448 | 0.572 | 0.450 | 0.387 | 12.5E-3 | 12.2E-3 | 8.8E-3 |
| SN-38 | 25.48 | 5.10 | 1.02 | 0.35 | 0.138 | 0.026 | 0.864 | 0.664 | 0.13 | 0.396 | 0.147 | 0.059 | 0.541 | 0.37 | 0.105 | 0.555 | 0.353 | 0.084 | 6.0E-3 | 11.2E-3 | 4.0E-3 |
| Etoposide | 8494.73 | 1698.95 | 339.79 | 0.309 | 0.193 | 0.134 | 0.835 | 0.618 | 0.336 | 0.349 | 0.21 | 0.146 | 0.598 | 0.453 | 0.267 | 0.546 | 0.388 | 0.226 | 13.6E-3 | 10.3E-3 | 2.8E-3 |
| Duvelisib | 7736.41 | 1547.28 | 309.46 | 0.425 | 0.322 | 0.206 | 0.611 | 0.338 | 0.173 | 0.535 | 0.435 | 0.285 | 0.505 | 0.264 | 0.14 | 0.532 | 0.339 | 0.201 | 9.4E-3 | 16.2E-3 | 12.9E-3 |
| Vemurafenib | 63278.22 | 12655.64 | 2531.13 | 0.417 | 0.086 | -0.003 | 0.794 | 0.32 | -0.011 | 0.417 | 0.062 | -0.017 | 0.479 | -0.029 | -0.082 | 0.524 | 0.099 | -0.033 | 9.6E-3 | 11.4E-3 | 8.2E-3 |
| Cabozantinib | 2811.51 | 562.30 | 112.46 | 0.531 | 0.463 | 0.381 | 0.281 | 0.112 | 0.139 | 0.613 | 0.542 | 0.444 | 0.427 | 0.337 | 0.275 | 0.473 | 0.371 | 0.307 | 7.5E-3 | 5.8E-3 | 5.0E-3 |
| Temsirolimus | 97.64 | 488.21 | 19.53 | 0.336 | 0.343 | 0.325 | 0.51 | 0.527 | 0.501 | 0.414 | 0.425 | 0.408 | 0.379 | 0.379 | 0.361 | 0.406 | 0.416 | 0.396 | 6.8E-3 | 11.4E-3 | 5.5E-3 |
| Everolimus | 11.58 | 2.32 | 0.46 | 0.314 | 0.289 | 0.166 | 0.417 | 0.425 | 0.344 | 0.391 | 0.366 | 0.232 | 0.323 | 0.301 | 0.188 | 0.364 | 0.344 | 0.226 | 9.1E-3 | 8.3E-3 | 13.6E-3 |
| Pemetrexed | 893141.27 | 178628.25 | 35725.65 | 0.34 | 0.323 | 0.251 | 0.301 | 0.221 | -0.004 | 0.415 | 0.359 | 0.262 | 0.293 | 0.196 | 0.042 | 0.327 | 0.262 | 0.123 | 27.7E-3 | 25.9E-3 | 32.4E-3 |
| PD0325901 | 230.20 | 46.04 | 9.21 | 0.29 | 0.216 | 0.13 | 0.296 | 0.205 | 0.146 | 0.251 | 0.173 | 0.083 | 0.44 | 0.323 | 0.218 | 0.320 | 0.229 | 0.145 | 7.0E-3 | 2.9E-3 | 3.7E-3 |
| Ceritinib | 1433.33 | 286.67 | 57.33 | 0.155 | 0.095 | 0.042 | 0.64 | 0.28 | 0.111 | 0.095 | 0.017 | 0.002 | 0.356 | 0.127 | 0.042 | 0.317 | 0.124 | 0.048 | 9.7E-3 | 8.8E-3 | 1.1E-3 |
| Dasatinib | 321.71 | 64.34 | 12.87 | 0.114 | 0.027 | 0.048 | 0.516 | 0.323 | 0.181 | 0.113 | 0.024 | 0.042 | 0.476 | 0.224 | 0.105 | 0.295 | 0.141 | 0.080 | 12.2E-3 | 15.0E-3 | 25.9E-3 |
| Tanespimycin | 14567.43 | 2913.49 | 582.70 | 0.487 | 0.111 | 0.003 | 0.164 | -0.09 | -0.084 | 0.4 | 0.05 | -0.023 | 0.032 | -0.13 | -0.126 | 0.286 | -0.013 | -0.059 | 14.2E-3 | 20.0E-3 | 33.6E-3 |
| Selumetinib | 1763.24 | 352.65 | 70.53 | 0.248 | 0.178 | 0.101 | 0.205 | 0.154 | 0.132 | 0.238 | 0.156 | 0.089 | 0.408 | 0.276 | 0.179 | 0.286 | 0.195 | 0.124 | 4.8E-3 | 1.5E-3 | 1.3E-3 |
| LY2603618 | 6188.40 | 1237.68 | 247.54 | 0.154 | 0.031 | 0.011 | 0.474 | 0.259 | 0.037 | 0.244 | 0.062 | 0.011 | 0.283 | 0.108 | -0.003 | 0.283 | 0.108 | 0.011 | 2.5E-3 | 14.7E-3 | 20.2E-3 |
| CAL-101 | 7943.77 | 1588.75 | 317.75 | 0.3 | 0.152 | 0.076 | 0.165 | 0.095 | 0.102 | 0.363 | 0.225 | 0.132 | 0.246 | 0.13 | 0.085 | 0.280 | 0.158 | 0.103 | 11.3E-3 | 8.7E-3 | 15.0E-3 |
| Carfilzomib | 138.91 | 27.78 | 5.56 | 0.095 | 0.023 | 0.022 | 0.602 | 0.298 | 0.037 | 0.208 | 0.067 | -0.002 | 0.2 | 0.036 | 0.04 | 0.270 | 0.099 | 0.018 | 20.6E-3 | 19.9E-3 | 28.1E-3 |
| Pazopanib | 91424.39 | 18284.88 | 3656.98 | 0.205 | 0.194 | 0.166 | 0.456 | 0.403 | 0.176 | 0.225 | 0.178 | 0.179 | 0.187 | 0.061 | 0.017 | 0.269 | 0.199 | 0.126 | 19.0E-3 | 10.7E-3 | 4.3E-3 |
| Selinexor | 924.86 | 184.97 | 36.99 | 0.076 | 0.019 | 0.007 | 0.627 | 0.675 | 0.11 | 0.133 | 0.06 | -0.015 | 0.21 | 0.104 | -0.011 | 0.248 | 0.211 | 0.018 | 47.3E-3 | 13.8E-3 | 17.2E-3 |
| Palbociclib | 264.47 | 52.89 | 10.58 | 0.19 | 0.142 | 0.092 | 0.344 | 0.333 | 0.271 | 0.197 | 0.156 | 0.066 | 0.259 | 0.194 | 0.111 | 0.244 | 0.200 | 0.122 | 8.8E-3 | 6.6E-3 | 11.4E-3 |
| Sorafenib | 7314.66 | 292.59 | 1462.93 | -0.017 | -0.015 | -0.124 | 0.513 | 0.241 | 0.166 | 0.066 | -0.03 | -0.116 | 0.367 | -0.057 | -0.161 | 0.242 | 0.024 | -0.085 | 36.2E-3 | 18.0E-3 | 22.9E-3 |
| Vandetanib | 1573.58 | 314.72 | 62.94 | 0.215 | 0.063 | 0.017 | 0.45 | 0.109 | 0.064 | 0.179 | 0.034 | -0.005 | 0.173 | 0.011 | -0.016 | 0.240 | 0.045 | 0.013 | 9.6E-3 | 10.2E-3 | 4.8E-3 |
| Regorafenib | 8077.54 | 1615.51 | 323.10 | 0.036 | -0.071 | -0.041 | 0.481 | 0.05 | 0.093 | 0.114 | -0.013 | 0.015 | 0.257 | 0.027 | 0.024 | 0.230 | 0.007 | 0.023 | 19.9E-3 | 8.8E-3 | 8.3E-3 |
| Gefitinib | 4945.18 | 197.81 | 989.04 | 0.172 | 0.004 | 0.008 | 0.499 | 0.115 | 0.144 | 0.136 | 0.01 | -0.011 | 0.108 | 0.025 | 0.042 | 0.221 | 0.030 | 0.030 | 4.4E-3 | 4.2E-3 | 6.7E-3 |
| Triapine | 7989.76 | 1597.95 | 319.59 | 0.089 | 0.013 | -0.006 | 0.37 | 0.292 | 0.016 | 0.112 | 0.016 | -0.032 | 0.185 | 0.065 | -0.019 | 0.185 | 0.089 | -0.022 | 9.7E-3 | 23.8E-3 | 19.2E-3 |
| MK1775 | 998.80 | 199.76 | 39.95 | 0.029 | -0.002 | -0.003 | 0.252 | 0.107 | 0.016 | 0.049 | 0.013 | -0.017 | 0.142 | 0.068 | 0.011 | 0.115 | 0.035 | -0.007 | 22.1E-3 | 24.0E-3 | 23.8E-3 |
| Etinostat | 2.02 | 50.48 | 10.10 | 0.034 | 0.05 | 0.025 | 0.097 | 0.123 | 0.132 | -0.008 | -0.021 | -0.026 | 0.051 | 0.04 | 0.024 | 0.038 | 0.047 | 0.033 | 9.9E-3 | 11.5E-3 | 9.0E-3 |
| Binimetinib | 1117.33 | 223.47 | 44.69 | 0.027 | 0 | 0.005 | 0.017 | -0.017 | -0.033 | 0.013 | -0.019 | -0.022 | 0.073 | 0.027 | -0.01 | 0.027 | -0.008 | -0.018 | 24.7E-3 | 27.0E-3 | 23.6E-3 |
| Temozolomide | 2678.34 | 66958.54 | 13391.71 | 0.037 | -0.006 | -0.003 | 0.12 | 0.071 | 0.043 | -0.014 | -0.016 | -0.036 | 0.002 | -0.002 | -0.01 | 0.022 | 0.005 | -0.011 | 25.3E-3 | 32.0E-3 | 22.4E-3 |
| Lapatinib | 5672.85 | 226.91 | 1134.57 | 0.007 | 0.016 | -0.011 | 0.059 | 0.02 | 0.054 | 0.022 | -0.007 | -0.015 | 0.02 | 0.02 | 0.052 | 0.021 | 0.007 | 0.010 | 27.2E-3 | 26.5E-3 | 26.3E-3 |
| XL147 | 30990.81 | 1239.63 | 6198.16 | 0.026 | -0.007 | 0.015 | -0.033 | 0.123 | 0.061 | 0.026 | 0.02 | 0.017 | 0.033 | 0.019 | 0.035 | 0.017 | 0.029 | 0.027 | 5.5E-3 | 11.2E-3 | 11.0E-3 |
| Azacitadine | 614.25 | 122.85 | 3071.25 | -0.011 | 0.005 | -0.024 | 0.035 | 0.039 | 0.044 | 0.024 | 0.007 | -0.003 | 0.039 | 0.011 | 0.027 | 0.013 | 0.008 | 0.005 | 21.1E-3 | 23.9E-3 | 19.5E-3 |
| Crizotinib | 1398.94 | 55.96 | 279.79 | -0.183 | -0.13 | -0.178 | 0.256 | 0.162 | 0.18 | -0.151 | -0.074 | -0.106 | 0.137 | 0.033 | 0.098 | 0.010 | -0.008 | -0.013 | 24.3E-3 | 20.9E-3 | 21.1E-3 |
| TRC102 | 99.63 | 498.14 | 2490.72 | -0.004 | -0.009 | -0.007 | 0.085 | 0.032 | -0.052 | -0.046 | -0.045 | -0.035 | 0.007 | -0.026 | -0.033 | 0.008 | -0.008 | -0.025 | 8.8E-3 | 7.2E-3 | 9.4E-3 |
| Axitinib | 2.88 | 14.39 | 71.93 | -0.008 | -0.008 | 0.002 | 0.033 | 0.009 | -0.037 | 0.004 | 0.004 | 0.029 | 0.044 | 0.002 | -0.032 | 0.006 | -0.008 | -0.016 | 25.6E-3 | 15.9E-3 | 18.9E-3 |
| Vismodegib | 671.92 | 3359.60 | 16798.01 | -0.017 | -0.025 | -0.016 | 0.045 | 0.092 | 0.167 | -0.024 | -0.059 | -0.085 | -0.013 | -0.092 | -0.158 | -0.005 | -0.030 | -0.030 | 4.6E-3 | 9.1E-3 | 12.7E-3 |
| Plerixafor | 1841.69 | 73.67 | 368.34 | -0.03 | -0.008 | -0.042 | 0.019 | 0.007 | 0.018 | -0.032 | -0.009 | -0.038 | -0.002 | -0.003 | -0.005 | -0.013 | -0.015 | -0.027 | 28.3E-3 | 27.4E-3 | 23.1E-3 |
| Birinapant | 198.28 | 991.40 | 4957.00 | -0.019 | -0.056 | -0.075 | -0.033 | -0.058 | -0.147 | -0.049 | -0.068 | -0.065 | -0.074 | -0.131 | -0.189 | -0.049 | -0.085 | -0.123 | 29.2E-3 | 27.2E-3 | 25.9E-3 |
