# Supplementary figures and images for: Neurofibromin level directs RAS pathway signaling and mediates sensitivity to targeted agents in malignant peripheral nerve sheath tumors
Source: Oncotarget. 2018 Apr 27;9(32):22571–85. doi: 10.18632/oncotarget.25181 (PMC5978249; doi:10.18632/oncotarget.25181)

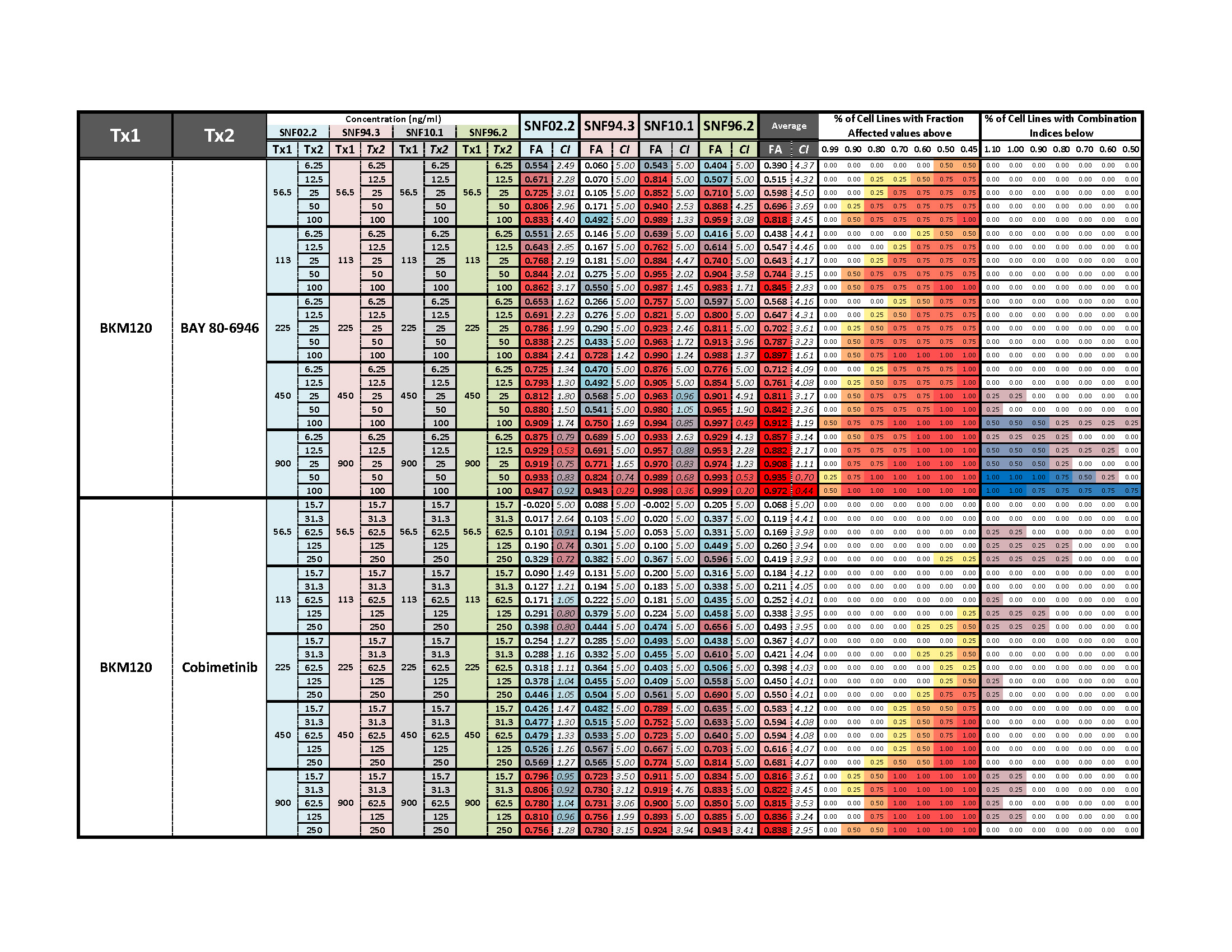

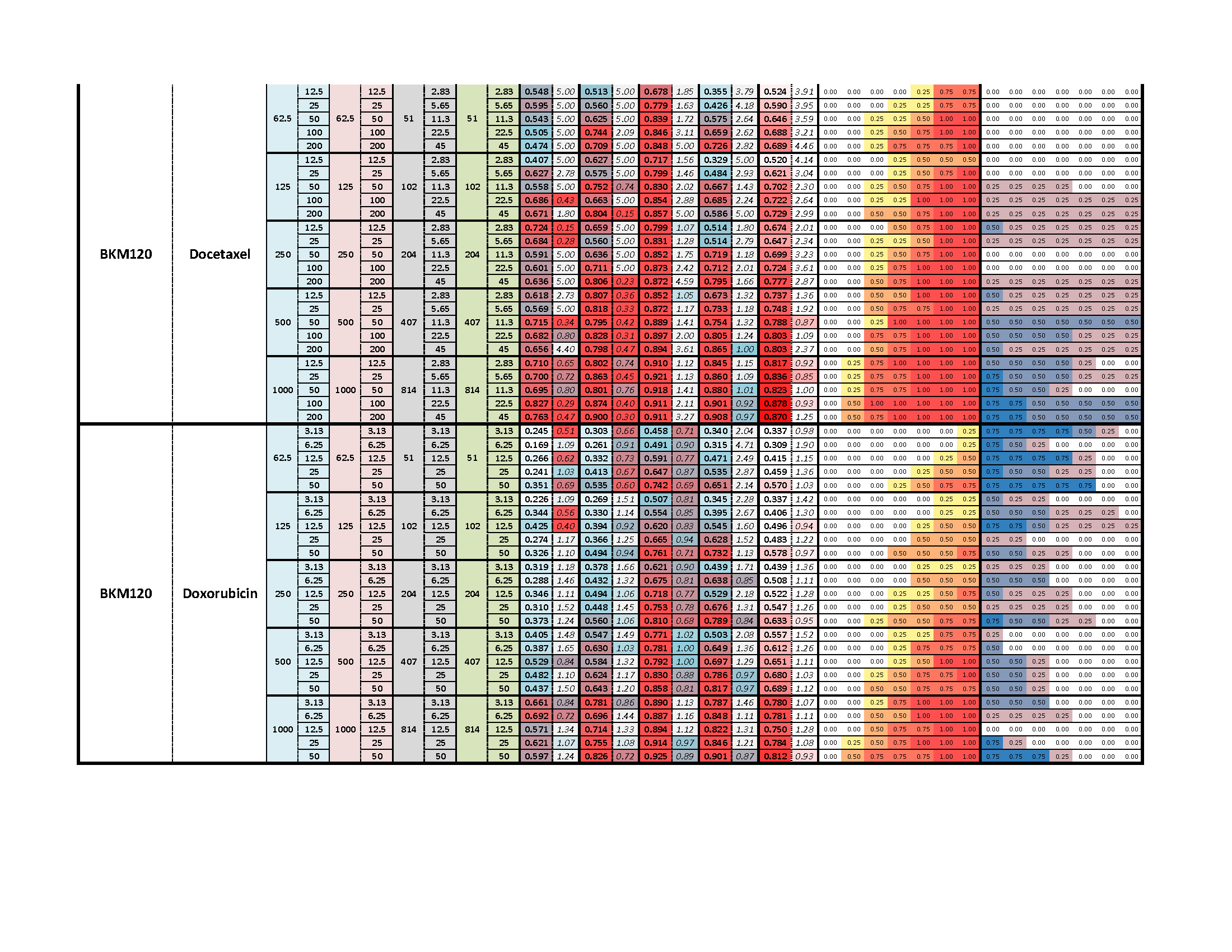

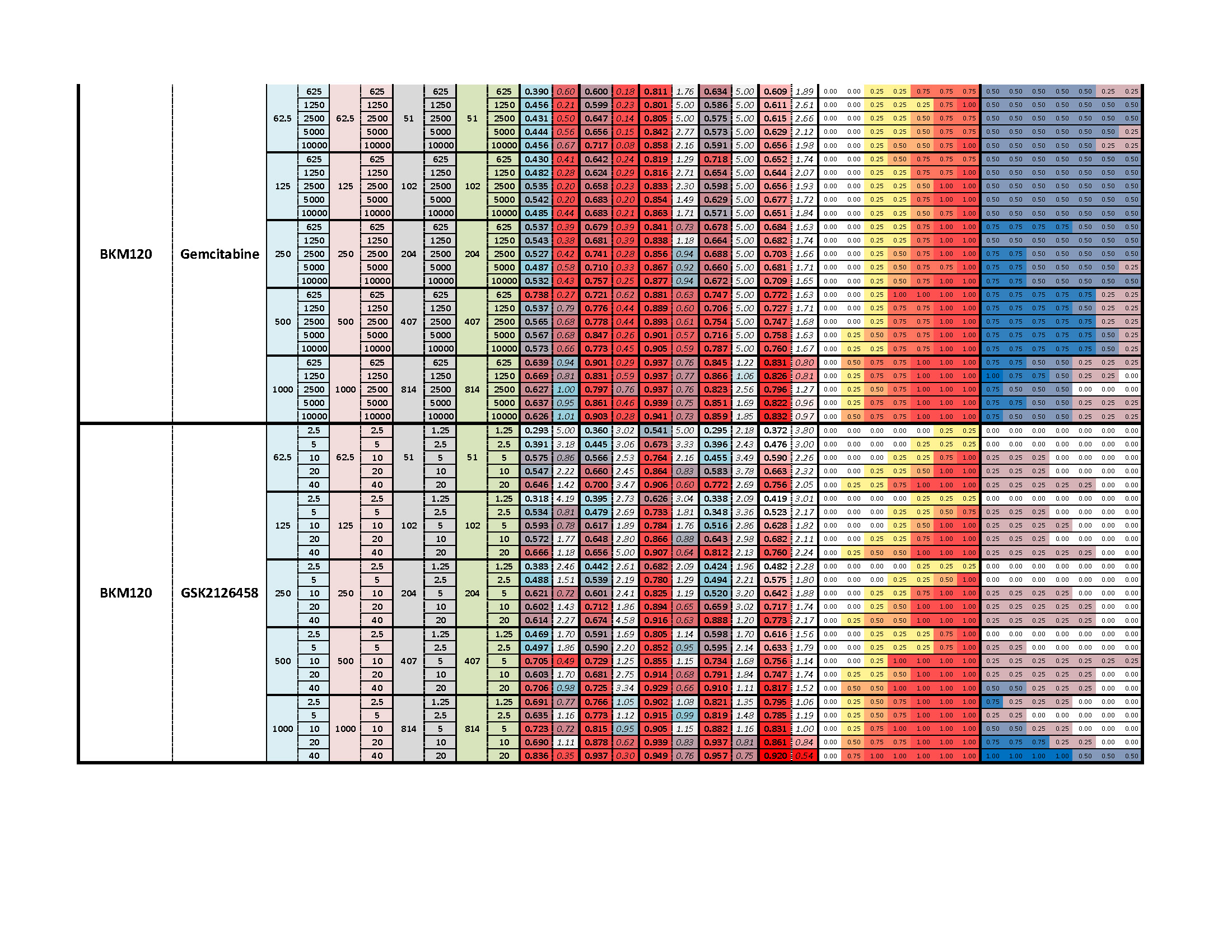


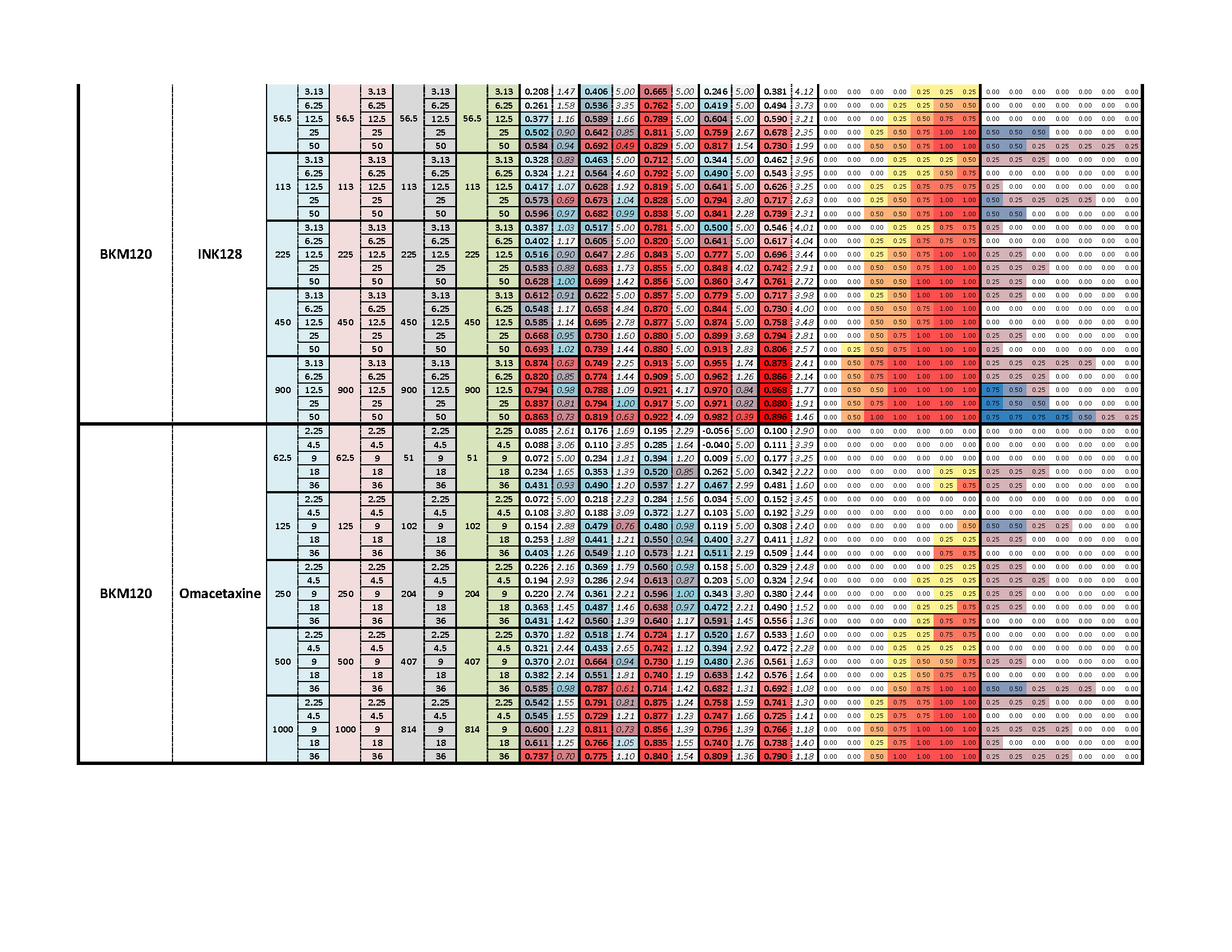

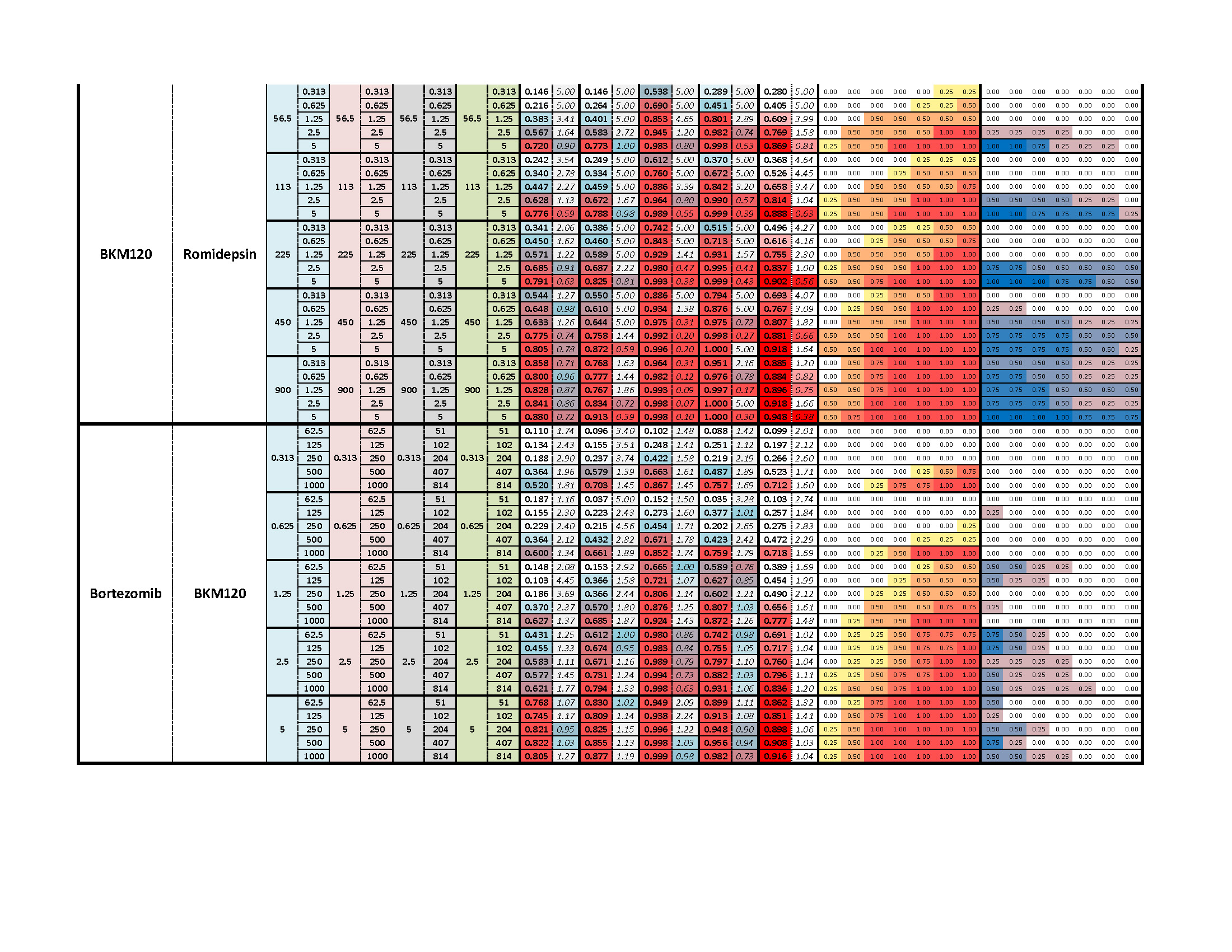


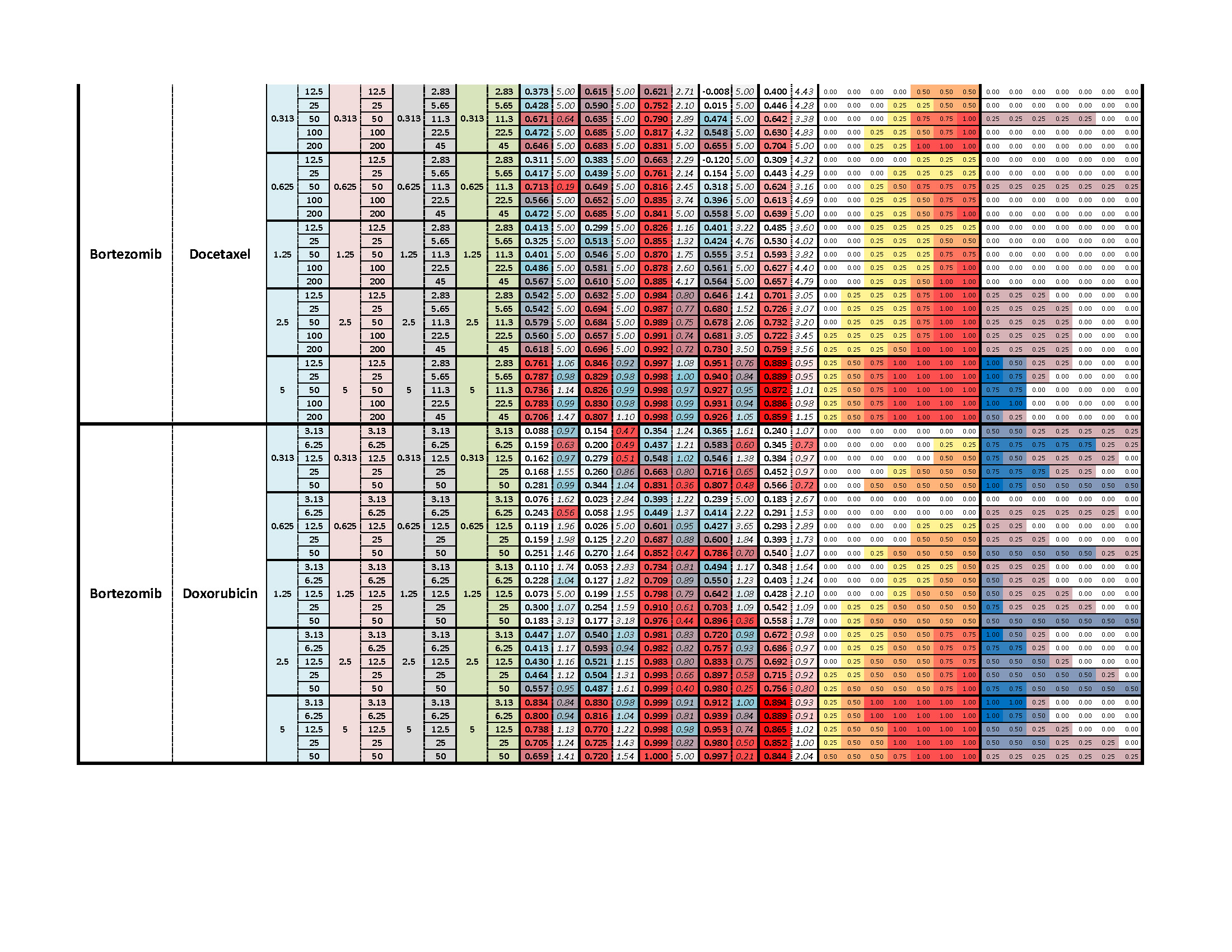

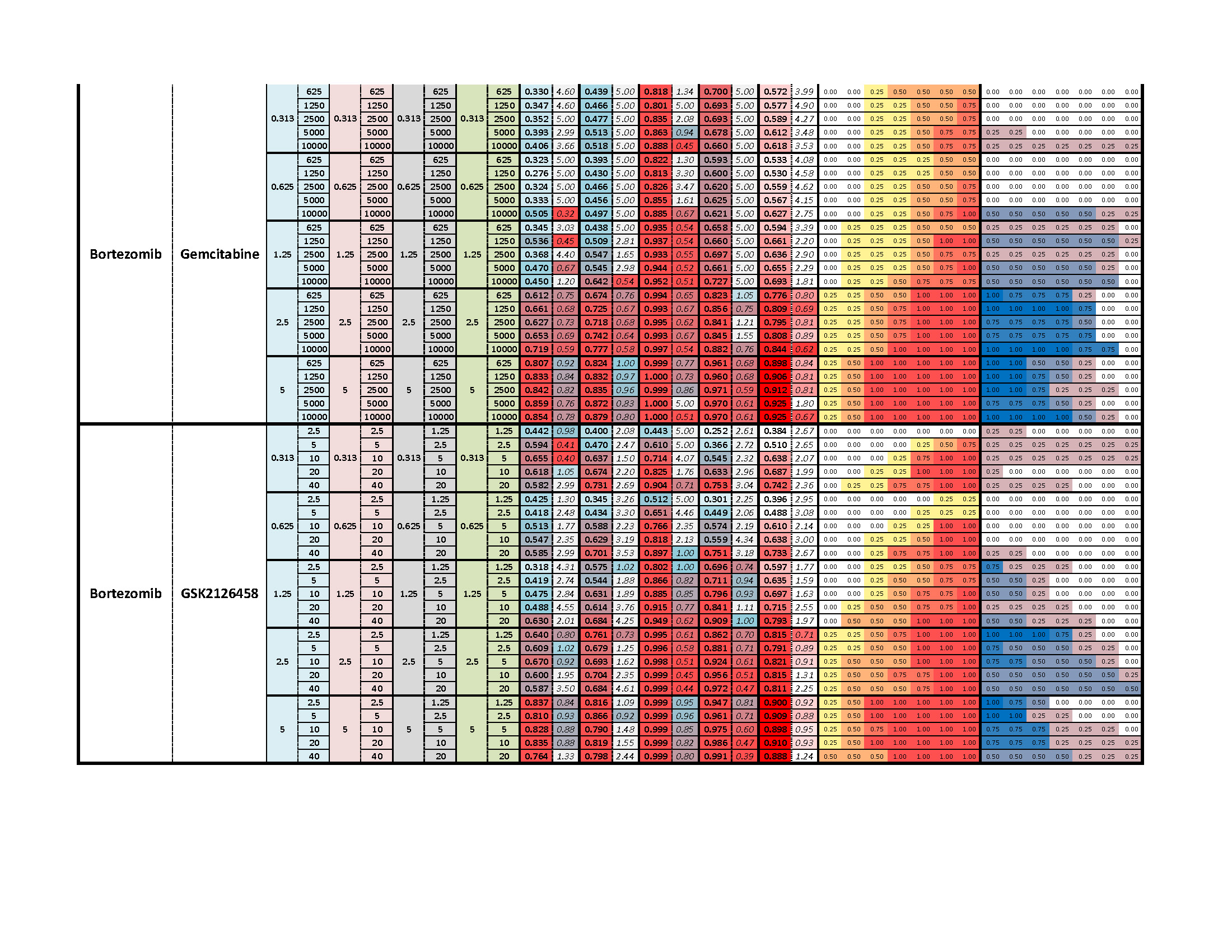


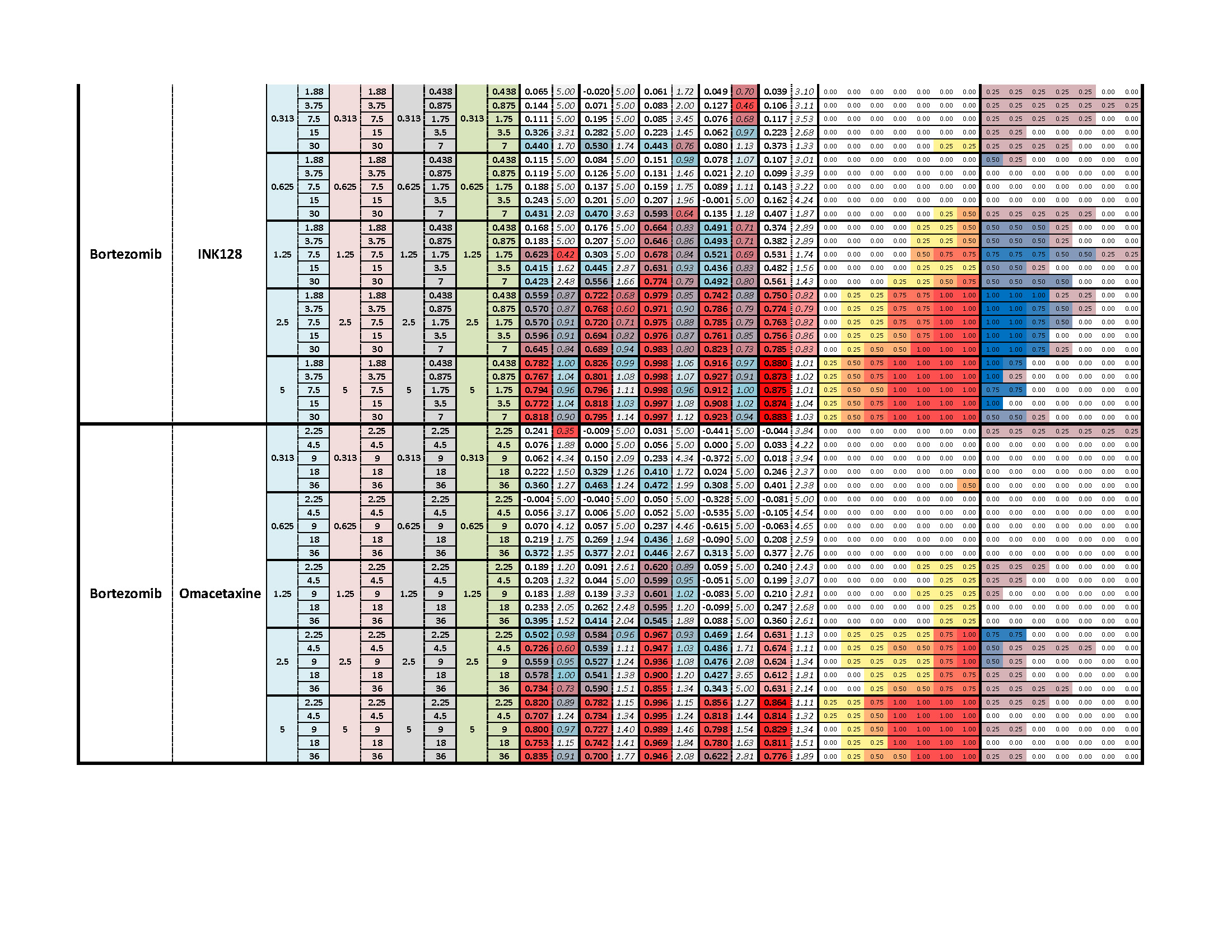

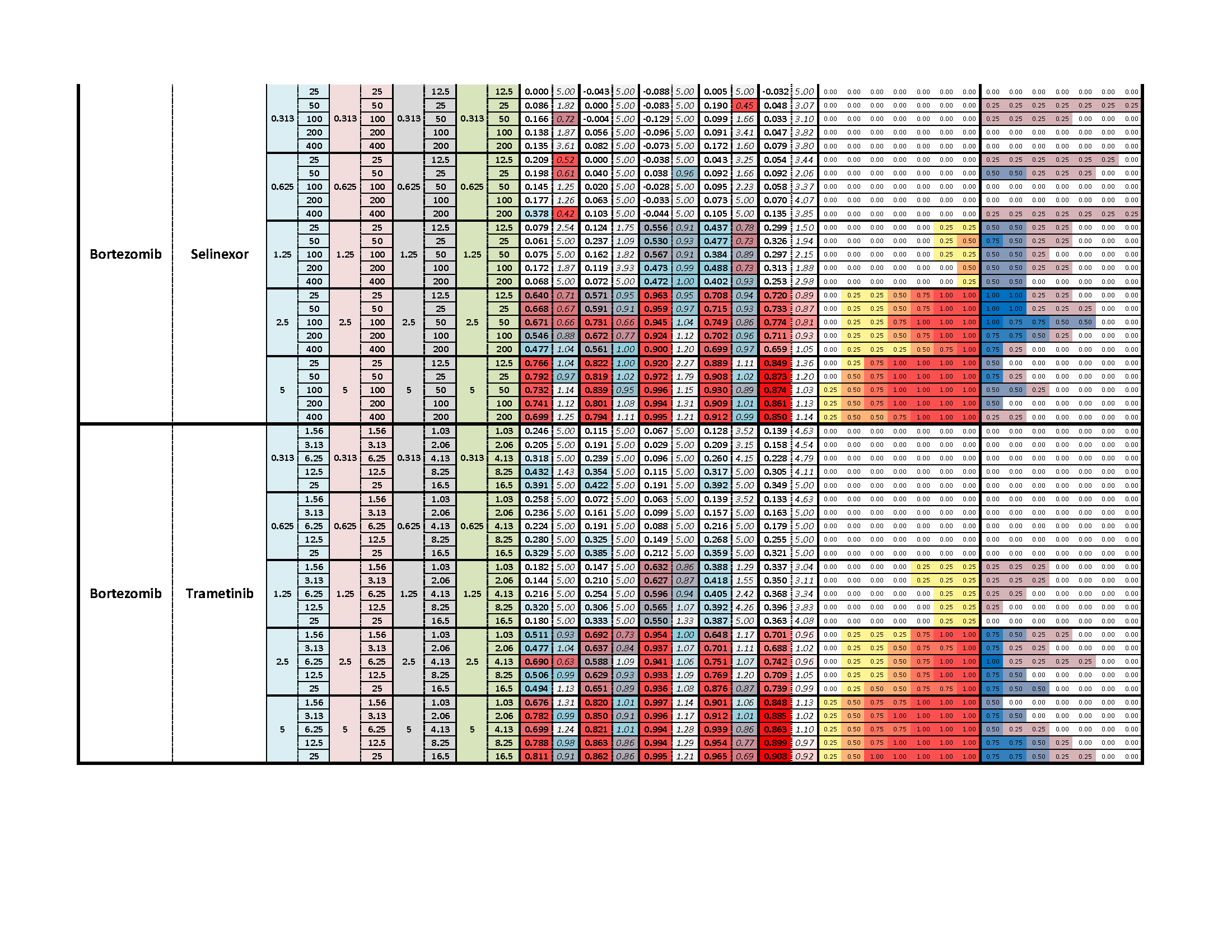


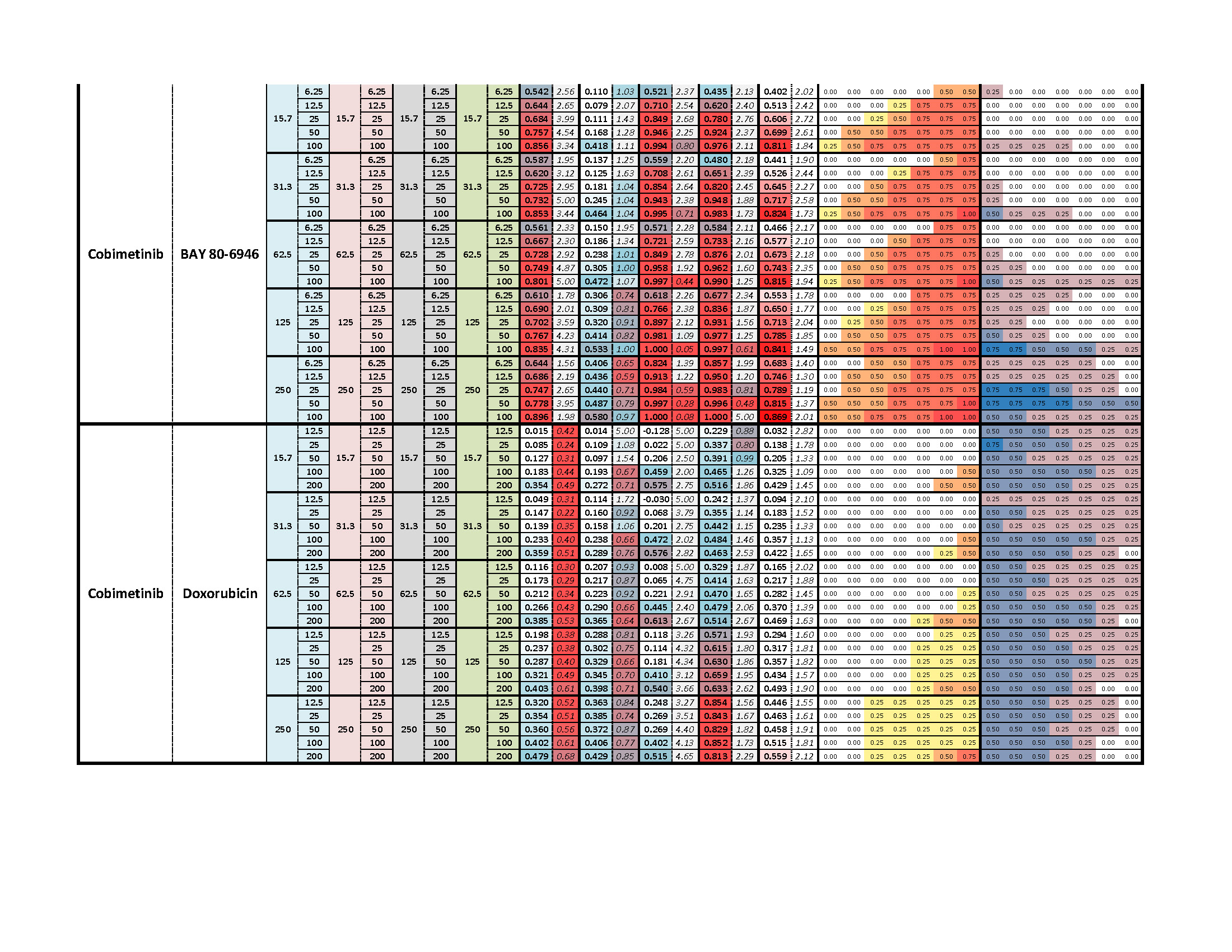

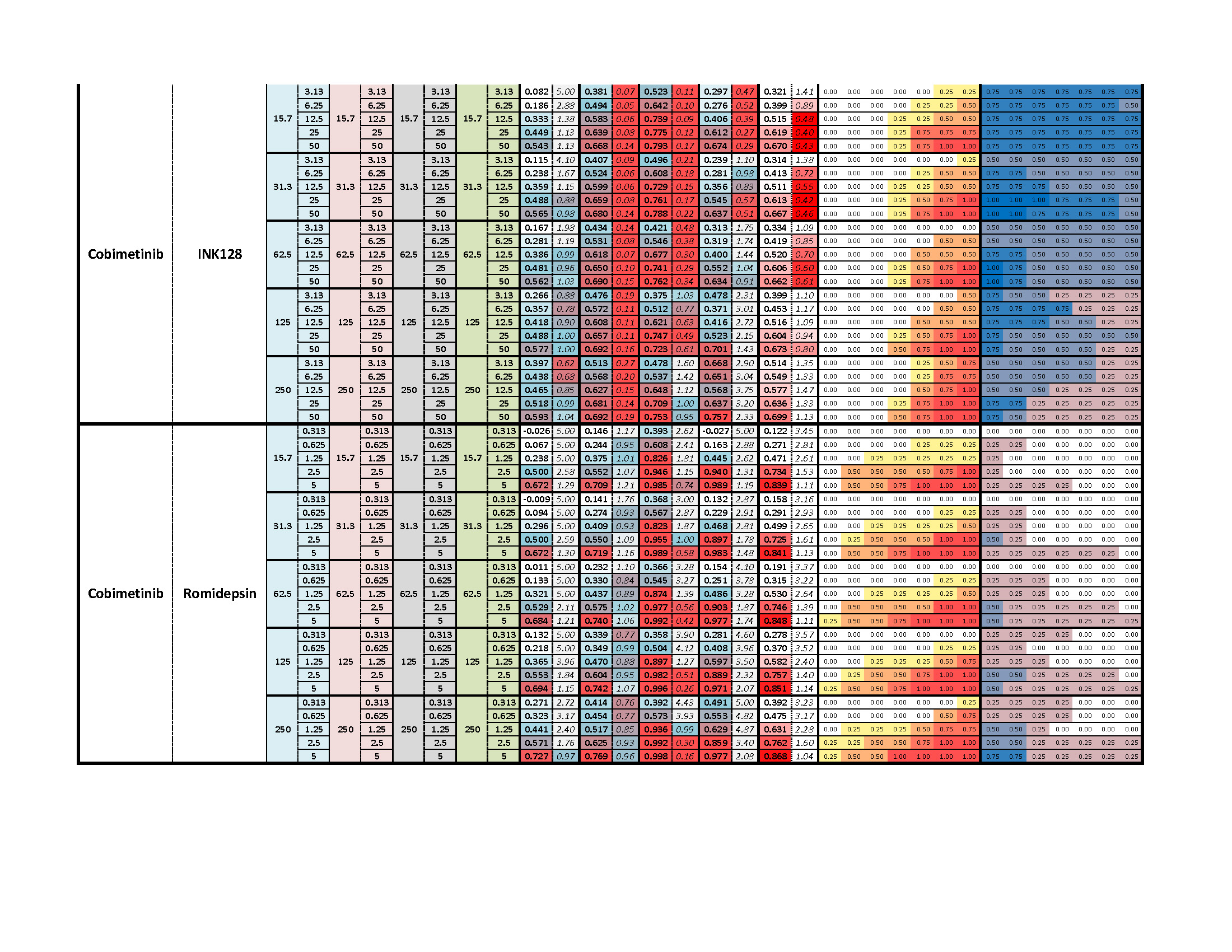


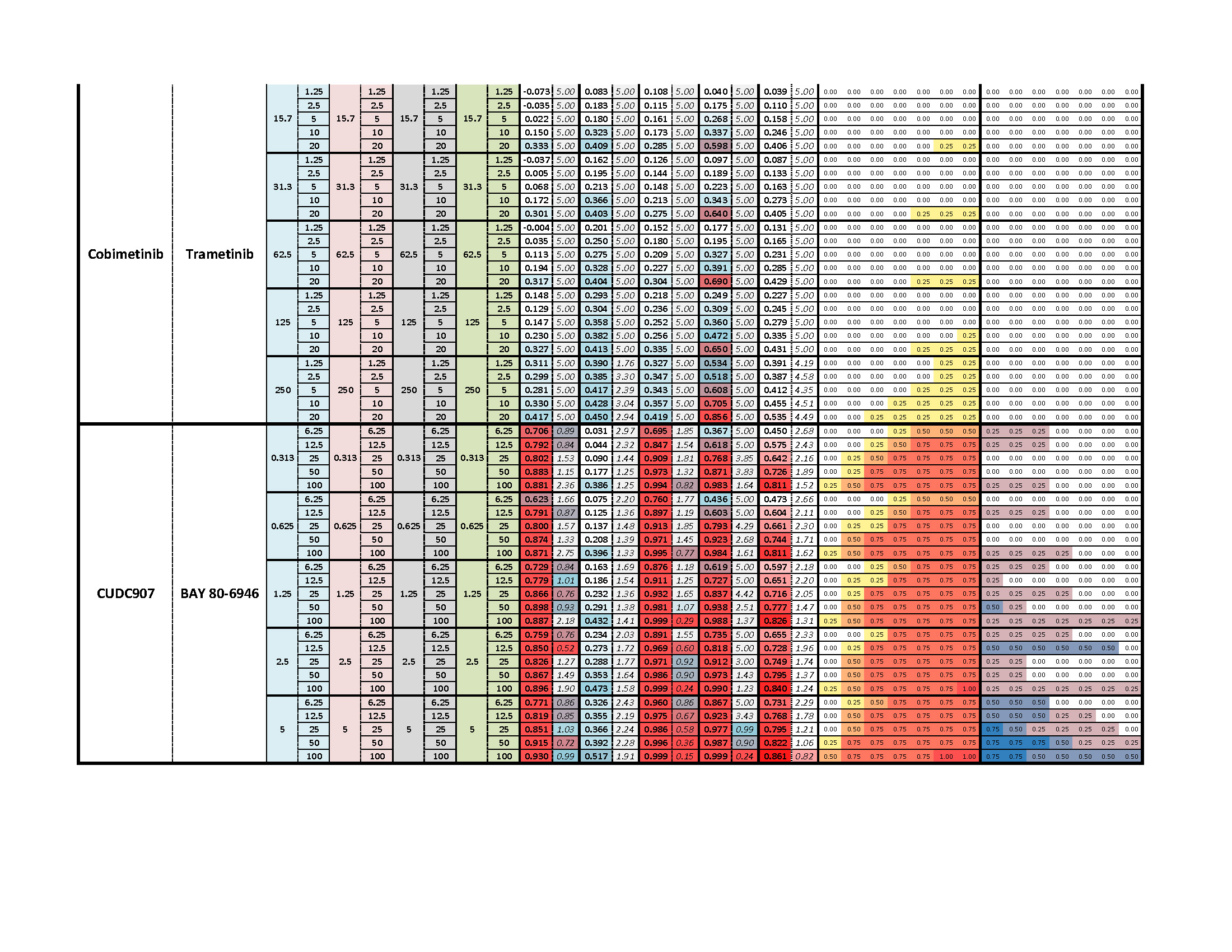

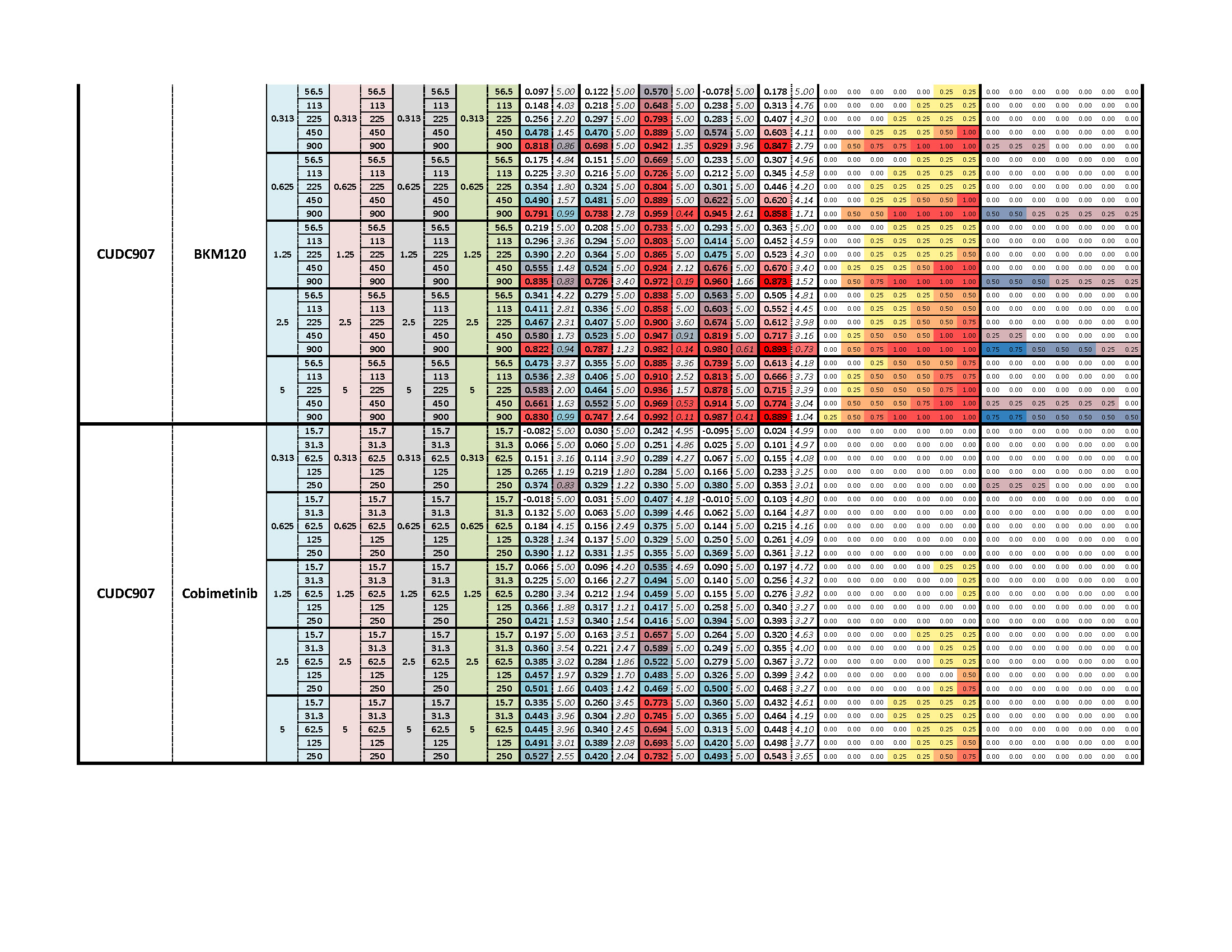


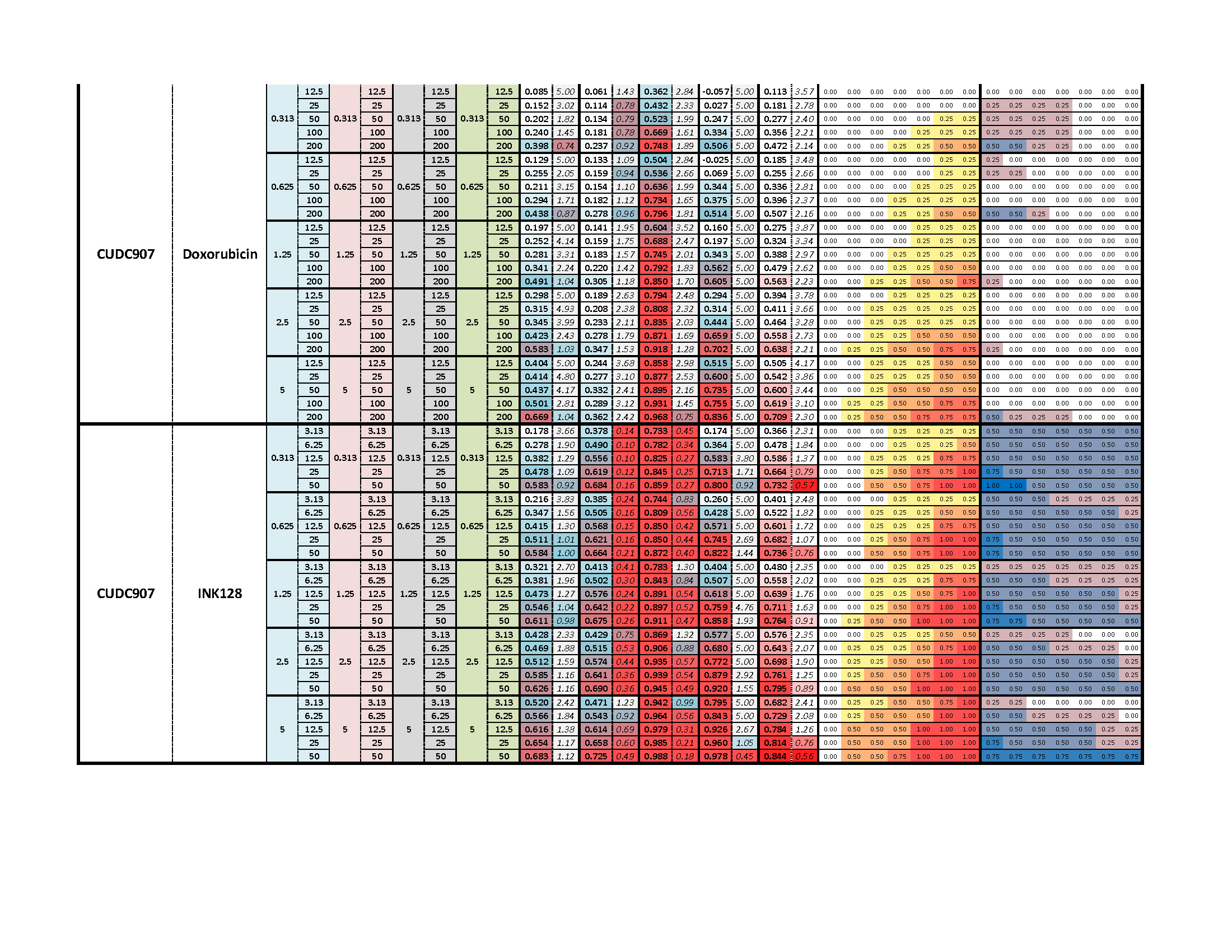

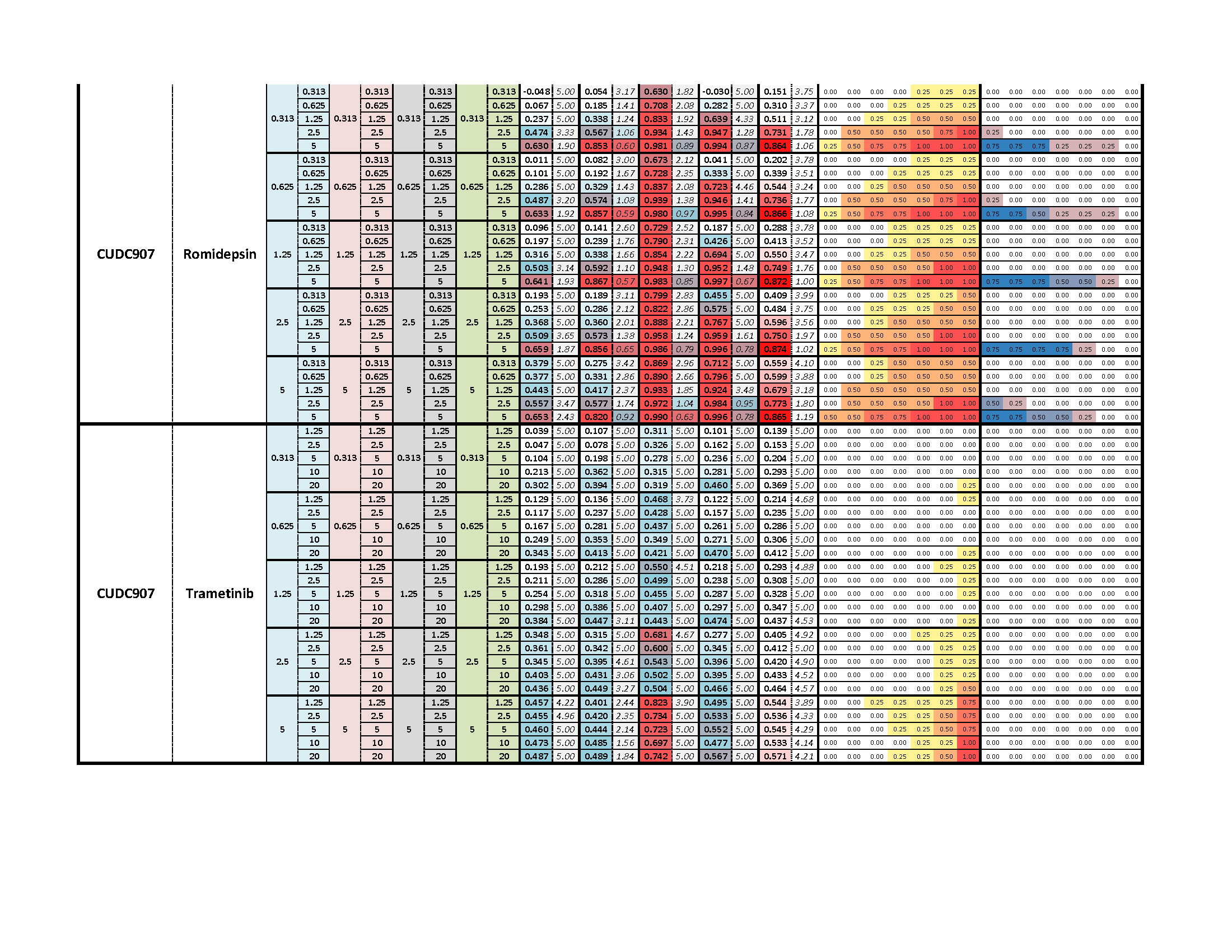


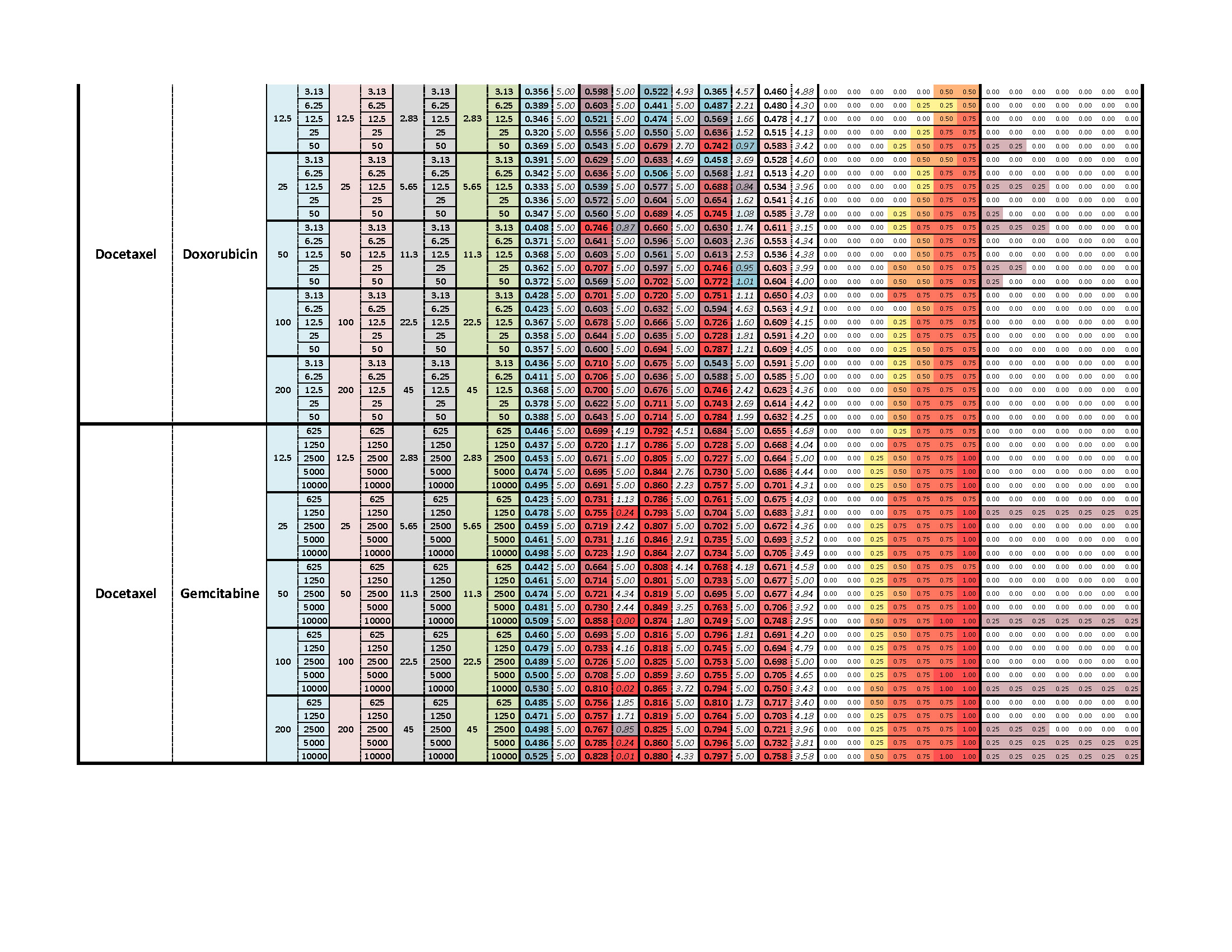

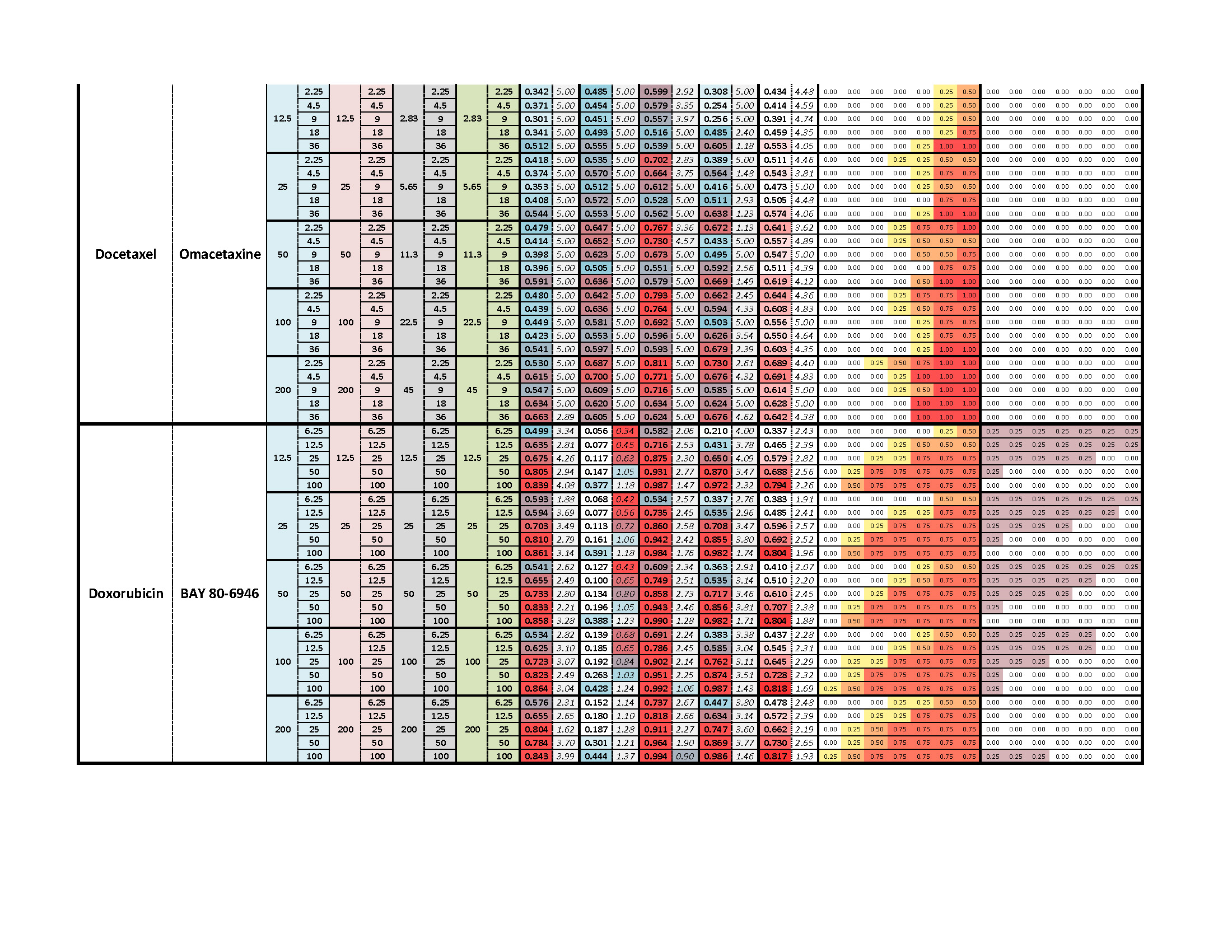


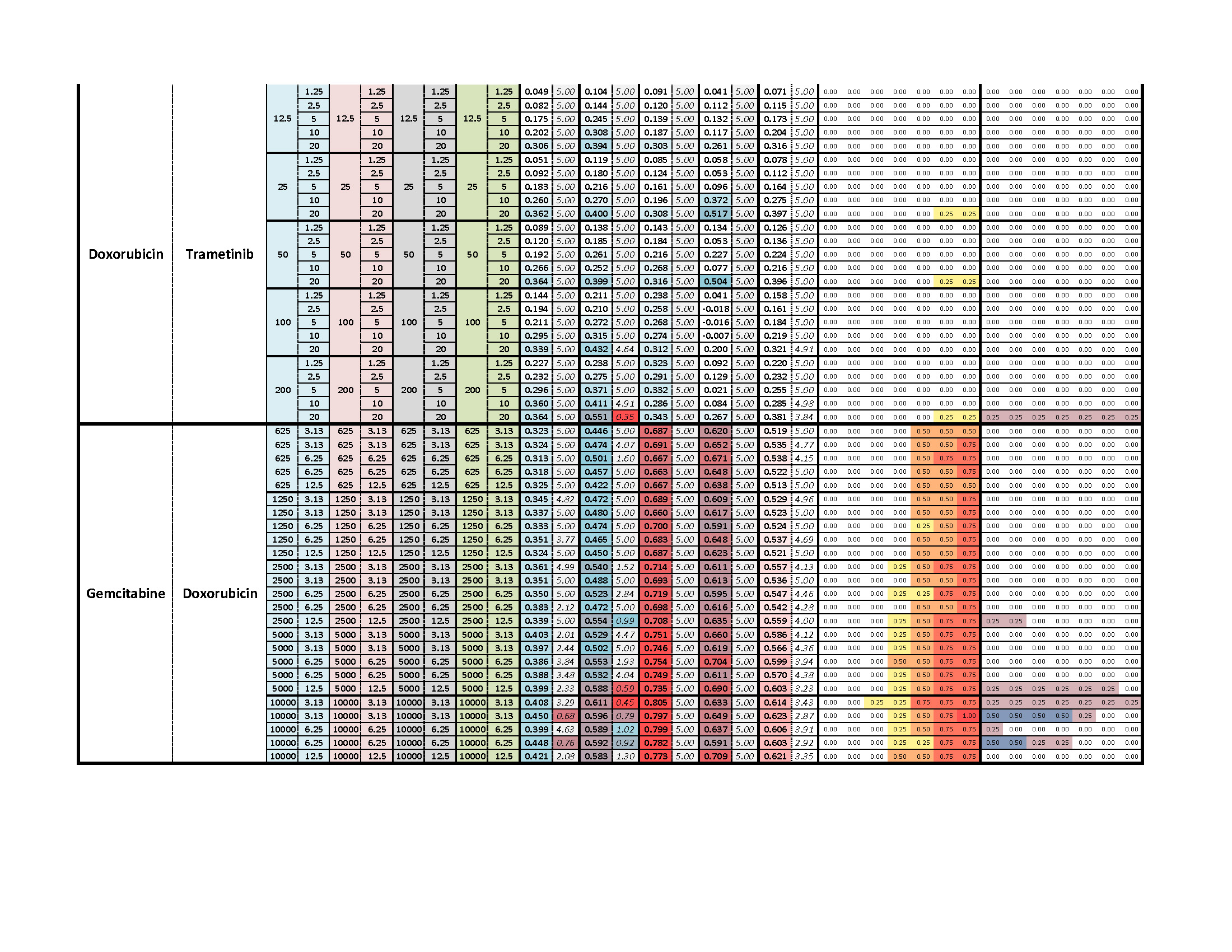

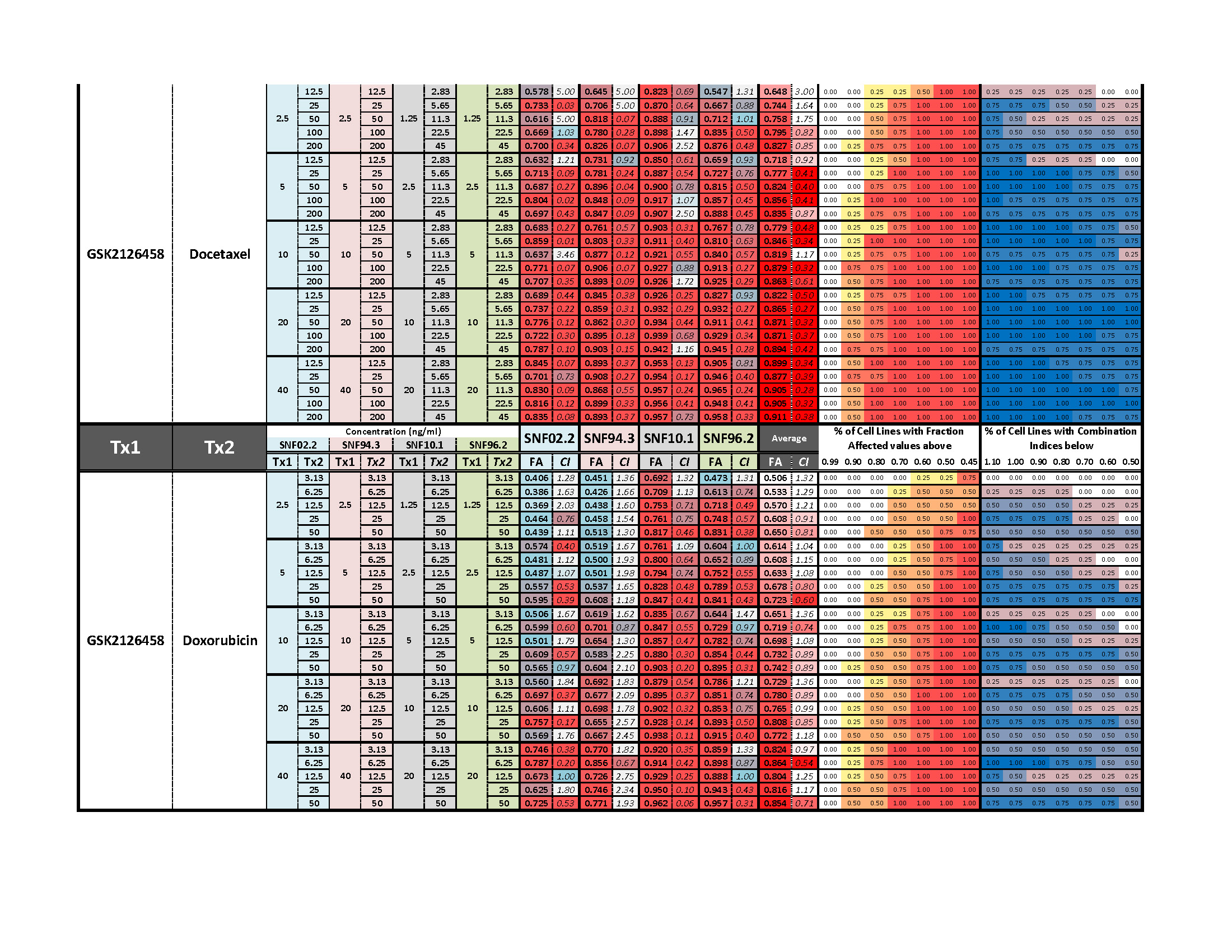


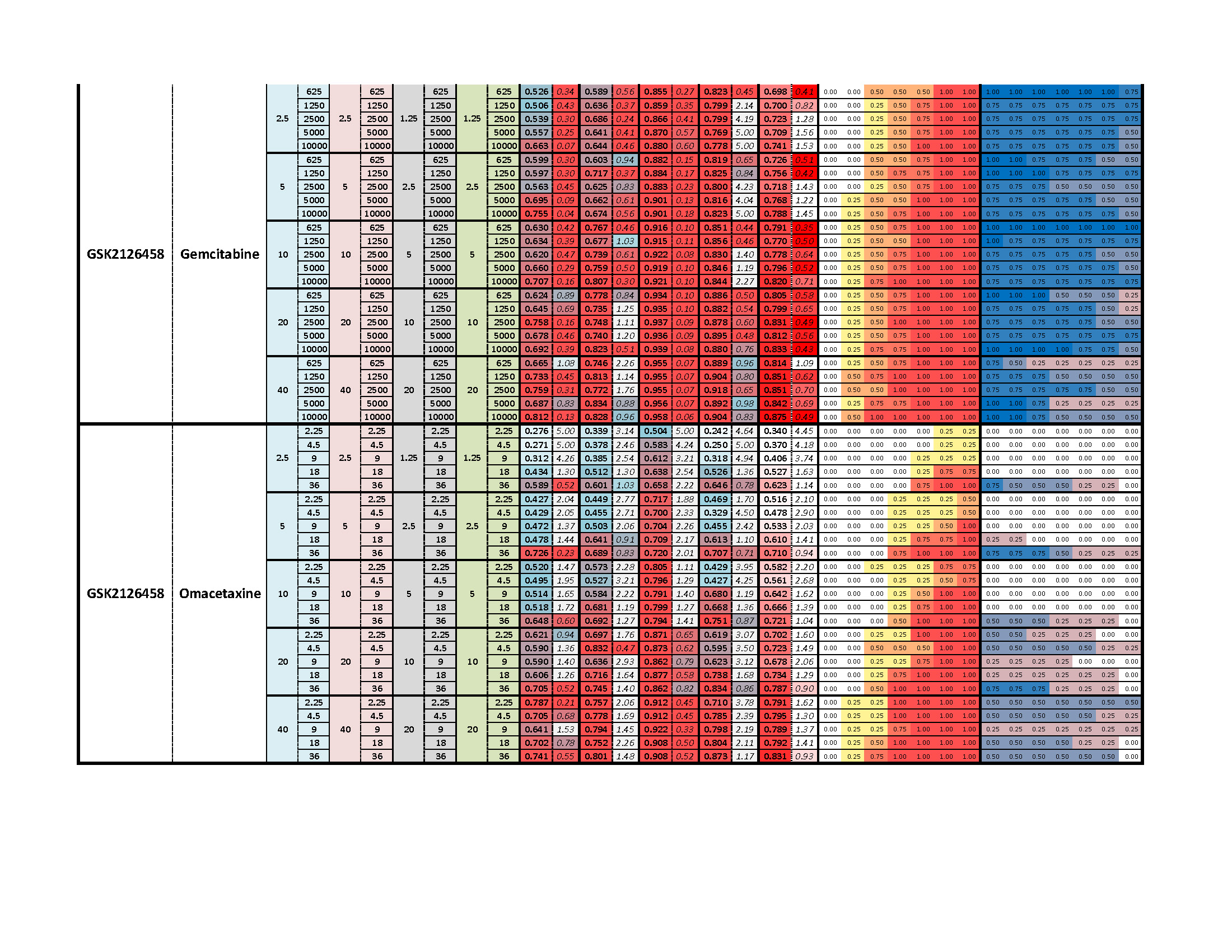

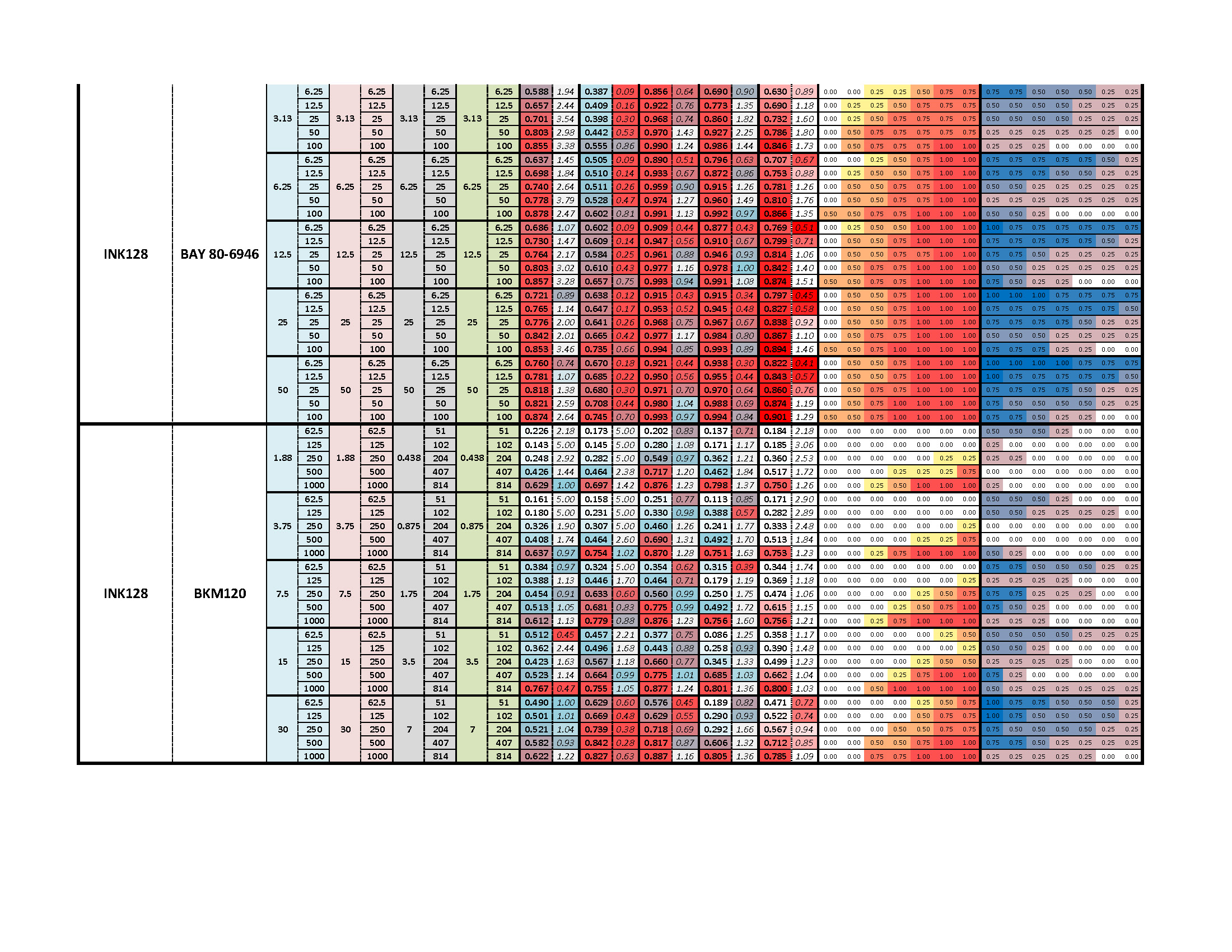


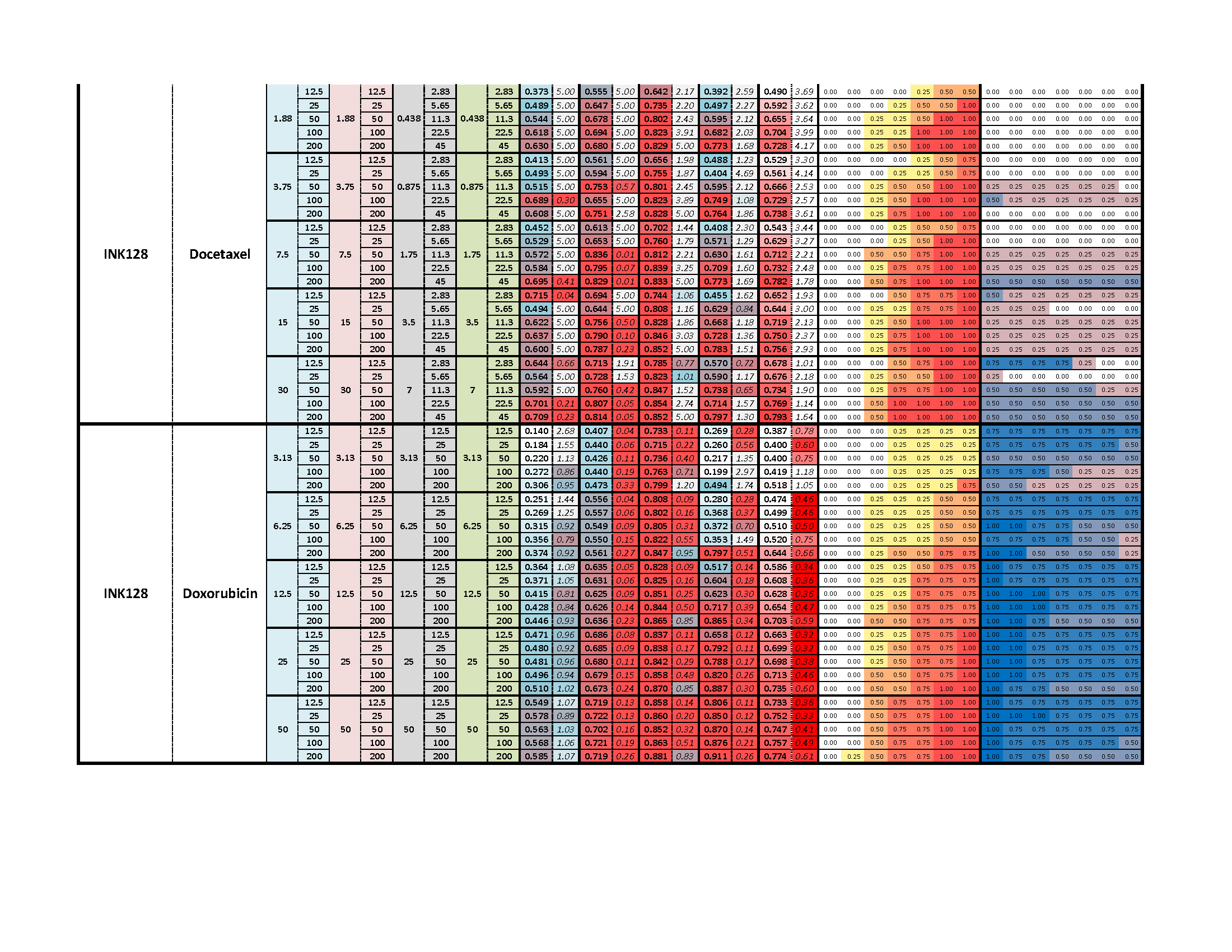

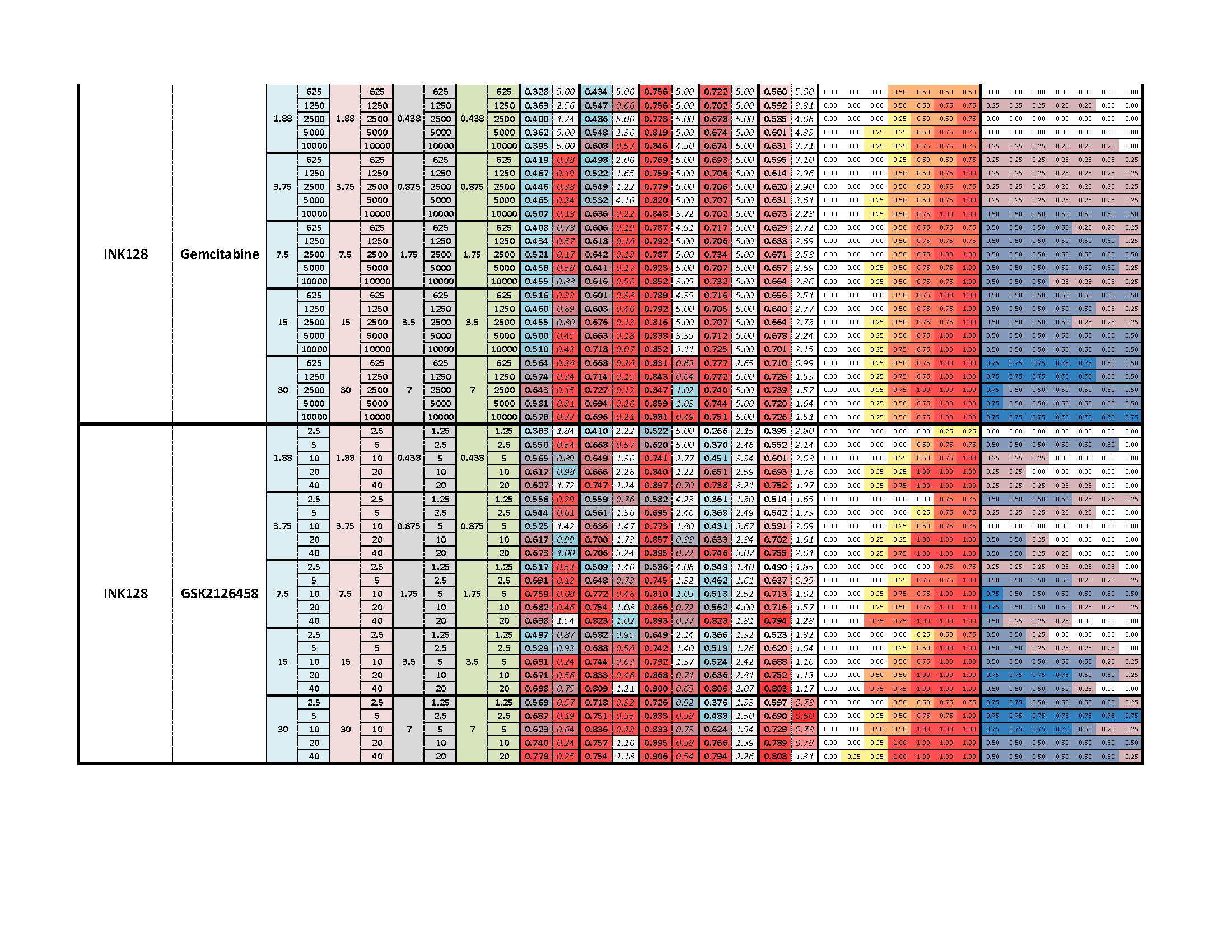


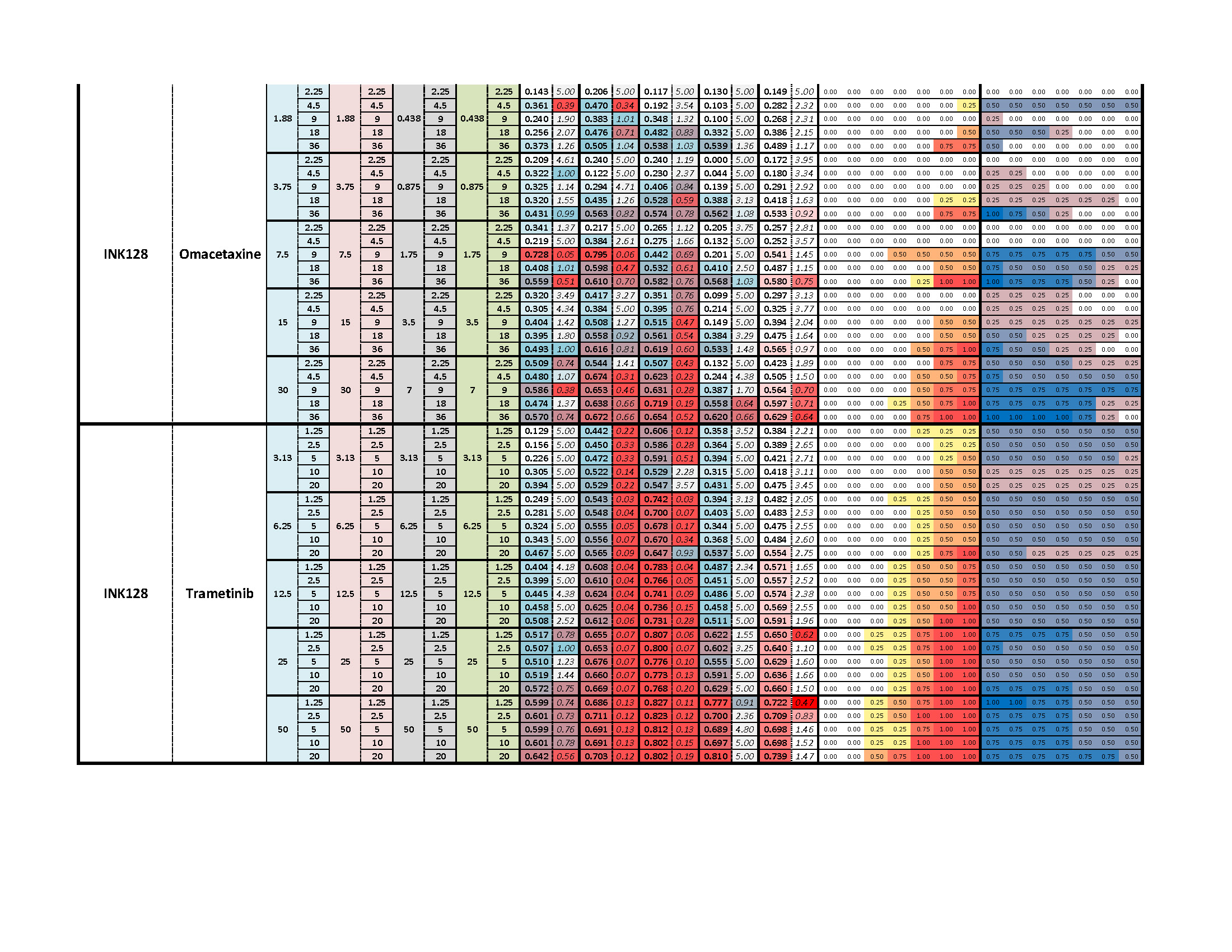

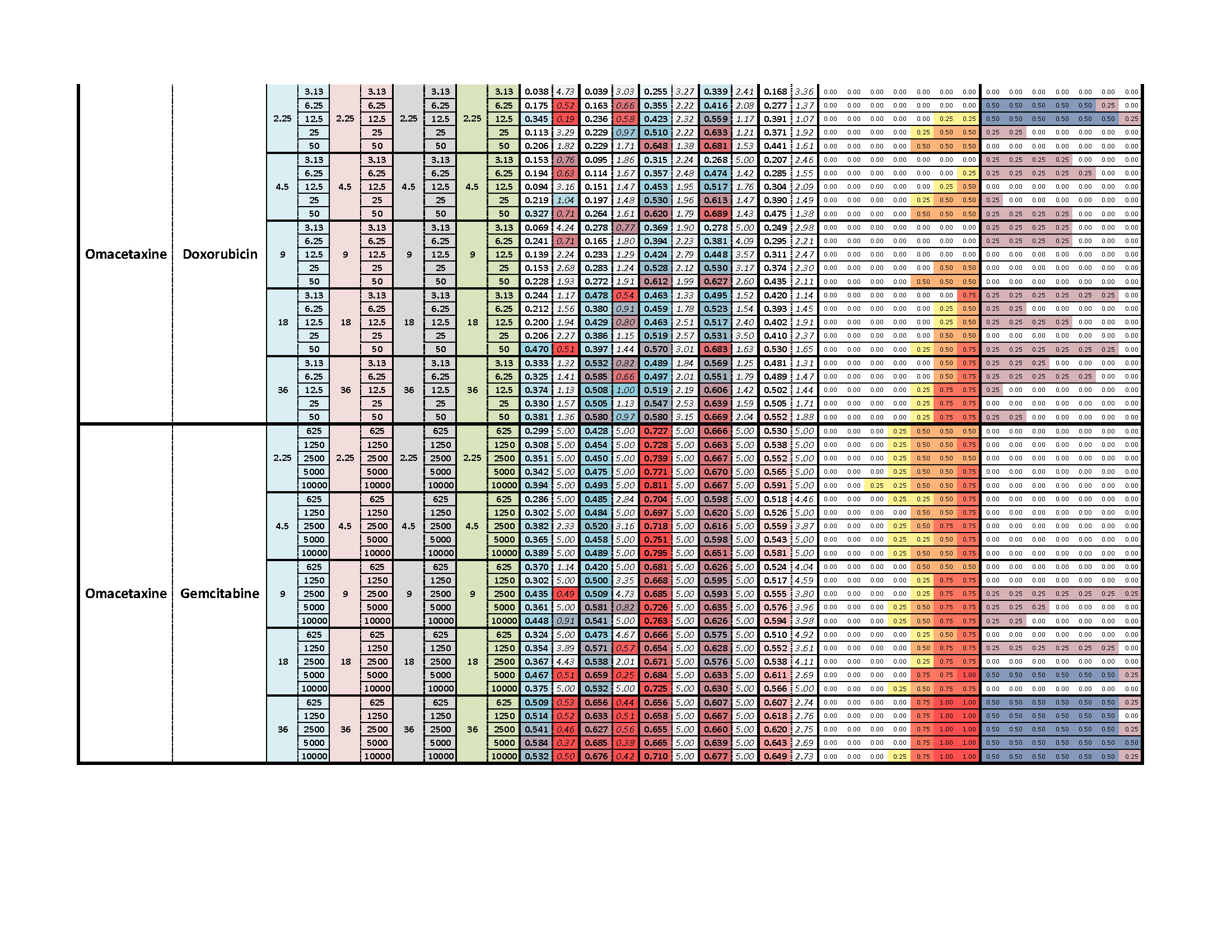


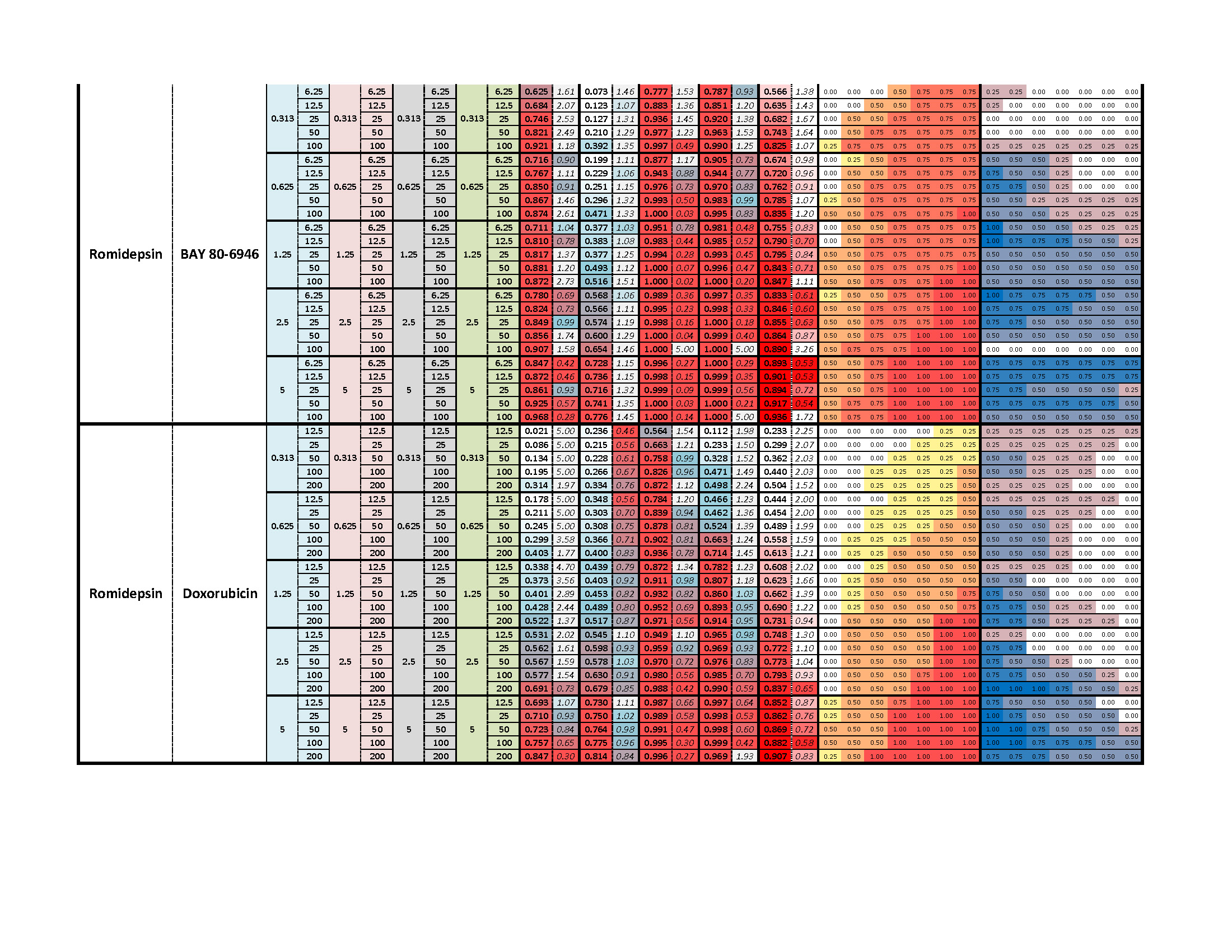

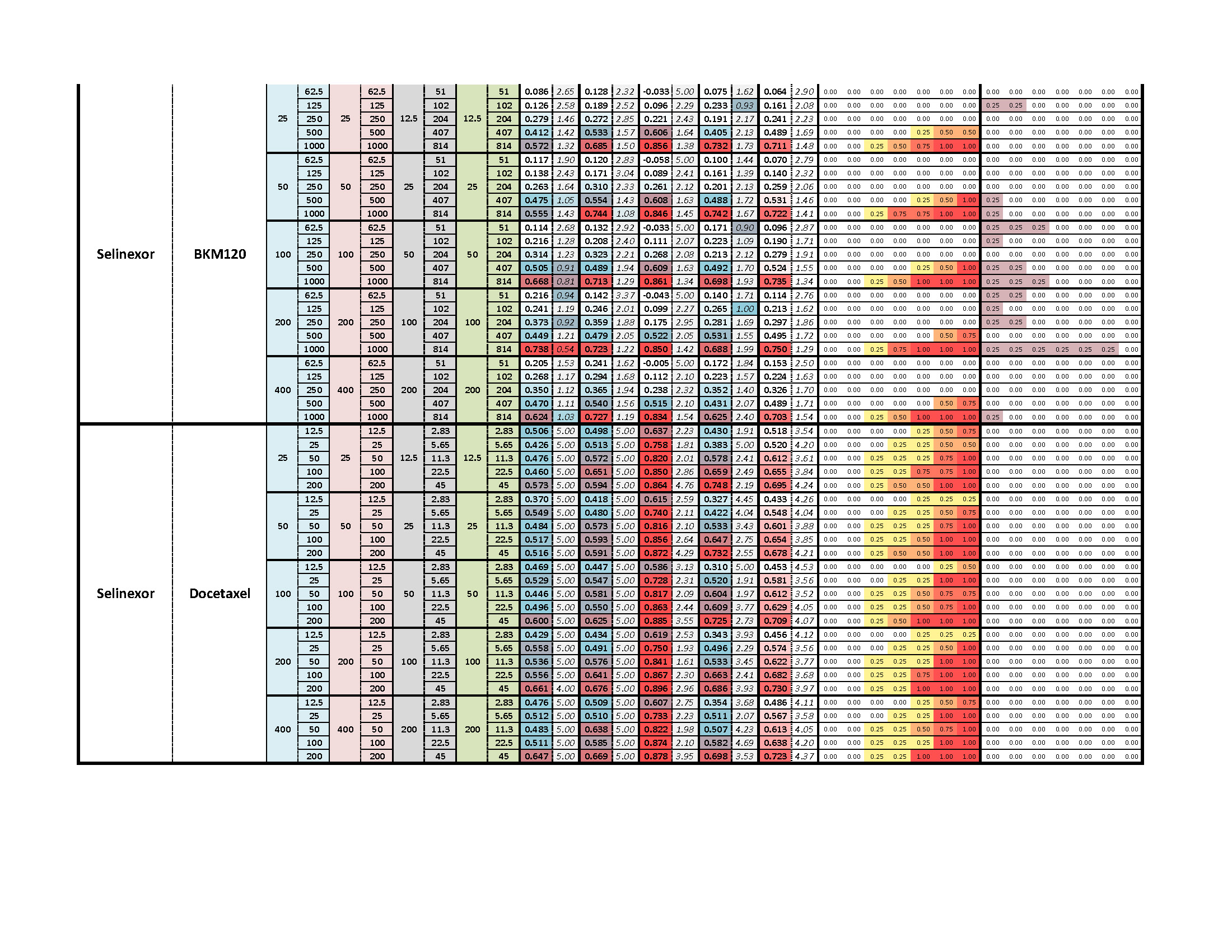

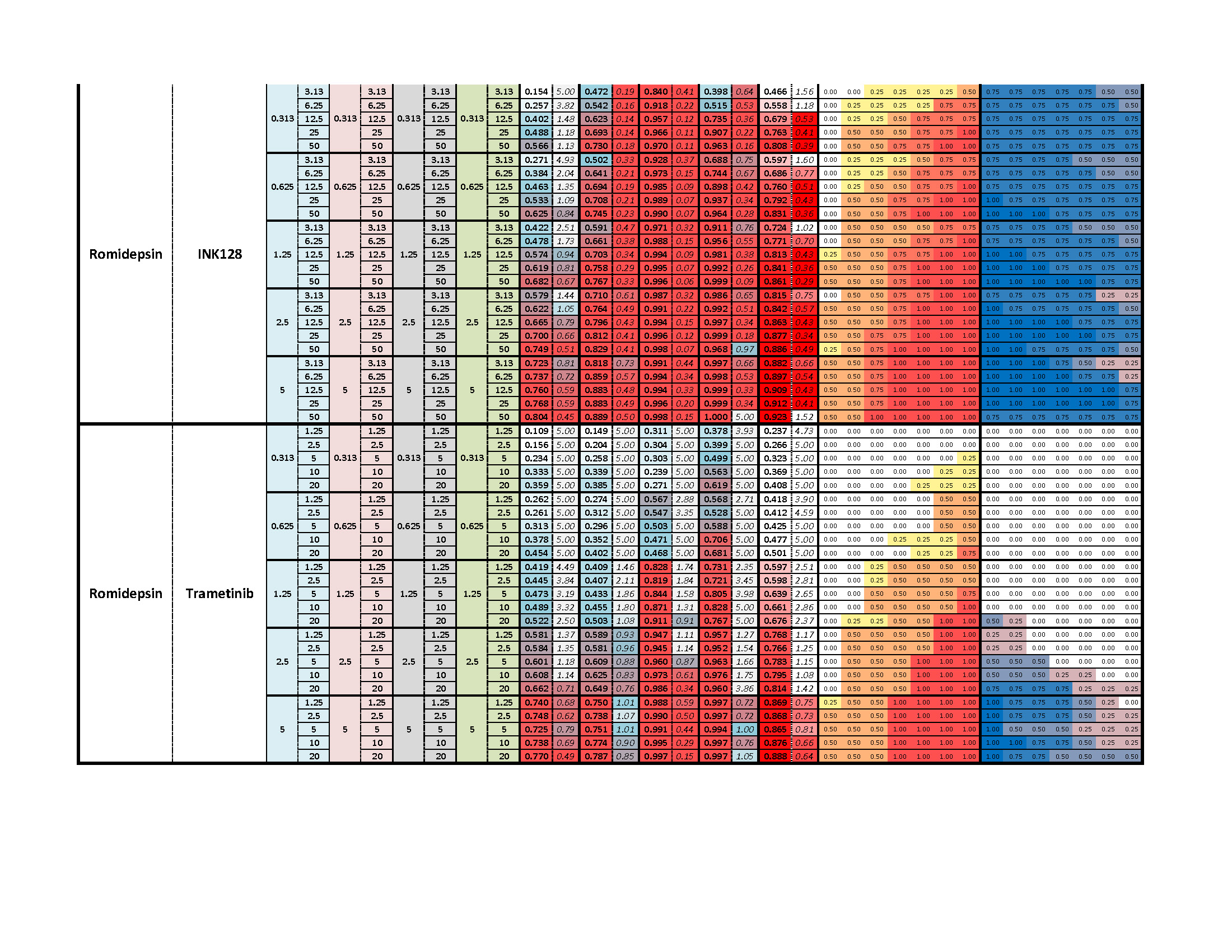


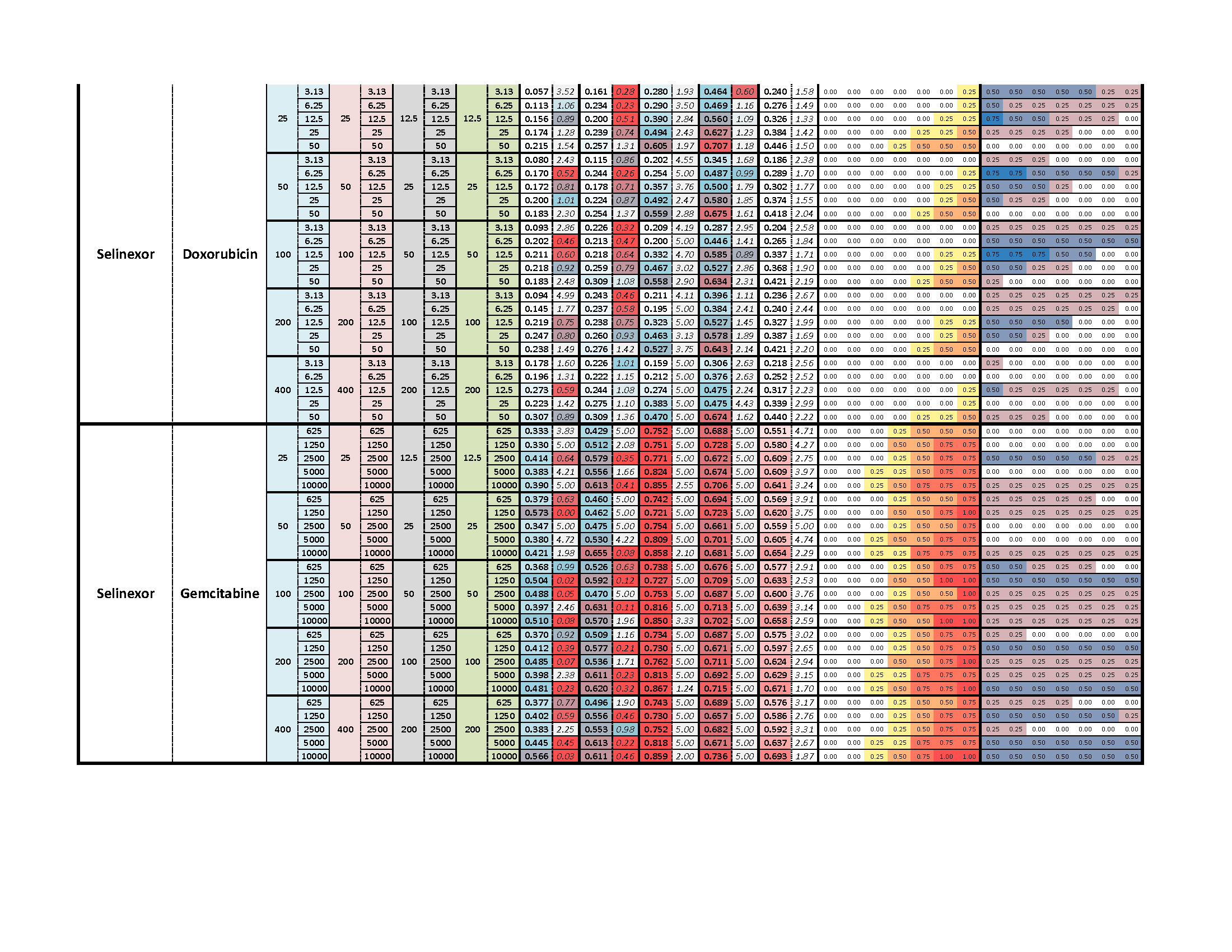

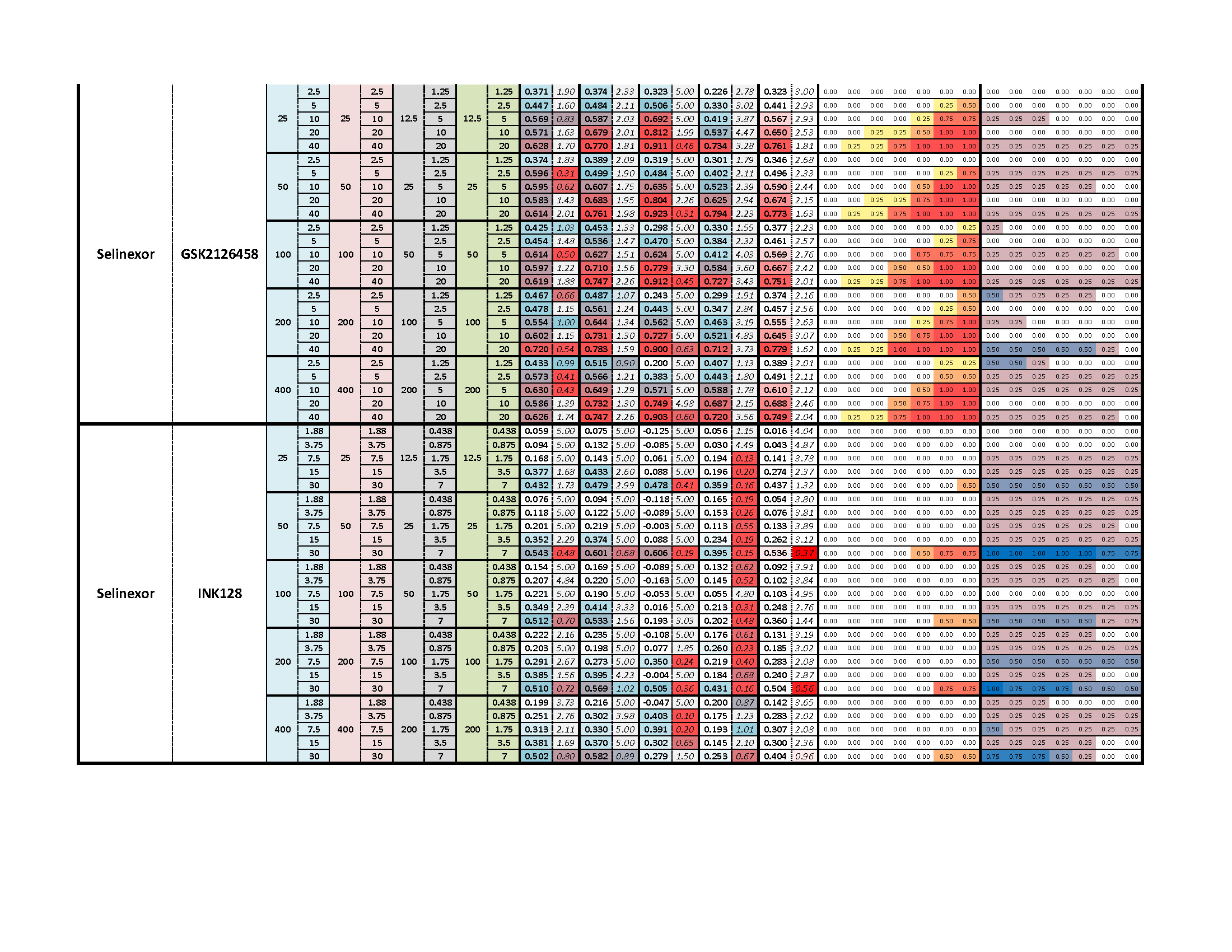


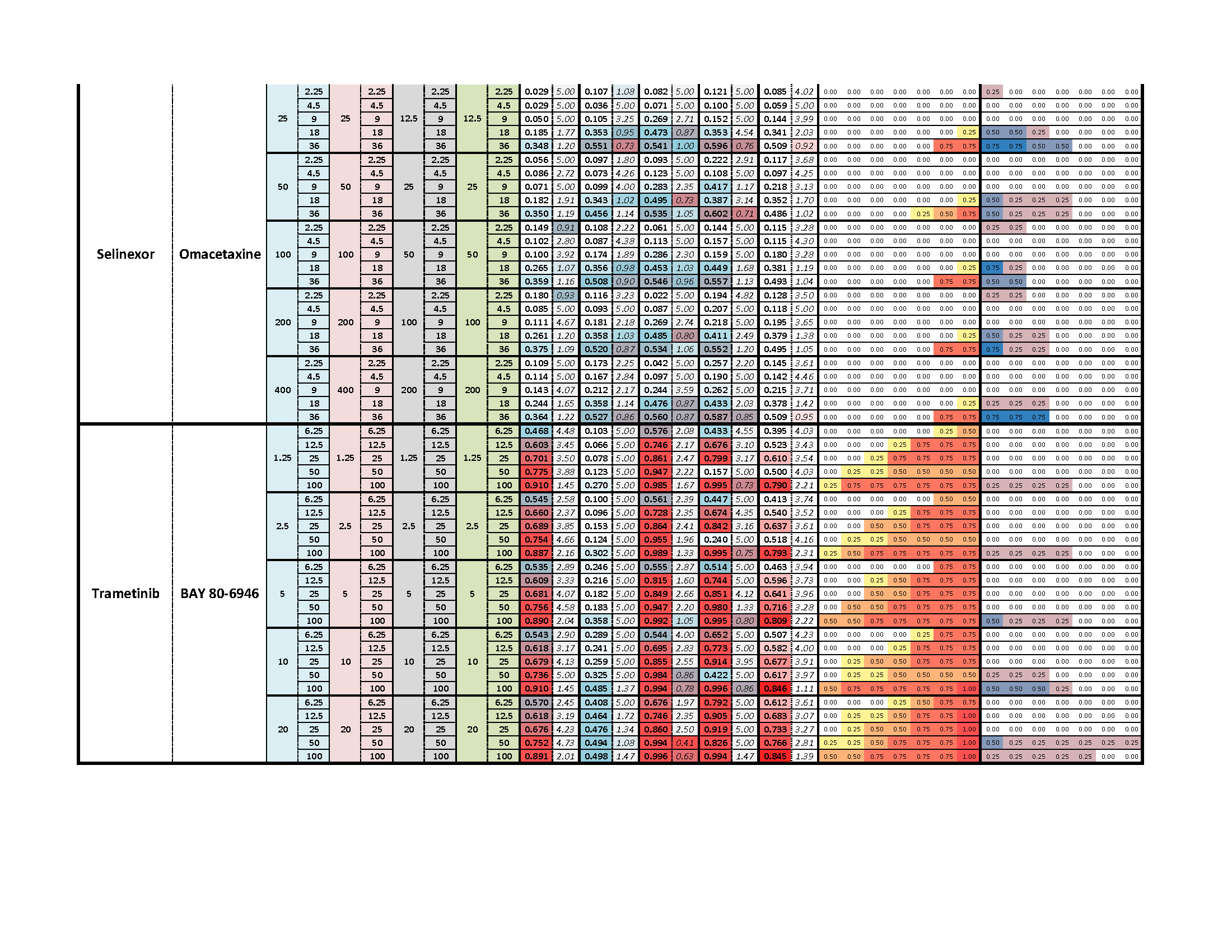

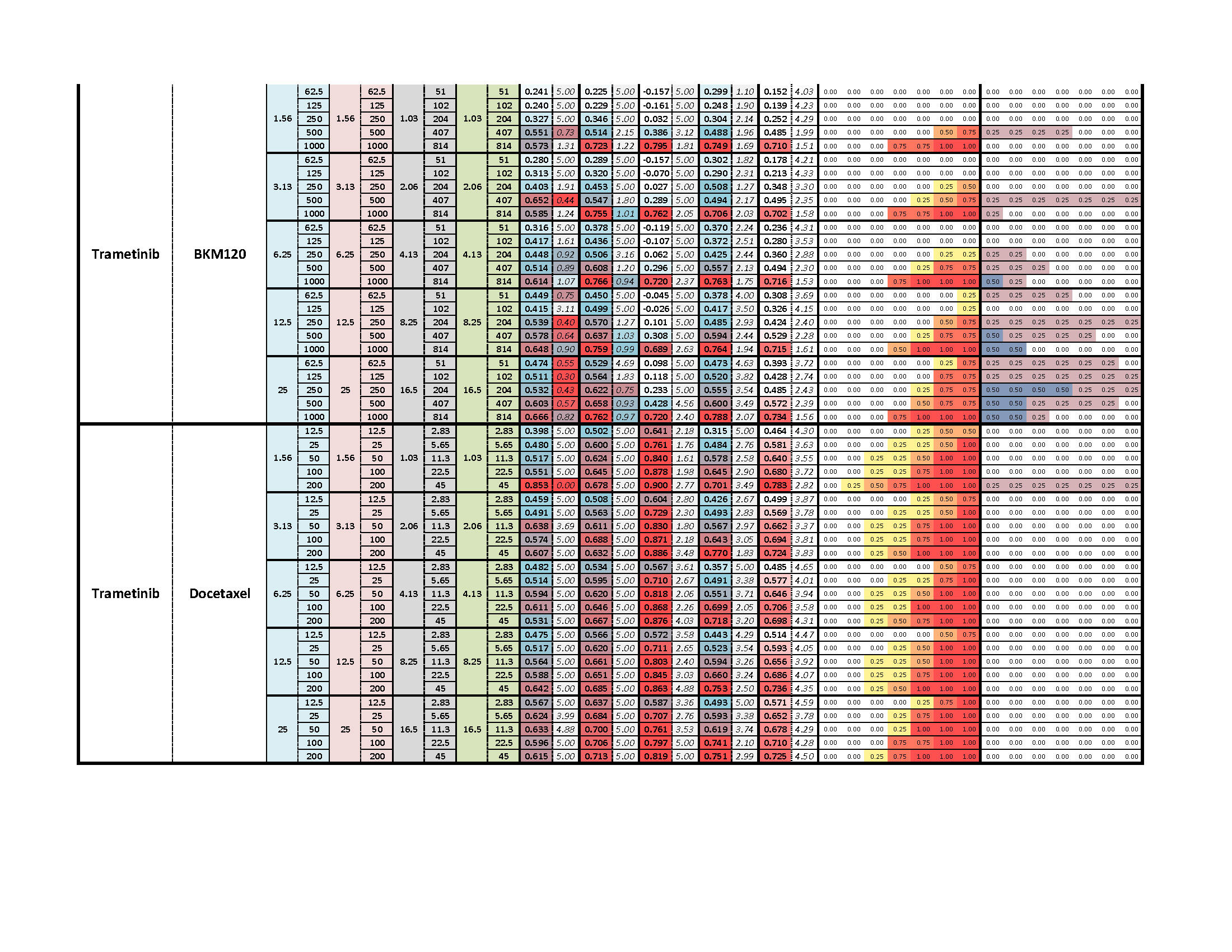


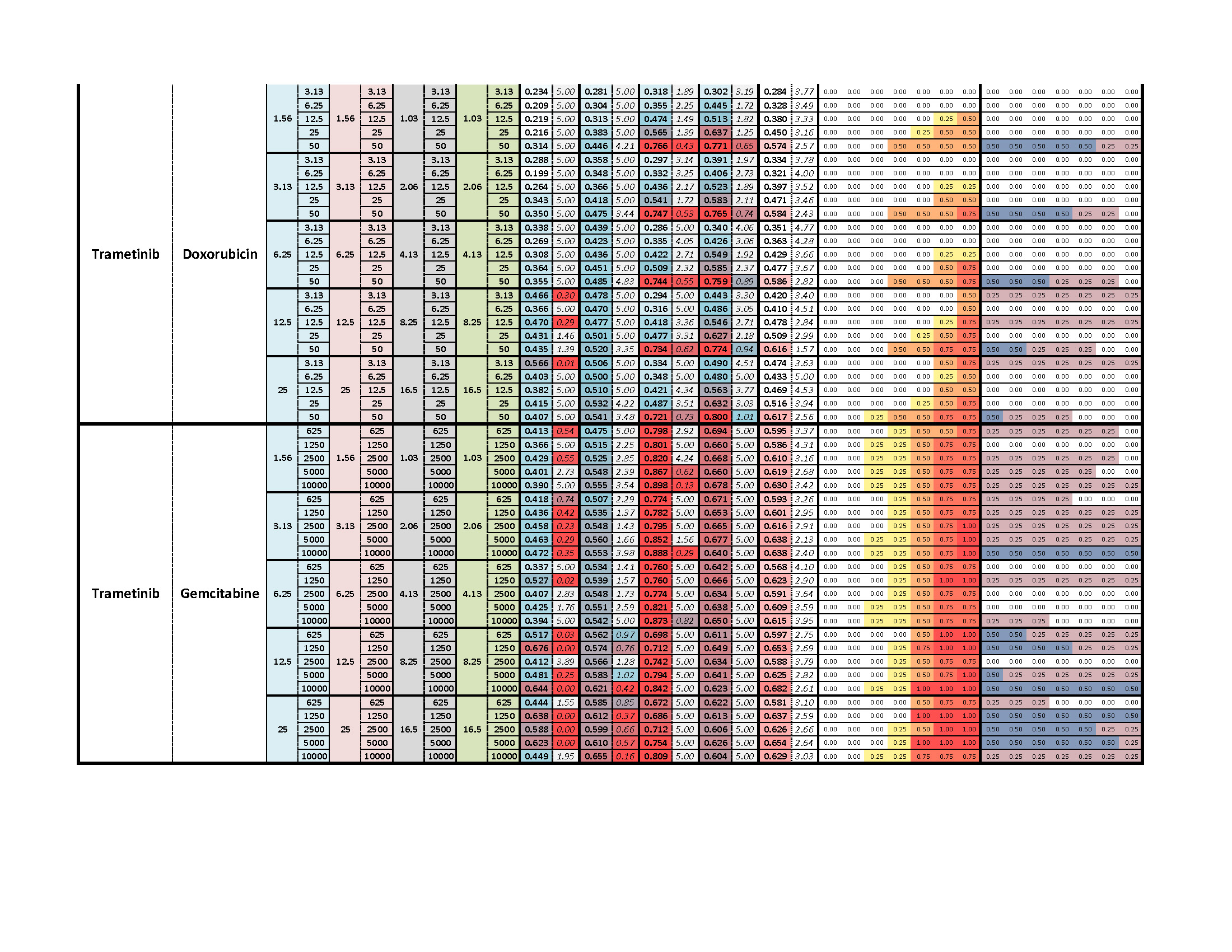

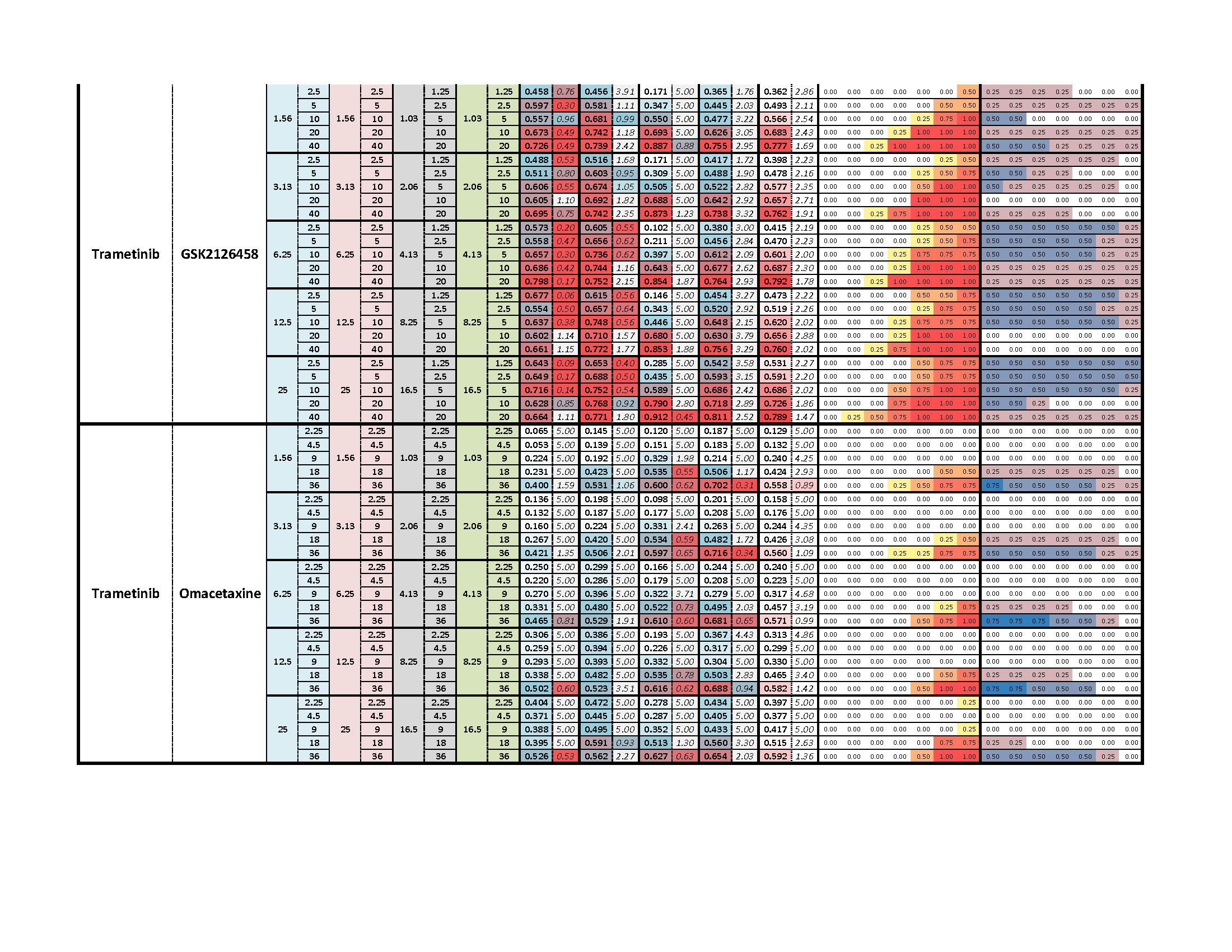


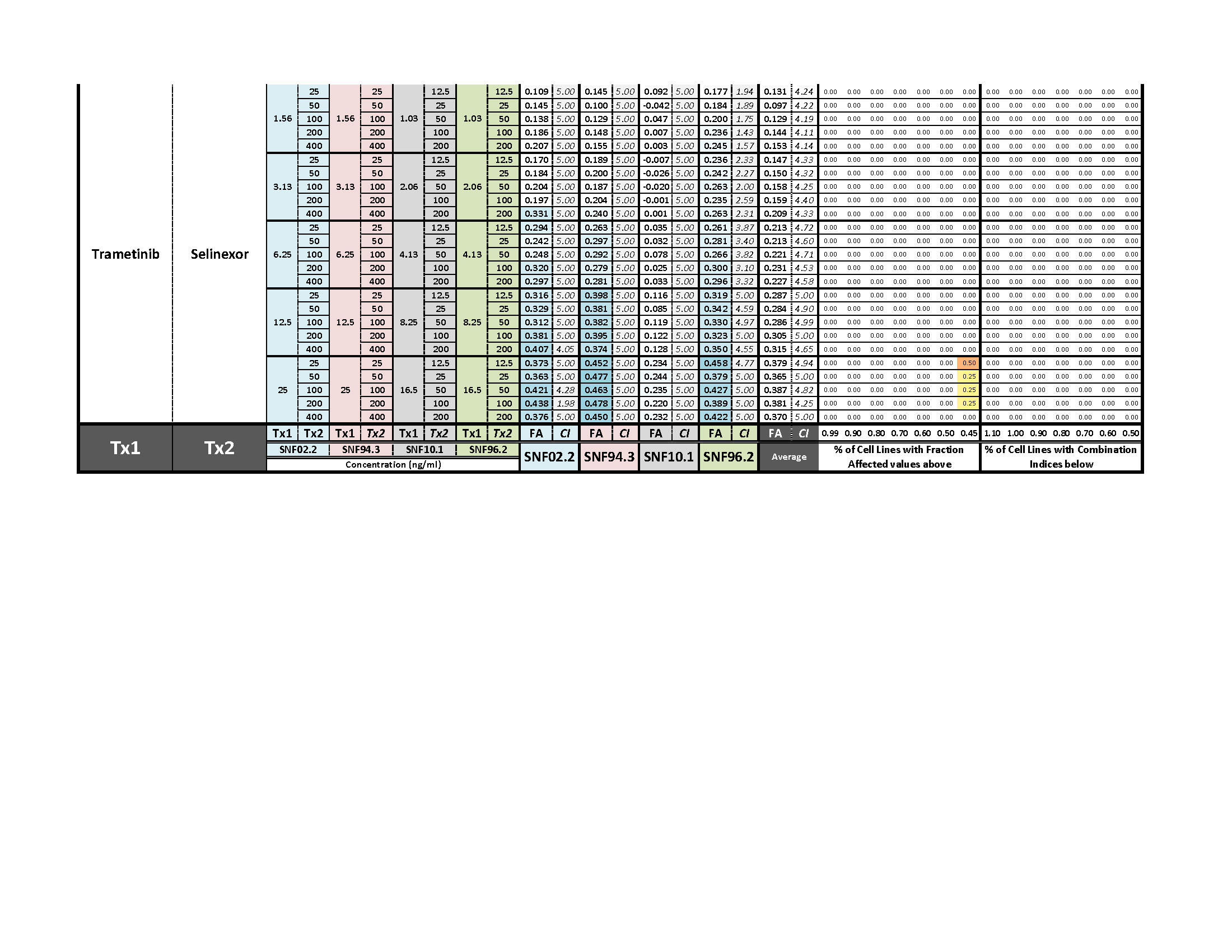

Supplement: Supplementary file 4 [file oncotarget-09-22571-s004.docx]
